# Neolithic farmers or Neolithic foragers? Organic residue analysis of early pottery from Rakushechny Yar on the Lower Don (Russia)

**DOI:** 10.1007/s12520-021-01412-2

**Published:** 2021-07-26

**Authors:** Manon Bondetti, Lara González Carretero, Ekaterina Dolbunova, Krista McGrath, Sam Presslee, Alexandre Lucquin, Viktor Tsybriy, Andrey Mazurkevich, Andrey Tsybriy, Peter Jordan, Carl Heron, John Meadows, Oliver E. Craig

**Affiliations:** 1 BioArCh, University of York, Environment Building, Wentworth Way Heslington, York, YO10 5DD UK; 2 Arctic Centre and Groningen Institute of Archaeology (GIA), University of Groningen, Aweg 30, 9718CW Groningen, The Netherlands; 3 Department of Scientific Research, The British Museum, London, WC1B 3DG UK; 4 Department of Archaeology of Eastern Europe and Siberia, The State Hermitage Museum, 34 Dvortsovaya Embankment, Saint Petersburg, 190000 Russian Federation; 5 ICTA, Universitate Autonoma de Barcelona, UAB 08193 Bellaterra (Cerdanyola), Building Z Campus, Barcelona, Spain; 6 Don Archaeological Society, 95A M Gorkogo, Rostov-on-Don, Russian Federation; 7 Department of Archaeology and Ancient History, Lund University, Lund, Sweden; 8 Centre for Baltic and Scandinavian Archaeology (ZBSA), SchlossGottorf, Schleswig-Holstein State Museums Foundation, Schlossinsel 1, 24837 Schleswig, Germany

**Keywords:** **P**ottery, Early Neolithic hunter-gatherer, Farmers, Lipid residue analysis, ZooMS, Scanning electron microscopy (SEM)

## Abstract

The emergence of pottery in Europe is associated with two distinct traditions: hunter-gatherers in the east of the continent during the early 6th millennium BC and early agricultural communities in the south-west in the late 7th millennium BC. Here we investigate the function of pottery from the site of Rakushechny Yar, located at the Southern fringe of Eastern Europe, in this putative contact zone between these two economic ‘worlds’. To investigate, organic residue analysis was conducted on 120 samples from the Early Neolithic phase (ca. mid-6^th^ millennium BC) along with microscopic and SEM analysis of associated foodcrusts. The results showed that the earliest phase of pottery use was predominantly used to process riverine resources. Many of the vessels have molecular and isotopic characteristics consistent with migratory fish, such as sturgeon, confirmed by the identification of sturgeon bony structures embedded in the charred surface deposits. There was no evidence of dairy products in any of the vessels, despite the fact these have been routinely identified in coeval sites to the south. Further analysis of some of the mammalian bones using ZooMS failed to demonstrate that domesticated animals were present in the Early Neolithic. Nevertheless, we argue that intensive exploitation of seasonally migratory fish, accompanied by large-scale pottery production, created storable surpluses that led to similar socio-economic outcomes as documented in early agricultural societies.

**Supplementary Information:**

The online version contains supplementary material available at 10.1007/s12520-021-01412-2.

## Introduction

The transition to the Neolithic marks a period of profound socio-economic change, often related to increased sedentism, intensification of subsistence practices with the rise of new, more specialist strategies. Yet the economic transition that underpins this process has been interpreted differently. In the Western European archaeological tradition, ‘Neolithisation’ specifically refers to the economic transition from foraging to agriculture and the rearing of domesticated livestock, and is often associated with the introduction of a package of innovations, including pottery (Dixon 1928; Childe 1951; Kuzmin 2013; Hommel 2014). To prehistorians working in northern Eurasia, however, the Neolithic denotes the emergence of pottery production invariably amongst hunter-gatherer communities (Zhukov 1929; Chard 1974; Barnes 1999; Gibbs and Jordan 2016). For the latter, the degree to which the inception of pottery transformed hunter-gatherer subsistence practices is debated (Jordan and Zvelebil 2009). This debate has recently been fuelled by chemical analysis of early hunter-gatherer ceramic vessels allowing determination of their use. Based on evidence from Northern Europe and East Asia, it appears that aquatic foods were preferentially cooked in hunter-gatherer vessels, perhaps marking intensification of fishing at this time, leading to surplus production and creating the conditions for increased sedentism and population growth (e.g. Craig et al. 2007, 2011, 2013; Bērziņš 2010; Lucquin et al. 2016b, 2018; Oras et al. 2017; Shoda et al. 2017; Gibbs et al. 2017; Bondetti et al. 2020b). However, other studies have shown more variable patterns of use which, arguably, reflect economic continuity from the preceding ‘Mesolithic’ period (Oras et al. 2017; Bondetti et al. 2020b), where it is argued that intensified fishing was already established (e.g. Bērziņš 2010) or came later.

**Fig. 1:**
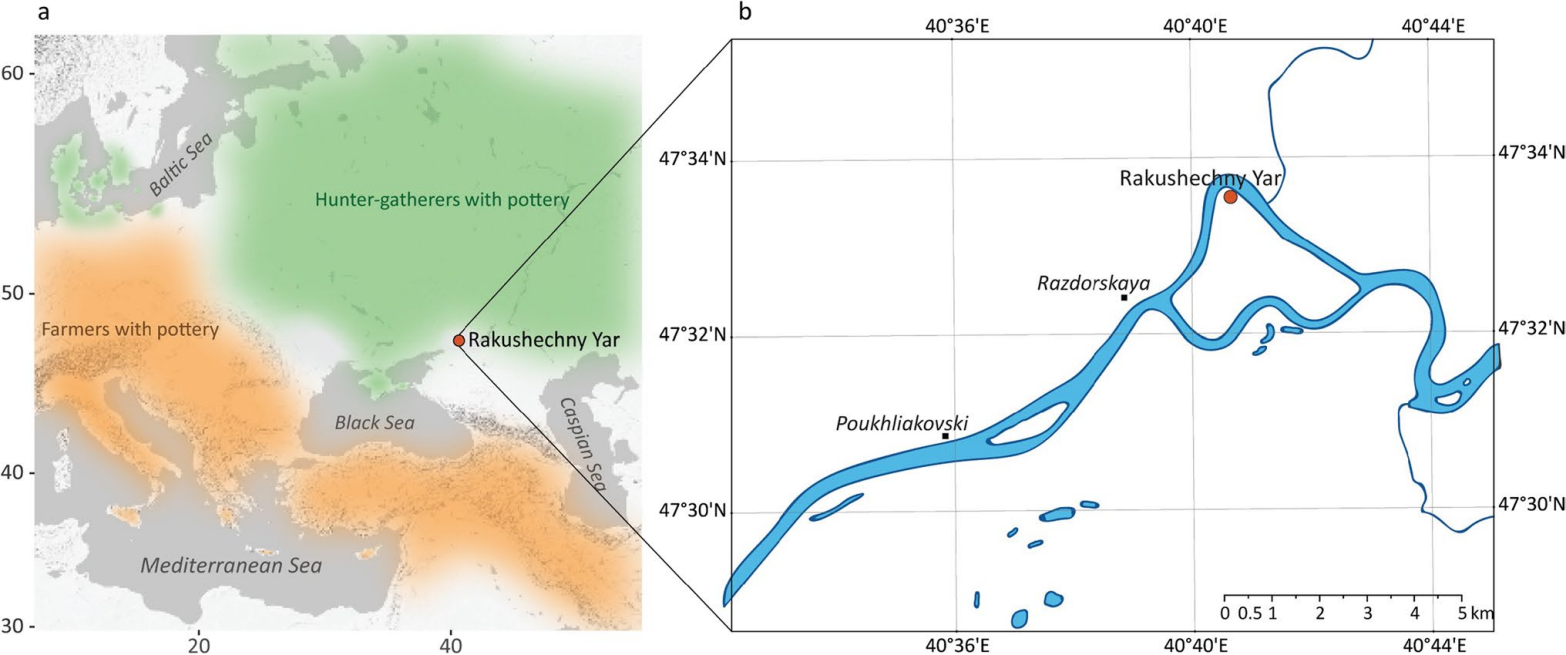
A simplified map (**a**) showing the location of Rakushechny Yar at the southern fringe of Eastern Europe (Russia), lying between ceramic foragers and farmers at *ca.* 5000 cal BC (**b**) showing the location of Rakushechny Yar on Porechny Island, in the lower Don River

In Europe, pottery was used by both hunter-gatherers in the north-east of the continent, during the early 6th millennium BC (Piezonka 2011; Kriiska et al. 2017) and by the first agricultural communities, such as those in the Balkans, at least by the end of the 7th millennium (Krauß et al. 2018; Fig. 1a). From the available dates and site locations, it is often assumed that these pottery traditions were associated with quite separate cultural trajectories with independent origins (Dolukhanov et al. 2009a, b; Davison et al. 2009; Fuller et al. 2015; Jordan et al. 2016). Eurasian hunter-gatherers and early farmers had different uses for pottery; aquatic products are largely absent from pottery produced by early farming communities, which are instead dominated by ruminant products including dairy products (Evershed et al. 2008b; Nieuwenhuyse et al. 2015; Debono Spiteri et al. 2016). These findings support the notion that ceramic using foragers and early farmers operated in two independent worlds (Gronenborn 2008).

Some of the earliest pottery vessels in Europe are found in the North Pontic-Caspian steppe during the early 6th millennium BC; a region lying between these worlds (Fig. 1a). Here, knowledge of ceramic production may have been acquired by hunter-gatherers from agriculturalists and pastoralists already established in Anatolia and the Caucasus (Fig. 1a) or brought directly by farmers who moved into this region and adjusted their practices by adopting a mixed foraging/farming economy. Alternatively, pottery production may have been introduced by other forager communities to the north and east or may even have been a quite separate local innovation. Unfortunately, dating the arrival of pottery throughout this region at the required precision to investigate these questions is currently hampered by the lack of detailed site-based chronological models. The problem is further exacerbated as individual dates of hunter-gatherer vessels are often made on adhering charred ‘foodcrusts’ which may be subject to significant freshwater or marine reservoir effects (Fischer and Heinemeier 2003; Philippsen 2013).

In order to examine the different aspects of pottery production in this region, here we present lipid residue analysis of vessels from one of the key sites, Rakushechny Yar, a hunter-gatherer site situated close to the putative forager/farmer contact zone in the Pontic steppe (Fig. 1a) that dates from the mid/late 6th millennium BC (Supplementary Materials). Residue analysis provides direct evidence of vessel use, from which we can infer possible functions and hence the motivations for the adoption of this technology by hunter-gatherers at regional and sub-regionals scales, as well as contributing to the emerging picture of regional variability in early pottery use in North-Eastern Europe (Courel et al. 2020). We were also interested in whether domesticated animal products were present, particularly on ceramic from the earliest layers at the site. If so, this evidence would support the hypothesis that pottery in this region was acquired through interaction with farmers and more broadly that ideas, materials and people flowed across the ‘boundary’ between foragers and farmers (Rowley-Conwy et al. 2014).

In support of the latter hypothesis, analysis of material culture found at Rakushechny Yar and more broadly in the North Pontic region suggests that the region was embedded in a wide cultural network that possibly encompassed farming communities to the south (Gorelik et al 2016). Neolithic obsidian from Southern Ukraine was obtained from Armenia and Central Anatolia (Biagi et al. 2014) and the presence of wattle-and-daub architecture at Rakushechny Yar (layer 11) and a female figurine (layer 10) (Belanovskaya 1995; Tsybrij et al. 2017) are similar to the many found in Anatolia (Cauvin 2000; Budja 2005, 2009). Similarly, Early Neolithic pottery at Rakushechny Yar is noted to share some technological and morphological attributes with SW Asian pottery produced by farmers (Vandiver 1987; Le Mière and Picon 1998; Nishiaki and Le Mière 2005; Budja 2009; Mazurkevich and Dolbunova 2015). Whilst there are also some shared technological and morphological traits between the Rakushechny Yar pottery and hunter-gatherers pottery from the neighbouring Lower Volga region, both the ceramic manufacturing techniques and the high proportion of undecorated pottery appear to have been quite different (Mazurkevich et al. 2017; Mazurkevich and Dolbunova 2012, 2015). Thus, the origin of the pottery at Rakushechny Yar based on stylistic and technological aspects is still unclear.

**Fig. 2:**
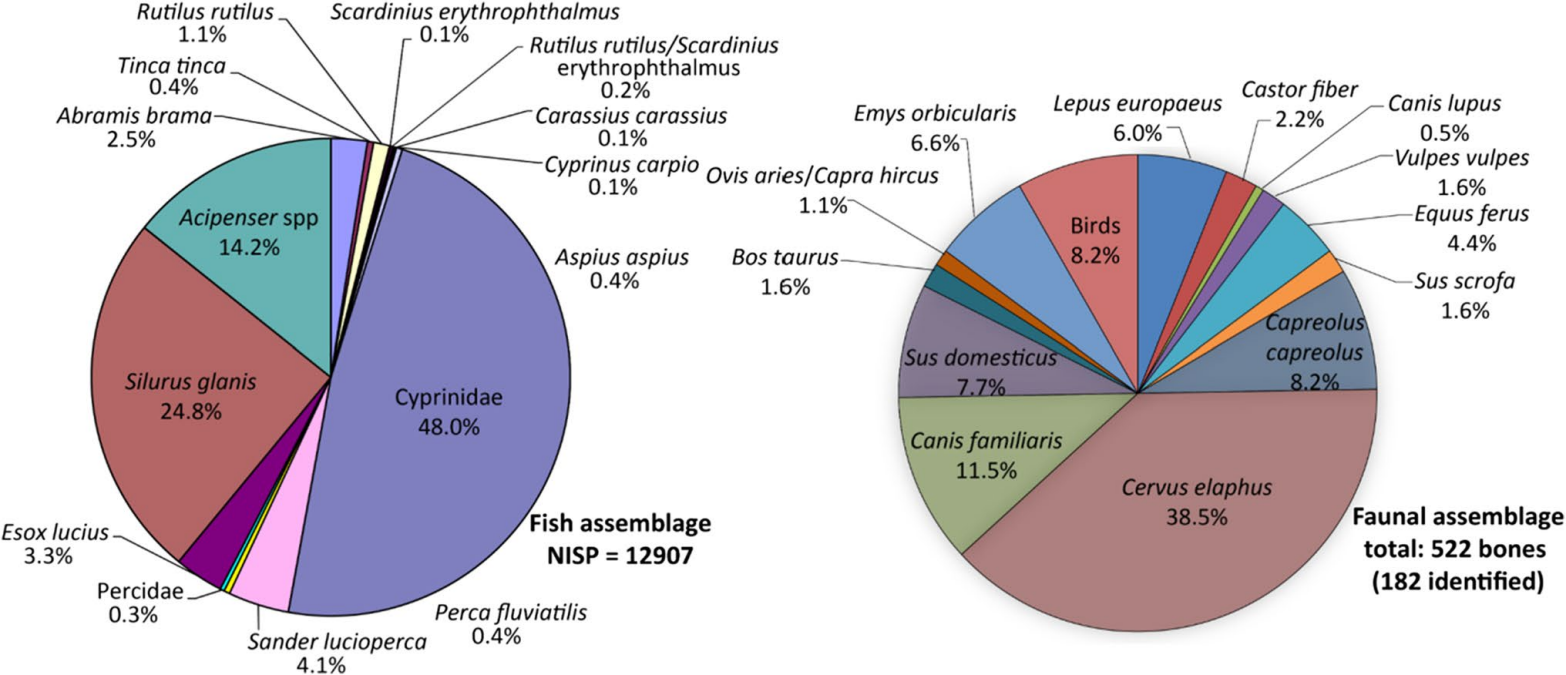
Archaeozoological assemblage distribution of fish and terrestrial animals from the Early Neolithic phase of Rakushechny Yar (Sablin 2018)

Although the faunal remains at Rakushechny Yar show that the site is clearly associated with hunting and fishing, it has also been reported that domesticated animals (cattle, *Bos taurus*, sheep, *Ovis aries*, goat, *Capra hircus*, pig, *Sus domesticus,* Fig. 2) were present in the faunal assemblage (from layer 21; (Belanovskaya 1995; Tsybrij et al. 2017). The presence of wild cattle and wild boar in this region precludes the secure identification of domesticated forms of these species on morphological criteria. In contrast, wild sheep and goats are thought to have been absent (Bobrinskoy et al. 1944), so the presence of these taxa would provide clear evidence of domestication. A second aim therefore was to use molecular identification (ZooMS) and AMS dating of mammalian bone to confirm or refute the presence of domesticated sheep/goat during the early phases of the site.

## Rakushechny Yar: site and context

Rakushechny Yar is located on the northwest part of Porechny Island (Fig. 1b). The site was established on the shoreline of an ancient naturally dammed lake (Dolbunova et al. 2020b). The first stage of occupation at Rakushechny Yar by Early Neolithic communities was initially dated to the 7th millennium BC (Tsybrij et al. 2017), but this has been more recently revised, based on new AMS dates, to the middle of the 6th millennium BC (Dolbunova et al. 2020b); Supplementary Materials). It was then occupied, possibly seasonally, until the Eneolithic and Bronze Age period dated to the 5th–4th millenium BC (Mazurkevich and Dolbunova 2012; Dolbunova 2016; Dolbunova et al. 2020b). Twenty-three cultural layers were identified, each of them clearly separated by sterile interlayers allowing fine chronological reconstruction. The cultural layers from 23 to 11 are attributed to the Early Neolithic phases.

Ceramic vessels recovered from the earliest cultural layers at Rakushechny Yar show variation in terms of form, volume and technology. Thirteen different vessel forms, attributed to the Early Neolithic layers, were identified constituting what we can consider a complete repertoire of ‘kitchenware’, encompassing plates, cooking pots, bowls (Fig. S1), all with different volumes, from tiny vessels (< 0.5 L) to much larger containers (15–20 L). Pottery making therefore seems to be highly developed from the outset, consistent with models for adoption rather than independent innovation. The ceramic vessels are generally with flat bottoms and are largely undecorated (91%). Nevertheless, pointed bottomed pottery was also found and some of the pottery was covered with red and/or yellow ochre and used as ochre containers (Mazurkevich and Dolbunova 2015). Four different *chaînes opératoires* were reconstructed as well as the use of various local clay sources and paste recipes reflecting the adaptation of several techniques for pottery production (Mazurkevich and Dolbunova 2012, 2015; Dolbunova 2016; Dolbunova et al. 2020a; Kulkova et al. 2018).

The well-preserved faunal assemblage reveals a subsistence economy mainly based on hunting and fishing. Wild mammals, such as red and roe deer (*Cervus elaphus*, *Capreolus capreolus*), and riverine resources including both freshwater (e.g. Cyprinidae, Wels catfish*, Silurus glanis*, etc.) and anadromous (e.g. sturgeon, *Acipenser* spp.) fish and shellfish (e.g. *Unio* spp. and *Viviparus diluvianus*) (Belanovskaya 1995; Zabilska-Kunek 2019), provided the majority of resources (Fig. 2). There is limited evidence of widespread plant exploitation, with the recovery of only one water caltrop (*Trapa natans L.*). Charcoal remains from the site are interpreted as waste from wood processing or smoking (Dolbunova et al. 2020a). The faunal assemblage consists predominantly of kitchen waste accumulated in cultural layers. Red deer were butchered on site, as testified by finds from edible and inedible parts of the body (phalanges, including hooves, antlers fragments and teeth). Nevertheless, the location of the site and the amount of fish bones and shell remains, as well as the very specific lithic and bone tool assemblage, emphasize the importance of fishing activities at Rakushechny Yar. As discussed above, putative finds of domesticated animals have been reported in the lower layers (e.g. sheep, cattle, pig; Sablin 2018; Dolbunova et al. 2020b), but these represent only ca. 11% of the mammalian assemblage (Fig. 2) otherwise dominated by wild cervids. Although the cattle and pig bones have been assigned on morphological grounds to be from domesticated stock, further DNA analysis is needed to confirm their origin and whether the common domesticated phenotypic traits are present (Verdugo et al. 2019; Frantz et al. 2019). It is important to note that domesticated cereal impressions on pottery vessels only appear in later Eneolithic layers (Motuzaite-Matuzeviciute 2012).

## Materials and methods

### Lipid residue analysis and bulk stable isotope analysis of pottery

Permissions were obtained to sample 120 samples (74 sherds and 46 foodcrusts) for organic residue analysis, representing 95 vessels typologically ascribed to the Early Neolithic phase. For 22 vessels permission was granted to only sample foodcrusts to minimise destruction to the vessel. The samples were obtained from early Neolithic layers N10–16, 19–21 and 23 from the older excavation made in 1966 (Belanovskaya 1995) and from the more recent excavation including Sect. 1 and on viviparus layers (1 to 3) in Sects. 2–3; (Table S1; see also description in Dolbunova et al. 2020a). Layers 17–15 (Sect. 1) are dated to between *ca*. 5600–5500 cal BC and the other Early Neolithic layers are most likely of similar date, or even slightly more recent (Dolbunova et al. 2020b). In addition, lipids of modern edible endemic fruits and plants from Rakushechny Yar’s vicinity (e.g. bulrush, *Typha*; wild thyme, *Thymus*; silverberry, *Elaeagnus*; wild rose berry, *Rosa*; wild pear, *Pyrus*; wild apple; *Malus*), modern shellfish (*Viviparus*, *Unio*) from the Lower Don River, modern fish (freshwater or migratory species) and wild ruminants (e.g. reindeer, *Rangifer tarandus;* elk; *Alces alces*, roe deer) from Russia were analysed (Table S2) for comparison with the lipids derived from archaeological ceramic samples. Lipids from soil from some of the cultural layers of the site were also extracted to control any potential environmental contamination (Table S1).

Lipids extracts were obtained for all the samples (potsherds: ca. 1 g; foodcrusts ca. 20 mg) using a modified one-step acidified methanol methodology (Craig et al. 2013). When sufficient material was present, an additional solvent extraction following published protocols (Bondetti et al. 2020b) was performed (*n* = 20), to enable the detection of any triacylglycerols or wax esters. Lipid extracts were analysed using gas chromatography-mass spectrometry (GC–MS) with different columns and modes. For the methanolic acid extracts yielding a sufficient amount of lipids, the carbon isotope values of palmitic acid (C_16:0_) and stearic acid (C_18:0_) were obtained using gas chromatography-combustion-isotope ratio mass spectrometry (GC-C-IRMS). Elemental analysis-isotope ratio mass spectrometry analysis (EA-IRMS) was also performed on charred foodcrusts samples (∼ 1 mg) for the determination of their stable nitrogen (δ^15^N) and carbon (δ^13^C) isotope values, as detailed in previous publications (Craig et al. 2007; Lucquin et al. 2016b). Further information on the extraction methodologies employed here and the instrument conditions are provided in the Supplementary Materials.

### Digital microscopy and scanning electron microscopy (SEM)

Where the quantity of charred foodcrusts was sufficient, digital and scanning electron microscopy was conducted to complement the lipid analysis. This approach has previously been tested and proved successful in determining the presence of plant products in foodcrusts adhered to prehistoric pottery from the Netherlands (Raemaekers et al. 2013). Seven of the 50 charred foodcrusts from Rakushechny Yar were selected for microscopic analysis (samples 915, 916, 920, 921, 926, 927 and 929); these were chosen from the samples available due to their good preservation and thicker macrostructure which provided a larger mass of material and allowed a thorough investigation of their composition. Initial observation under low-powered microscopy was carried out using a Leica MZ APO binocular microscope at magnifications of between 8 and 50x. In order to study the microstructure and physical attributes of the residues, images were created using a VHX-5000 Keyence digital microscope at magnifications from 20 to 200x. From these, food fragments that presented visible inclusions (such as putative animal or plant tissues) were selected for further study under SEM. For SEM observation, samples were cleaned from adhered materials such as clay residue and sediments with a brush and sputter coated with *ca*. 1 micron of gold when necessary for image quality purposes. These were then examined using a Hitachi S-3700 N scanning electron microscope housed at the Scientific Research Department of the British Museum. The two main aims of these analyses were to identify specific types of particles visible in the foodcrusts, such as plant and animal tissues, in order to elucidate the ingredients used, and the exploration of the foodcrusts’ microstructures in order to distinguish potential processing and cooking techniques which might have led to the accumulation of these residues.

Selected fragments of foodcrusts were analysed using SEM for specific observation of the matrix/microstructure. During this, up to five images of the matrix of each fragment were captured, at 10–16 mm working distance and at 50 × magnification, to cover the whole surface. Then, the visible particles were captured one by one for further identification. Identification of any tissues was carried out in comparison with modern and archaeological reference materials as well as available published data.

### Bulk analysis of bone collagen

Stable isotopic analysis of bone collagen was undertaken from a range of fauna from the site to provide a broad reference with which to interpret the lipid data. Archaeological bones of deer (*n* = 24), pig (*n* = 7), beaver (*Castor fiber*, *n* = 1), horse (*Equus ferus*, *n* = 3), zander (*n* = 4) and Wels catfish (*n* = 1) from Rakushechny Yar were selected (Table S3) to serve as an archaeological isotopic reference. Bone collagen was extracted using a standard procedure developed by Longin (1971) and Brown et al. (1988) and then modified in Alexander et al. (2015). Samples were subsequently isotopically analysed by EA-IRMS akin to the foodcrust samples. Further information about the methodology is provided in the Supplementary Material.

### ZooMS (zooarchaeology by mass spectrometry)

To confirm the presence of domesticated species, 18 bone samples from different layers at Rakushechny Yar were subjected to collagen peptide mass fingerprinting, also known as ZooMS. Four of the bones had been morphologically identified as sheep or goat, while the others could only be assigned to the family Bovidae or unidentified (Table S5). Frequently used in archaeology and palaeontology, ZooMS was developed by (Buckley et al. 2009) as a technique to identify collagen-based remains (i.e. bone, antler, leather) and is particularly useful for fragmented or worked remains where morphological indicators have been modified or removed. It involves peptide mass fingerprinting of Type I collagen using matrix-assisted laser desorption/ionisation time-of-flight mass spectrometry (MALDI-ToF–MS). Extracted collagen peptides from a specimen are compared with known peptide markers in order to identify the source of the raw material, usually to the genus level. ZooMS was performed following a slightly modified method to that outlined in (Buckley et al. 2009), with the extracted collagen peptides analyzed on a Bruker ultraflex III MALDI-ToF–MS. Sample spectra were analysed using mMass software (www.mmass.org; Strohalm et al. 2008) and compared with a database of known *m/z* markers (Buckley et al. 2009, 2010; Buckley and Collins 2011; Kirby et al. 2013). More information about the protocol and instrument conditions are available in the Supplementary Material.

### *AMS *^*14*^*C dating*

The ceramic assemblage discussed in this paper can be dated by stratigraphic association with mammal bones interpreted as food waste, which have been dated by accelerator mass spectrometry (AMS) following routine protocols. Bone identifications have been tested by ZooMS, as described above, and collagen from the dated bones analysed by EA-IRMS, providing additional isotopic reference data. A summary of relevant methods and results, and a re-evaluation of legacy ^14^C data from Rakushechny Yar, is provided in the Supplementary Material.

## Results and discussion

### Preservation of animal fats in Rakushechny Yar pottery

The preservation of lipids in association with pottery from Rakushechny Yar was excellent, with 94% of the samples yielding amounts of lipids above the accepted threshold of interpretation (i.e. > 5 μg/g for potsherd or > 100 μg/g for foodcrusts) (Evershed et al. 2008a; Craig et al. 2013). Overall, the lipid profiles are characterized by a complex mixture of aliphatic compounds, encompassing saturated fatty acids ranging from C_6:0_ to C_30:0_, monounsaturated fatty acids (C_14_-C_24_), branched fatty acids (C_12_-C_19_) and dicarboxylic acids (C_5_-C_15_) (Fig. S2a). Cholesterol and its derivatives were also detected in over one third of the samples. Overall, the lipid evidence points overwhelmingly to the presence of animal fats in the majority of pots from Rakushechny Yar, which could include both aquatic and terrestrial animal sources.

Only one sample (151) displayed a different molecular profile (Fig. S2b), with diterpenes typical of Pinaceae resin and wood (dehydroabietic acid, methyl-dehydroabietic acid and 7-oxo-dehydroabietic acid) along with traces of retene characteristic of thermal treatment (Colombini et al. 2005; Modugno and Ribechini 2009). The absence of methyl-dehydroabietic acid in the solvent-extracted extracts seems to support the use of resin rather than the processing of resinous pine wood (Modugno and Ribechini 2009). Interestingly, this vessel had a very specific elongated form (Fig. S3), similar to ‘oil lamps’ recovered from hunter-gatherer Baltic contexts (Heron et al. 2013). Whilst these compounds can arise from environmental contamination (Naihuang et al. 1995; Costa et al. 2016), the absence of such markers in the sediments collected at Rakushechny Yar does not favour this interpretation. Interestingly, anthracological analyses of the charcoals recovered from the site revealed the non-use of pine as firewood, favouring instead other wood species (e.g. *Salix* sp. willow and/or *Populus* sp. poplar) (Dolbunova et al. 2020a). The detection of Pinaceae markers in this pottery might suggest particular usages of pine wood. Pinaceae resin may have had various functions in prehistory, as an adhesive, a disinfectant or as a waterproofing agent (Regert 2004; Mitkidou et al. 2008; Rageot 2015; Rageot et al. 2021). However, from its form, the vessel could have been used for burning conifer resin for its odiferous properties, i.e. as an incense (Lucquin et al. 2007) or conceivably as an insect repellent. Interestingly, no aquatic-derived lipids were detected in this sample which contrasts with the western Baltic examples that were mainly used for the burning of aquatic oils (Heron et al. 2013).

### Molecular and isotopic evidence for aquatic resources in pottery

**Fig. 3:**
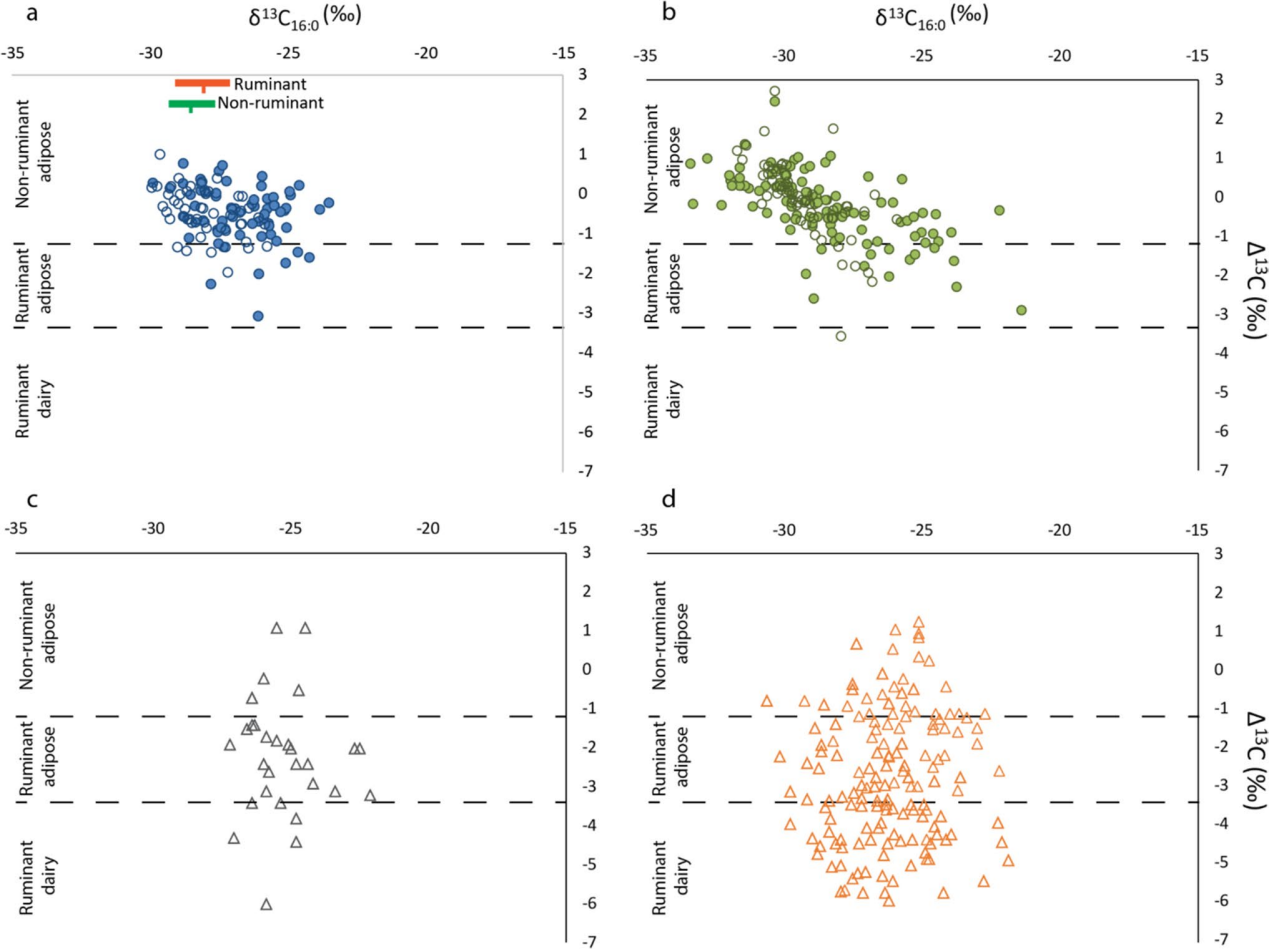
Plot of Δ^13^C values against δ^13^C_16:0_ values of (**a**) Rakushechny Yar samples compared with pottery from (**b**) Zamostje 2 site (Bondetti et al. 2020b) and early agricultural sites in (**c**) Syria (Nieuwenhuyse et al. 2015) and (**d**) Anatolia (Evershed et al. 2008b; Debono Spiteri et al. 2016). For (**a**) and (**b**) plots, the filled circles indicate samples with aquatic signals. For (**c**) and (**d**) aquatic derived lipids are not reported but presumed to be absent. The data generated here are compared with the mean and ranges (2σ) of expected lipid δ^13^C values, based on bone collagen of archaeological non-ruminant (including pig and beaver) and ruminant (red deer) recovered at Rakushechny Yar, plotted on the x-axis only. The collagen δ^13^C values were adjusted by − 8‰ to correct for the collagen to tissue offset in order to make these values comparable with δ^13^C_16:0_ of lipids extracted from pottery (Fernandes et al. 2015)

The stable carbon isotope values of the two most abundant unsaturated fatty acids (C_16:0_ and C_18:0_) were measured for 112 samples in order to provide further information regarding contents. The Δ^13^C (δ^13^C_18:0_–δ^13^C_16:0_) value avoids environmental influences on the absolute δ^13^C values (Copley et al., 2005) and has been widely used to discriminate ruminant adipose and dairy fats from other non-ruminant sources (e.g. Craig et al. 2012, 2013; Cramp et al. 2014; Taché and Craig 2015; Colonese et al. 2015; Lucquin et al. 2016b). These values are plotted against δ^13^C_16:0_ in Fig. 3a. At Rakushechny Yar, over 85% of the samples have non-ruminant lipid isotope signatures. Considering the faunal assemblage at Rakushechny Yar, non-ruminant sources could include aquatic or non-ruminant terrestrial animals, such as pig, beaver or horse.

A total of 64 samples, corresponding to 56% of the vessels analysed, contained diagnostic compounds for aquatic foods, either including *ω*-(*o*-alkylphenyl)alkanoic acids (APAAs) containing 20 or more carbon atoms alongside at least one isoprenoid acid (either 4,8,12-trimethyltridecanoic acid, phytanic or pristanic acid (Evershed 2008; Hansel and Evershed 2009; Cramp and Evershed 2014), or a contribution of SRR-isomer of phytanic acid > 75%. APAAs arises from the heating (at least 1 h of heating at a temperature ≥ 200 °C; (Bondetti et al. 2020c) of their precursor long-chain unsaturated fatty acids, which occur only in an appreciable amount in freshwater and marine animals (Hansel et al. 2004; Evershed et al. 2008a) and constitute, therefore, an important marker for the cooking of aquatic source fats in pots.

Whilst APAA-C_20_ are also produced by heating terrestrial mammals and plants, the relative abundance of these compounds is much greater in processed aquatic products (Bondetti et al. 2020c). The calculation of the relative abundance of APAA-C_20_ vs. C_18_ provides a new criterion for identifying whether these compounds arose from the processing of aquatic or terrestrial commodities. Rakushechny Yar samples overall display a high APAA C_20_/C_18_ ratio (x̄ = 0.23 ± 0.10) consistent with values from authentic fish (> 0.06). Likewise, although phytanic acid occurs both in the tissues of aquatic and ruminant animals, the ratio of the phytanic diastereomers (3S,7R,11R,15-phytanic acid [SRR], and 3R,7R,11R,15-phytanic acid [RRR]) provides an additional tool to distinguish these sources (Lucquin et al. 2016a). A greater contribution of SRR-isomers (i.e. > 75%) is usually ascribed to aquatic species (Lucquin et al. 2016a). A further 20 samples contained C_18_ APAAs and at least one isoprenoid or an SRR% between 65.6 and 75.5% (Table S1), indicating that aquatic resources were most likely processed in these vessels as well (Evershed et al. 2008a; Heron et al. 2015; Lucquin et al. 2016b), although definitive evidence is lacking.

The other samples falling in the non-ruminant isotopic value area, without aquatic biomarkers, might either result from the degradation of these specific molecules or could also reflect the processing of non-ruminant terrestrial products rather than aquatic resources. These samples tend to have fatty acids more depleted in ^13^C (δ^13^C_16:0_ = -28.3 ± 0.9‰; δ^13^C_18:0_ = -28.6 ± 0.7‰) than samples with aquatic biomarkers (δ^13^C_16:0_ = -26.7 ± 1.4‰; δ^13^C_18:0_ = -26.9 ± 1.4‰; Mann–Whitney test: U = 193; z = 4.3; *p* < 0.01 for δ^13^C_16:0_ and U = 144; z = 4.9; *p* < 0.01 for δ^13^C_18:0_) in keeping with measurements made on collagen from beaver, horses and pigs from the site (Fig. 3a, Table S3) after a − 8‰ collagen to lipid offset has been applied (Fernandes et al. 2015). Nevertheless, in the absence of further diagnostic lipids, a more precise identification of non-ruminant terrestrial sources cannot be clearly established.

**Fig. 4:**
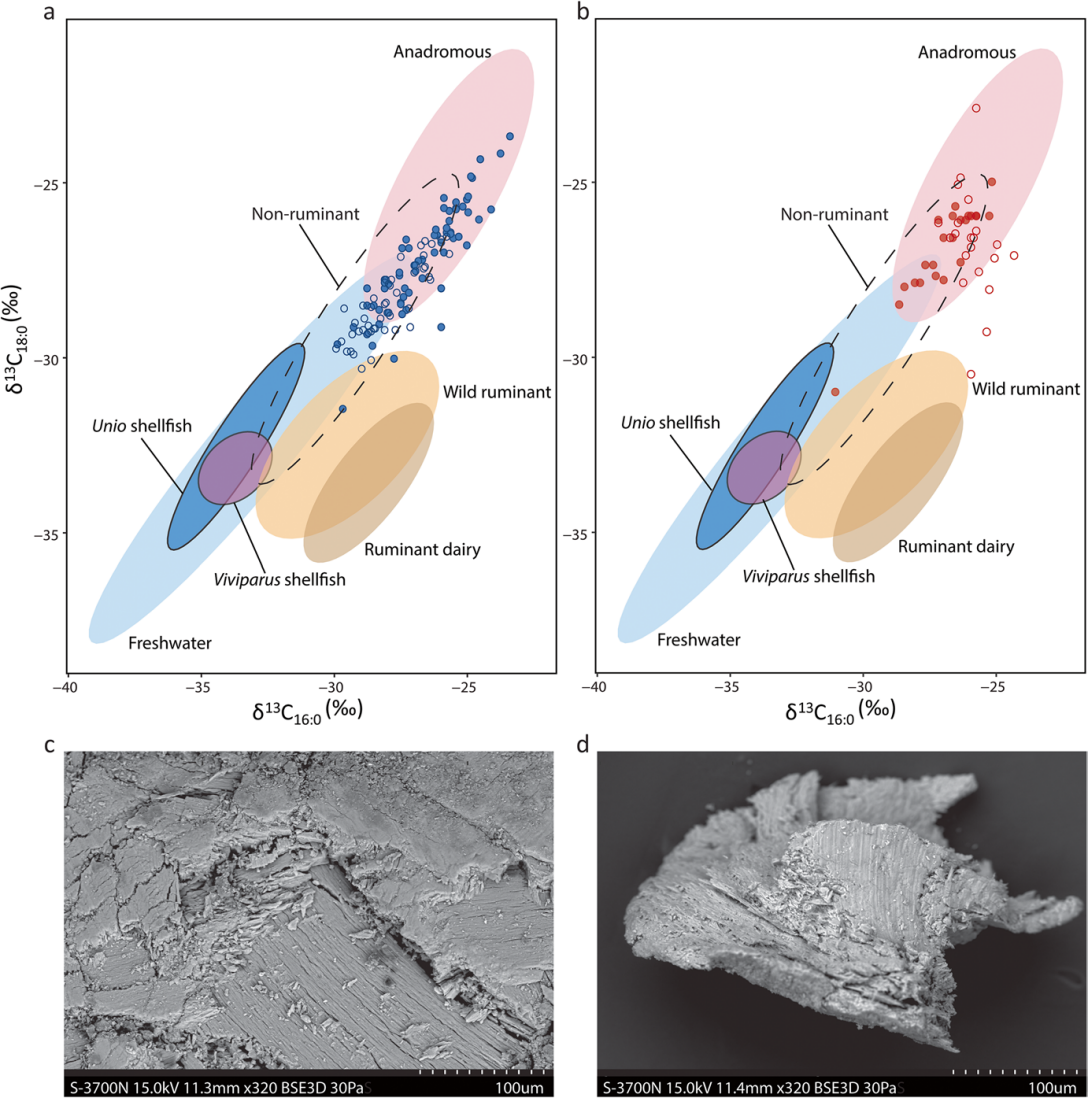
Plot of the δ^13^C values of C_16:0_ and C_18:0_ *n*-alkanoic acids extracted from (**a**) Rakushechny Yar pottery from Early Neolithic layers and (**b**) Iron Gates region ceramic vessels (Cramp et al. 2019). SEM micrographs showing fragments of sturgeon bony components (**c**) in a foodcrust sample from Rakushecnhy Yar (Raku-929) and (**d**) of archaeological sturgeon reference materials. Samples with aquatic biomarkers are shown by filled circles. The data are compared with reference ranges for authentic reference lipids from modern tissues from published studies (Dudd 1999; Spangenberg et al. 2006; Outram et al. 2009; Craig et al. 2011, 2012, 2013; Taché and Craig 2015; Pääkkönen et al. 2016, 2020; Lucquin et al. 2016b; Choy et al. 2016; Courel et al. 2020) and additional new *Unio* and *Viviparus* shellfish data from the Low Don River and freshwater fish and ruminants from Russia (95% confidence; Table S2). To allow comparison with the archaeological samples from the Holocene period, the δ^13^C values of the modern samples were adjusted given the variation in the atmospheric δ^13^C resulting from post-industrial carbon according to the known or estimated year of death of the animal (Hellevang and Aagaard 2015)

The δ^13^C_16:0_ and δ^13^C_18:0_ values of Rakushechny Yar samples are plotted in Fig. 4a. These have a wide range of δ^13^C isotopic values varying between − 23.5 and − 30.3‰. Whilst some samples have fatty acid δ^13^C values corresponding to the reported range for modern authentic freshwater fish oils (Fig. 4a), the majority have more positive δ^13^C values outside this range. These are more consistent with values expected from anadromous or marine fish (Craig et al. 2011, 2013; Pääkkönen et al. 2016; Lucquin et al. 2016b; Choy et al. 2016). Given the site’s location, some distance from the Sea of Azov and Black Sea (*ca*. 100 and 500 km from Rakushechny Yar site, respectively), the residues in the pottery are most likely attributed to anadromous fish, such as sturgeon, than marine species. Anadromous fish spend most of their lives in the sea and ocean and migrate into riverine habitats to spawn (Zydlewski and Wilkie 2012). Therefore adult anadromous fish, having fed mainly in the marine environment, maintains a relatively enriched δ^13^C signature even when caught up-river (McCarthy and Waldron 2000). Sturgeons are the third most represented species in the fishbone assemblage (Zabilska-Kunek 2019). Conversely, pots with relatively depleted ^13^C are more in keeping with freshwater fish such as cyprinids *(*Cyprinidae) and Wels catfish (*Silurus glanis*), constituting the major part of the fish remains in Rakushechny Yar (Zabilska-Kunek 2019), or even juvenile sturgeon which would still provide a more freshwater signal (Doucett et al. 1999).

To test these assumptions, we examined δ^13^C collagen values of fish present in the assemblages and reported in the literature. A correction of − 7‰ was applied to the collagen values to allow comparison with lipid values (Fernandes et al. 2015). The mean adjusted carbon isotopic value obtained from non-migratory freshwater fish recovered at Rakushechny Yar (catfish and zander) is − 28.5‰ (± 3‰; *n* = 5; Table S3). While this is consistent with the more depleted range of lipid δ^13^C values obtained from pottery at Rakushechny Yar, one zander had a much more positive δ^13^C value (Table S3). There was insufficient collagen in the sturgeon bones from Rakushechny Yar for analysis, but collagen isotope data from sturgeon bones from the Danube Gorges or Central Balkan sites show generally more positive δ^13^C values after applying the lipid to collagen correction (x̄ =  − 26.6 ± 1.2‰; *n* = 4; Borić et al. 2004; Jovanović et al. 2019) than freshwater species, although their ranges overlap.

A small proportion of samples contained aquatic lipids that could also be derived from the beaver fats (Fig. 4a, Table S2) based on their fatty acid δ^13^C values. Beavers were exploited at Rakushechny Yar but only form a minor part of the faunal assemblage (Fig. 2). As with aquatic organisms, beaver fats are characterised by high amounts of phytanic acid with a very significant contribution of the SRR-isomer (x̄ = 98.5 ± 2.9%) (Table S4) and APAAs C_20_ are produced when subjected to thermal treatment (Bondetti et al. 2020c). Nevertheless, the relatively high APAA C_20_/C_18_ ratio obtained from the Rakushechny Yar pottery precludes beavers, which exhibit a much lower value (< 0.06) (Bondetti et al. 2020c) and are instead consistent with fish or aquatic molluscs.

The homogeneity of the residues at Rakushechny Yar is intriguing, given the typologically diverse pottery assemblage analysed, which ranges from very small vessels (< 0.5 L) to large containers (15–20 L) with a wide range of thicknesses (Table S1). There was no statistical difference between the frequency of aquatic biomarkers when the data are disaggregated according to different volume categories, and there was no correlation with vessel wall thickness. When paired samples (charred foodcrusts and associated ceramics) are considered, the presence/absence of aquatic biomarkers are in accordance for 74% of the cases (*n* = 23). The isotopic values between charred foodcrusts and their associated ceramics are moderately correlated (Pearson: R = 0.67; *p* < 0.01 for δ^13^C_16_ and R = 0.53; *p* < 0.01 for δ^13^C_18_), with no systematic offset (δ^13^C_FA_ foodcrusts—δ^13^C_FA_ sherd; δ^13^C_16_offset =  − 0.36 ± 1.29, s^2^ = 1.67 (1σ) and δ^13^C_18_offset =  − 0.56 ± 1.56 (1σ), s^2^ = 2.44). Most likely, the ceramic matrix sample represents an accumulated lipid signal derived from multiple uses of the vessel where the foodcrusts represents more episodic cooking events, as suggested by long-term cooking experiments (Miller et al. 2020). Therefore, here we consider the ceramics matrix and foodcrusts as individual ‘samples’ representative of different ‘cooking events’. Whilst slight differences in the individual cooking events can be noted, the commodities processed appear to be similar overall (mean δ^13^C_16_ =  − 27.11 ± 1.46‰ (1σ) for foodcrusts and mean δ^13^C_16_ =  − 27.47 ± 1.66‰ (1σ) for ceramic; mean δ^13^C_18_ =  − 27.46 ± 1.64‰ (1σ) for foodcrusts and mean δ^13^C_18_ =  − 28.02 ± 1.57‰ (1σ) for ceramic; T-test: t = 0.78, *p* = 0.44 for δ^13^C_16_ and t = 1.20, *p* = 0.24 for δ^13^C_18_) supporting our inference of specialist ‘aquatic’ use of the pottery over its entire use-life.

**Fig. 5:**
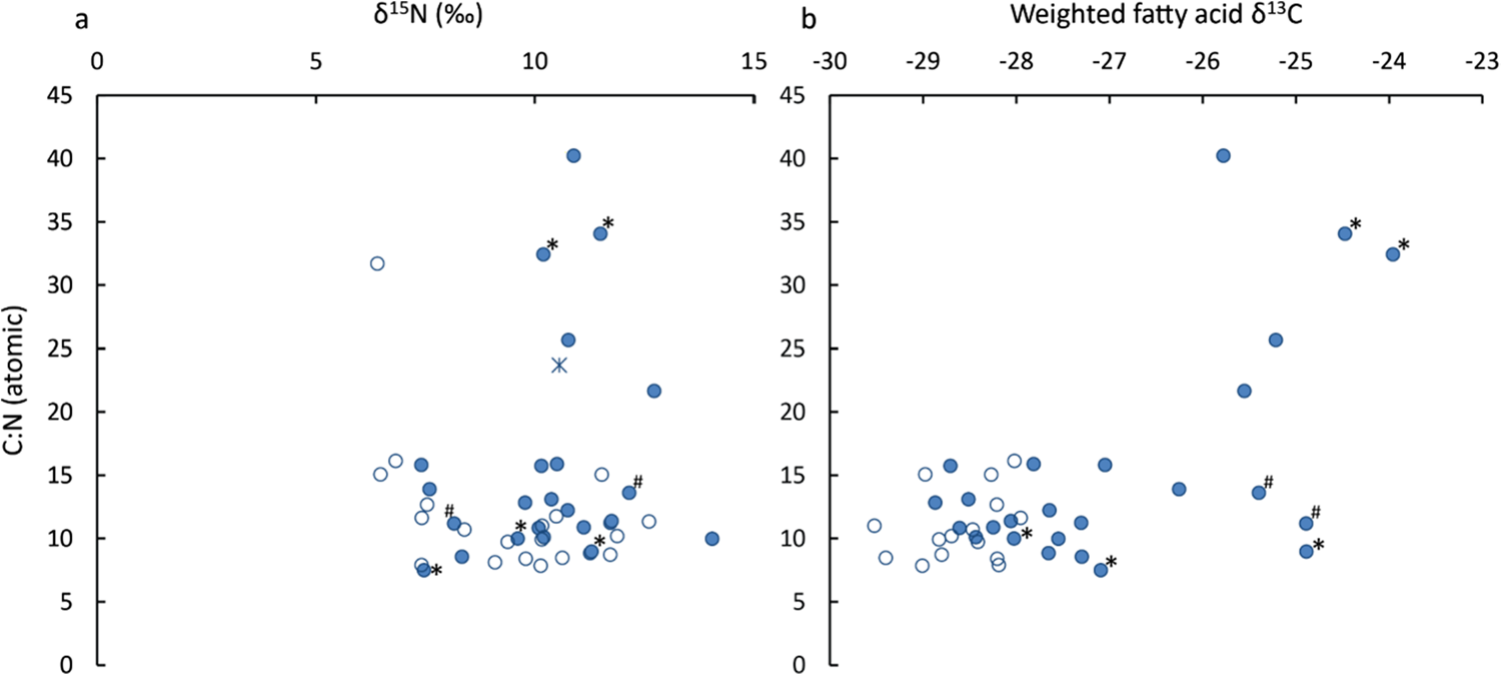
Plot of (**a**) δ^15^N against C/N ratio and (**b**) weighted fatty acid δ^13^C against C/N ratio obtained from Rakushechny Yar foodcrust samples of which fatty acids were available. Filled circles indicate samples with aquatic biomarkers, * represents samples where sturgeon osseous structures were detected by SEM, and ^#^ represents samples where cyprinid and sturgeon osseous structures were observed. The weighted fatty acid δ^13^C corresponds to the weighted average carbon isotope value based on the relative proportion of C_16:0_ and C_18:0_ fatty acids, as reflected in the P/S ratio ([δ^13^C_16:0_ × P/S + δ^13^C_18:0_]/ [P/S + 1])

Additionally, the bulk stable nitrogen (δ^15^N) and carbon (δ^13^C) isotope values of foodcrusts adhering to the ceramic vessels were measured. Figure 5a shows the δ^15^N values of foodcrusts plotted against the C/N ratios. Six samples have relatively higher C:N ratios (i.e. > 20) along with δ^15^N values above + 10‰, which is consistent with fish oil (Dufour et al. 1999; Heron et al. 2013; Craig et al. 2013; Choy et al. 2016). Interestingly, these samples and the pottery sherds they were associated with, have relatively enriched δ^13^C and previously ascribed to anadromous fish fats (δ^13^C_16:0_ =  − 25.7 ± 0.9‰ and δ^13^C_18:0_ =  − 26.1 ± 1.1‰) with over 85% of them having aquatic biomarkers (Fig. 5b). In addition, many of the foodcrusts exhibiting anadromous signature had a microscopically observable thin and oily microstructure also consistent with high-temperature processing and oil extraction.

The rendering of fish to produce storable oils would have helped to deal with the seasonal surplus of migratory sturgeon, available only during the late spring (Kovalchuk et al. 2018). Sturgeon, which can reach several hundred kilograms, likely constituted an important and valued source of food for prehistoric communities. Indeed, their flesh and roe are rich in fat and protein (Badiani et al. 1996; Ovissipour and Rasco 2011). Rendered oils could be stored and consumed during the colder seasons when resources were more scarce, but could also be accumulated and exchanged, in order to buffer risk but leading to the creation of ownership, debts and inequalities and social hierarchies (Hayden 2009). Other traditional non-culinary uses of fish products include collagen extraction for making glue and tanning of skins to make leather, all of which would have been valuable commodities (Jackinsky-Sethi 2014). The near absence of use-wear traces on the vessels suggests that they were not used for daily household activities but most likely for specialist activity during short-lived seasonal periods, although comparative experimental material would be needed to test this hypothesis.

The faunal remains at Rakushechny Yar also point to an extensive accumulation of shellfish, namely, *Viviparus diluvianus* and *Unio* (Dolbunova et al. 2020b). Although the natural or anthropogenic nature of *Viviparus* shells presence is unclear due to the current lack of research on them, several evidences indicate that the *Unio* shells have been widely used for various technological purposes (e.g. for the construction of platforms, as scraping tools and ochre containers), but they were also consumed potentially in large quantities. Given that an efficient way to extract mollusc meat from their shell is by boiling (Miracle 2002; Milner 2009), it is worth considering whether ceramic containers were used to facilitate this task, as has been suggested for Jōmon pottery in Japan (Ikawa-Smith 1976). However, the isotope values obtained from modern shellfish (*Unio* and *Viviparus diluvianus*) harvested in the Don River nearby Rakushechny Yar and corrected for the Suess effect (Hellevang and Aagaard 2015) rule out this hypothesis as their fatty acids are more depleted in ^13^C compared with those from the vessels (Fig. 4a). Instead, alternative methods may have been used for processing prior to consumption. In this sense, the discovery of a pit filled with burnt shell remains at Rakushechny Yar might suggest it was used as an oven for this purpose (Aldeias et al. 2019; Dolbunova et al. 2020b).

Similarly, ruminant animals make a significant contribution to the mammalian faunal assemblage but less than 15% of the ceramic samples have fatty acids with a ruminant stable carbon isotope signature (Δ^13^C values <  − 1; Fig. 3a). Over half of these samples contain aquatic biomarkers. A likely explanation is that ruminant carcass fats and fish oils were mixed in these pots. Bondetti et al. (2020a) and others (Cramp et al. 2019) have shown that negative Δ^13^C values are theoretically observed when fish oils are mixed with even a modest (ca. > 10%) amount of ruminant fats due to differences in the fatty acid concentrations between these products. Furthermore, some of these samples exhibit more enriched δ^13^C values than archaeological and modern wild-ruminants from Rakushechny Yar and Western Russia, respectively, also indicating that they were mixed with anadromous fish (Fig. 3a; Table S3). Overall, therefore ruminant carcass fats seem to be only a minor addition to culinary practices involving cooking pots at Rakushechny Yar.

### Digital microscopy and scanning electron microscopy

Analysis of foodcrusts by scanning electron microscopy (SEM) showed thin and compacted microstructure formed by vitrified shiny layers of charred matter. Interestingly, the appearance of the microstructure contrasts with previous analysis of charred material interpreted to be derived from boiling plant foods (Gonzalez Carretero et al. 2017; Gonzalez Carretero 2020), which tend to be more porous, suggesting a different mode of use as noted above. Particles with variable degrees of charring were clearly visible embedded in this microstructure. All seven of the analysed foodcrusts were seen to contain small fragments of sturgeon bony structures. In most cases, these were found to be derived from dermal scutes as they had a laminated microstructure typical of this type of bony tissue. In addition, potential fragments of sturgeon fin spine bones or round based scales have been identified in a single foodcrust, from sample 926. Fragments of sturgeon bones appeared embedded in the foodcrusts’ microstructures and their preservation differed from completely charred to non-charred. These were seen to measure between *ca*. 50 µm and 1000 µm in size and presented a clear laminated microstructure formed by a grid of parallel tubes forming horizontal bony layers as seen from modern and archaeological sturgeon reference materials (Hilton et al. 2011; Thieren et al. 2015; Figs. 4c and d). Positive identification of these remains as fragments of sturgeon bone structures was possible through the comparison with reference sturgeon bones, in particular lateral scutes and fin fragments. Reference materials were provided by Museum of London Archaeology (MOLA).

In addition to the remains of sturgeon bony structures, foodcrusts from samples 915 and 921 were seen to contain remains of partially preserved soft-edged (cycloid) fish scales. Based on the observation of typical morphological traits (Esmaeili et al. 2007), these are believed to belong to members of the cyprinid family (e.g. common bream, *Abramis brama*, carp, *Cyprinus carpio*, asp, *Aspius aspius* or tench, *Tinca tinca*) widely present in the archaeological fish bone assemblage at the site (Zabilska-Kunek 2019). In contrast to cyprinids, the other types of fish recovered from the archaeological record at Rakucheshny Yar, such as Wels catfish and sturgeon, have scale-less bodies. In the case of Wels catfish, their bodies are covered in a slime-like substance which is invisible in the archaeological record.

In contrast with the high proportion of fish, microscopic analysis of the selected samples has shown no clear evidence of any other animal products being used. Potential plant tissues were identified in three of the seven foodcrusts analysed. Although not well enough preserved to allow identification to type or genus level, these have similar appearances to some of the tissues contained in the epidermis of grasses, such as the cells of the aleurone layer containing what looks like aleurone protein. This is specifically seen in samples 920, 926 and 927.

### Were domesticated animals introduced with pottery at Rakushechny Yar?

No dairy fats were detected in any of the pottery vessels from Rakushechny Yar (Fig. 3a and 4a). All the Δ^13^C values are above the range for modern dairy reference fats (-3.3‰; Fig. 3a). Other clear molecular indicators of dairy, such as lower molecular weight triacylglycerols, were also absent. Twenty-five samples exhibited short-chain fatty acids (C_6:0_ to C_12:0_) (Table S1) characteristic of milk fats (Christie 1989; Dudd et al. 1998; Copley et al. 2003), although these most likely reflect the degradation of longer free fatty acids through thermal or catalytic cracking, or by bacterial action (Shimoyama et al. 1993; Raven et al. 1997). The absence of dairy contrasts sharply with the results of residue analysis of pottery from agricultural Early Neolithic sites in southwest Asia, which contained a high proportion of ruminant dairy and fat products (Evershed et al. 2008b; Nieuwenhuyse et al. 2015; Debono Spiteri et al. 2016; Fig. 3c and d).

Although the obtained results cannot rule out the presence of domesticated ruminant carcass fats in the pottery, these were likely to have been a minor contribution and are most likely derived from wild ruminants, in particular cervids, that dominate the mammalian bone assemblage (Belanovskaya 1995; Dolbunova et al. 2020b). The pattern of pottery use at Rakushechny Yar instead resembles other hunter-gatherer examples, such as Zamostje 2 in the Upper Volga (Fig. 3b) and the Estonian Narva culture (Oras et al. 2017), focused on aquatic resources, notably fish.

Given the absence of any clear evidence for domesticated animal products in the pottery from Rakushechny Yar, it was important to establish whether any domesticated species were present in the Early Neolithic faunal assemblage. Of the 18 samples of bones subjected to ZooMS analysis, only three could be assigned to sheep or goat (Table S5). One of these, recovered from upper layers (upper vivip layer 1) failed to produce an AMS date, but is derived from layers with other bones that were directly dated to the 3rd millennium BC, so could not be assigned to the Early Neolithic. A goat tooth, from an unclear context, was dated to 80–250 cal AD (SUERC-94517, 1855 ± 31 BP) (Table S6) and is the most recent dated remains so far at Rakushechny Yar. Finally, a bone identified as sheep/goat from the vivip 2 layer was dated to the Late Neolithic/Eneolithic period (SUERC-94518, 5433 ± 31 BP, 4350–4240 cal BC). Conversely, two samples from the Early Neolithic layers (15a and 17) and directly dated to the 6th millennium BC, initially morphologically identified as sheep, were revealed to be deer through ZooMS; deer are clearly distinguishable from sheep, with the peptide Col1AT40 displaying a *m/z* of 1550.8 instead of 1580.8 for the latter (Buckley et al. 2009).

Consequently, there is no secure identification of any domesticated animals in the early phases of ceramic use at Rakushechny Yar. Based on our current knowledge, Rakushechny Yar should therefore be considered as an entirely forager site during the Early Neolithic. It should be noted however that only a relatively small area along the shoreline at Rakushechny Yar has so far been excavated (*ca.* 90 m^2^) with little evidence for habitation structures.

### A clear boundary between the aquatic and agricultural Neolithic?

Both microscopy and chemical analysis of vessels from the Early Neolithic layers at Rakushechny Yar clearly shows a very strong association with fishing activities, particularly the processing of migratory fish, such as sturgeon. While some typological and stylistic details can be attributable to Early Neolithic agricultural settlements to the south and east, there is no evidence that the agricultural or pastoral economy was adopted at this site, as has previously been suggested (Belanovskaya 1995; Sablin 2018; Dolbunova et al. 2020b). Nevertheless, it is still plausible that knowledge of pottery production was gained through contact with farming communities but incorporated into an entirely fisher-hunter-gatherer economy. The other hypotheses are that early farmers moved to this region but abandoned food production in favor of intensive aquatic resource exploitation and adapted the use of pottery accordingly or that pottery production was innovated locally or was acquired from other foragers. Each of these hypotheses demands further investigation, particularly more precise ^14^C dating of sites, investigation of the dispersal dynamics and comparative typological assessment, on a regional and super-regional scale.

In particular, the potential acculturation of farmers into a foraging lifeway is gaining increasing traction through studies of this nature and should no longer be considered as anomalous. In the Iron Gates Gorge and in Southern Scandinavia, residue analysis has shown that Early Neolithic Starcevo and Funnel-beaker farming communities clearly turned their potcraft towards the processing of aquatic resources in addition to dairy and other produced foods (Craig et al. 2011; Cramp et al. 2019; Fig. 4b). Many of these sites were previously occupied in the Mesolithic and are situated along rivers, lakes and coasts with high aquatic productivity, particularly in seasonal aquatic foods.

It has been argued that agriculture, pastoralism and intensive fishing are delayed-return economies that require similar technological adaptations, including pottery, in anticipation of a predictable resource, and led to similar outcomes in terms of reducing mobility, and population growth (Oras et al. 2017). The evidence from Rakushechny Yar fits well with this model, given the seasonal nature of sturgeon exploitation. Careful advanced preparation would be needed to efficiently exploit this resource, including provision for processing and storage, prompting the need for pottery production. Ethnographic studies on native communities of coastal British Columbia show that different sizes of containers were used for the rendering oil from anadromous fish (Kuhnlein et al. 1982). This may also be the case for Rakushechny Yar where a diverse range of pottery containers were used to process aquatic products to maximize returns from this seasonal resource and perhaps facilitate its long-term storage and transportation. Evidence from the appearance of the charred deposits and their elemental composition further supports oil production as a likely function.

Finally, we found no evidence at Rakushechny Yar that the boundary between the aquatic and agricultural Neolithic was ‘permeable’. Perhaps the most likely hypothesis is that these semi-sedentary communities held very different world views and life-ways, which translated into separate notions regarding pottery production and usage. Investigations of pottery use and detailed archaeozoological analyses at other points of potential contact along the forager/farmer border are needed to confirm whether this pattern is sustained inter-regionally. Indeed, widespread analysis of 5th millenium BC forager pottery from the Baltic points to some exchange of produce between forager and farmers, at least in the western extreme prior to the wholescale adoption of farming (Courel et al. 2020).

## Supplementary Information

Below is the link to the electronic supplementary material.

Supplementary file1 (DOCX 1.43 KB)

## Data Availability

The authors confirm that the data supporting the findings of this study are available within the article and its supplementary materials.

## Code availability

Non applicable.
