# Supplementary material for: Neolithic farmers or Neolithic foragers? Organic residue analysis of early pottery from Rakushechny Yar on the Lower Don (Russia)
Source: Archaeol Anthropol Sci. 2021 Jul 26;13(8):141. doi: 10.1007/s12520-021-01412-2 (PMC8550616; doi:10.1007/s12520-021-01412-2)
Supplement: Supplementary file 1 — Supplementary file1 (DOCX 1.43 KB) [file 12520_2021_1412_MOESM1_ESM.docx]

**SUPPLEMENTARY MATERIAL**

**Table (S1): List of samples selected for lipid analysis (GCMS, GC-c-IRMS) and bulk isotope characteristics of charred deposits (EA-IRMS).**

| Sample | Type | Phase | Layers | Lipid conc. (µg g^-1^) | Major compounds detected | volume | SRR  (%) | APAA C_20_/C_18_ | δ^13^C_16 :0_ (‰) | δ^13^C_18 :0_ (‰) | Δ^13^C  (C_18:0_-C_16:0_) | %C | δ^13^ C  (‰) | %N | δ^15^N  (‰) | C:N |
| --- | --- | --- | --- | --- | --- | --- | --- | --- | --- | --- | --- | --- | --- | --- | --- | --- |
| Raku-86 | Foodcrust int | EN | 20 | 1375.8 | SFA (C_9:0-24:0_), UFA (C_15:1, 16:1,18:1, 20:1, 22:1_), DC (C_9-13_), br, terp, APAA (C_16, 18, 20_), tmtd, phy, pri |  | n/a |  |  |  |  | 24.8 | -25.4 | 3.7 | 9.1 | 8.1 |
| Raku-87 | Foodcrust int | EN | 20 | 1894.0 | SFA (C_14:0-24:0_), UFA (C_15:1-18:1, 20:1, 22:1, 24:1_), DC (C_11-13_), br, terp, APAA (C_16, 18, 20_), chol, tmtd, phy, pri | \|  \| \| --- \| \|  \| |  |  | -29.4 | -29.8 | -0.4 | 8.1 | -26.2 | 0.8 | 10.2 | 11.0 |
| Raku-88 | Foodcrust int^1^ | EN | 20 | 4733.3 | SFA (C_12:0-24:0_), UFA (C_14:1-16:1, 18:1, 22:1_), DC (C_9-13_), br, terp, APAA (C_16, 18, 20_), chol, tmtd, phy, pri | \|  \| \| --- \| \|  \| |  |  | -28.9 | -28.5 | 0.4 | 13.5 | -26.4 | 1.6 | 10.2 | 9.9 |
| Raku-89 | Foodcrust int^1^ | EN | 14 | 1812.2 | SFA (C_12:0-20:0_), UFA (C_15:1, 16:1, 18:1, 20:1, 22:1_), br, terp, APAA (C_16, 18, 20_), tmtd, phy, pri |  |  | 0.27 | -28.6 | -29.7 | -1.1 | 25.9 | -25.2 | 2.3 | 9.8 | 12.9 |
| Raku-90 | Ceramic | EN | 14 | 275.6 | SFA (C_11:0-26:0_), UFA (C_14:1-16:1,18:1, 20:1,22:1_), DC (C_8-16_), br, Alk (C_15, 16, 18, 20_), terp, HAP, APAA (C_16, 18, 20_), tmtd, phy, pri |  | 32.5 | 0.16 | -25.5 | -25.6 | -0.1 |  |  |  |  |  |
|  | Foodcrust int | EN | 14 | 1461.9 | SFA (C_14:0-24:0_), UFA (C_14:1, 16:1, 18:1, 22:1, 24:1_), DC (C_13_) (tr), br, terp, APAA (C_16, 18, 20_), tmtd, phy, pri |  |  | 0.30 | -27.5 | -28.5 | -0.9 | 10.6 | -25.7 | 0.8 | 10.5 | 15.9 |
| Raku-91 | Foodcrust int | EN | 14 | 2864.6 | SFA (C_14:0-24:0_), UFA (C_14:1-16:1 18:1, 20:1, 22:1, 24:1_), DC (C_13_), br, terp, APAA (C_16, 18, 20_), chol (tr), tmtd, phy, pri |  | tr | 0.44 | -28.1 | -28.7 | -0.7 | 6.8 | -25.2 | 0.7 | 11.1 | 10.9 |

| Sample | Type | Phase | Layers | Lipid conc. (µg g^-1^) | Major compounds detected | volume | SRR  (%) | APAA C_20_/C_18_ | δ^13^C_16 :0_ (‰) | δ^13^C_18 :0_ (‰) | Δ^13^C  (C_18:0_-C_16:0_) | %C | δ^13^ C  (‰) | %N | δ^15^N  (‰) | C:N |
| --- | --- | --- | --- | --- | --- | --- | --- | --- | --- | --- | --- | --- | --- | --- | --- | --- |
| Raku-92 | Ceramic | EN | 14-15 | 42.4 | SFA (C_9:0-26:0_), UFA (C_14:1, 16:1, 18:1, 22:1_), Alk (C_15-18, 20, 22-24, 26-28_), br, terp*, HAP, APAA (C_16, 18, 20_), tmtd, phy, pri |  |  |  |  |  |  |  |  |  |  |  |
|  | Foodcrust int^1^ | EN | 14-15 | 1510.0 | SFA (C_12:0-24:0_), UFA (C_16:1, 18:1, 22:1_), DC (C_9-11, 13_), br, terp, APAA (C_16, 18, 20_), tmtd, phy, pri |  | 63.2 | 0.02 | -28.8 | -28.5 | 0.3 | 27.2 | -28.5 | 2.2 | 10.1 | 15.8 |
| Raku-93 | Ceramic | EN | 14-15 | 379.6 | SFA (C_9:0-26:0_), UFA (C_16:1, 18:1, 22:1_), DC (C_7-16_), br, terp, APAA (C_16, 18, 20_), tmtd, phy, pri |  | 52.5 | 0.14 | -24.6 | -26.1 | -1.5 |  |  |  |  |  |
|  | Foodcrust int | EN | 14-15 | 870.0 | SFA (C_14:0-26:0_), UFA (C_15:1, 16:1, 18:1, 20:1, 22:1, 24:1_), DC (C_13_), Alk (C_14-20_), Alkone (16-K_31_), br, terp, APAA (C_16, 18, 20_), tmtd, phy, pri |  | tr |  | -27.9 | -28.8 | -0.9 | 22.9 | -25.8 | 2.1 | 7.5 | 12.7 |
| Raku-94 | Foodcrust int | EN | 14-15 | 885.7 | SFA (C_14:0-24:0_), UFA (C_16:1, 18:1, 20:1, 24:1_), br, terp, APAA (C_16, 18, 20_), tmtd, phy, pri |  |  |  | -27.8 | -29.2 | -1.5 |  |  |  |  |  |
| Raku-95 | Ceramic | EN | 14 | 53.5 | SFA (C_12:0-24:0_), UFA (C_14:1, 16:1, 18:1, 22:1_), Alk (C_15-17, 19, 20_), br, terp, APAA (C_16, 18, 20_), tmtd, phy, pri |  |  |  | -29.3 | -29.1 | 0.2 |  |  |  |  |  |
|  | Foodcrust int | EN | 14 | 1339.5 | SFA (C_14:0-24:0_), UFA (C_16:1, 18:1, 20:1, 22:1, 24:1_), DC (C_13_), br, terp, APAA (C_16, 18, 20_), tmtd, phy, pri |  |  | 0.19 | -28.3 | -29.0 | -0.7 | 26.9 | -26.8 | 2.8 | 10.1 | 10.8 |
| Raku-96 | Ceramic^1^ | EN | 12 | 38.8 | SFA (C_12:0-26:0_), UFA (C_16:1, 18:1, 22:1_), DC (C_11, 12_), Alk (C_17, 18, 20-24_), br, terp*, HAP, APAA (C_16, 18, 20_), tmtd, phy, pri | c | 59.8 | 0.27 | -27.6 | -27.6 | 0.0 |  |  |  |  |  |
|  | Foodcrust int | EN | 12 | 10.8 | SFA (C_14:0-26:0_), UFA (C_15:1, 16:1, 18:1, 18:2, 20:1, 22:1, 24:1_), DC (C_13_), br, terp, APAA (C_16, 18, 20_), tmtd, phy, pri |  |  |  | -27.3 | -28.1 | -0.9 | 19.8 | -25.3 | 2.4 | 14.1 | 10.0 |

| Sample | Type | Phase | Layers | Lipid conc. (µg g^-1^) | Major compounds detected | volume | SRR  (%) | APAA C_20_/C_18_ | δ^13^C_16 :0_ (‰) | δ^13^C_18 :0_ (‰) | Δ^13^C  (C_18:0_-C_16:0_) | %C | δ^13^ C  (‰) | %N | δ^15^N  (‰) | C:N |
| --- | --- | --- | --- | --- | --- | --- | --- | --- | --- | --- | --- | --- | --- | --- | --- | --- |
| Raku-97 | Ceramic | EN | 12 | 115.8 | SFA (C_12:0-26:0_), UFA (C_16:1, 18:1, 22:1_), DC (C_9, 11_), Alk (C_15, 16, 18-24_), br, terp, APAA (C_16, 18, 20_), tmtd, phy, pri |  | 58.0 | 0.24 | -28.8 | -28.0 | 0.8 |  |  |  |  |  |
|  | Foodcrust int | EN | 12 | 2457.7 | SFA (C_14:0-26:0_), UFA (C_15:1, 16:1, 18:1, 20:1, 22:1, 24:1_), DC (C_13_), br, terp, APAA (C_16, 18, 20_), tmtd, phy, pri |  |  | 0.24 | -27.4 | -28.2 | -0.8 | 10.3 | -23.8 | 1.1 | 10.8 | 12.2 |
| Raku-98 | Foodcrust int | EN | 12 | 1557.7 | SFA (C_14:0-30:0_), UFA (C_14:1-16:1, 18:1, 20:1, 22:1, 24:1_), DC (C_13_), br, terp, APAA (C_16, 18, 20_), tmtd, phy, pri |  | tr | 0.28 | -28.1 | -27.8 | 0.3 | 18.4 | -25.5 | 1.4 | 11.5 | 15.1 |
| Raku-99 | Ceramic^1^ | EN | 12 | 118.8 | SFA (C_8:0-26:0_), UFA (C_16:1, 18:1, 22:1_), DC (C_9_), Alk(C_15-24, 28, 29_), br, terp, HAP, APAA (C_16, 18, 20_), tmtd, phy, pri |  | tr |  | -27.9 | -27.8 | 0.1 |  |  |  |  |  |
|  | Foodcrust int | EN | 12 | 2052.1 | SFA (C_12:0-24:0_), UFA (C_14:1-16:1, 18:1, 20:1, 22:1, 24:1_), DC (C_13_), br, terp, APAA (C_16, 18, 20_), tmtd, phy, pri |  |  |  | -29.0 | -29.2 | -0.2 | 16.6 | -25.7 | 2.5 | 10.1 | 7.9 |
| Raku-100 | Foodcrust int | EN | 11 | 3223.1 | SFA (C_13:0-24:0_), UFA (C_14-16:1, 18:1, 20:1, 22:1, 24:1_), DC (C_13_), br, terp, APAA (C_16, 18, 20_), tmtd, phy, pri |  |  |  | -28.5 | -28.3 | 0.2 | 8.7 | -25.7 | 1.0 | 8.4 | 10.7 |
| Raku-101 | Foodcrust int | EN | 11 | 2369.0 | SFA (C_12:0-24:0_), UFA (C_14:1-18:1, 20:1, 22, 24:1_), DC (C_11, 13_), br, terp, APAA (C_16, 18, 20_), tmtd, phy, pri |  |  | 0.17 | -28.5 | -28.5 | 0.0 | 20.2 | -25.8 | 1.8 | 10.4 | 13.1 |
| Raku-102 | Foodcrust int | EN | 11 | 1590.0 | SFA (C_13:0-26:0_), UFA (C_14:1-16:1, 18:1, 20:1, 22:1, 24:1_), DC (C_13_), br, terp, APAA (C_16, 18, 20_), tmtd, phy, pri |  |  |  | -28.7 | -29.1 | -0.4 | 17.2 | -26.2 | 2.2 | 11.7 | 8.7 |
| Raku-103 | Foodcrust int | EN | 11 | 929.5 | SFA (C_14:0-26:0_), UFA (C_15:1, 16:1, 18:1, 20:1, 22:1, 24:1_), DC (C_11-14_), br, terp, APAA (C_16, 18, 20_), tmtd, phy, pri |  | tr | 0.20 | -27.5 | -26.9 | 0.6 | 25.7 | -24.7 | 2.5 | 11.7 | 11.3 |

| Sample | Type | Phase | Layers | Lipid conc. (µg g^-1^) | Major compounds detected | volume | SRR  (%) | APAA C_20_/C_18_ | δ^13^C_16 :0_ (‰) | δ^13^C_18 :0_ (‰) | Δ^13^C  (C_18:0_-C_16:0_) | %C | δ^13^ C  (‰) | %N | δ^15^N  (‰) | C:N |
| --- | --- | --- | --- | --- | --- | --- | --- | --- | --- | --- | --- | --- | --- | --- | --- | --- |
| Raku-104 | Foodcrust int | EN | 11 | 2538.2 | SFA (C_14:0-24:0_), UFA (C_14:1-16:1, 18:1, 20:1, 24:1_), DC (C_13_) (tr), br, terp, APAA (C_16, 18, 20_), tmtd, phy, pri |  |  |  | -28.3 | -28.9 | -0.6 | 9.7 | -24.5 | 1.1 | 9.4 | 9.7 |
| Raku-105 | Foodcrust int | EN | 11 | 1969.8 | SFA (C_12:0-26:0_), UFA (C_14:1-16:1, 18:1, 20:1, 22:1, 24:1_), br, terp, APAA (C_16, 18, 20_), chol (tr), tmtd, phy, pri |  | 29.7 |  | -28.6 | -29.2 | -0.6 | 10.7 | -26.1 | 1.2 | 11.9 | 10.2 |
| Raku-106 | Ceramic | EN | 11 | 70.1 | SFA (C_10:0-26:0_), UFA (C_16:1, 18:1, 22:1_), Alk (C_15-24, 26, 27_),br, terp, HAP, APAA (C_16, 18, 20_), tmtd, phy, pri |  |  |  | -29.3 | -29.3 | 0.0 |  |  |  |  |  |
|  | Foodcrust int^1^ | EN | 11 | 1858.9 | SFA (C_14:0-20:0_), UFA (C_14:1, 16:1, 18:1, 22:1_), br, terp, APAA (C_16, 18, 20_), tmtd, phy, pri |  |  |  | -27.3 | -28.6 | -1.3 | 21.4 | -25.1 | 3.1 | 11.3 | 8.9 |
| Raku-107 | Foodcrust int^1^ | EN | 11 | 1523.2 | SFA (C_14:0-24:0_), UFA (C_16:1, 18:1, 20:1, 22:1, 24:1_), br, terp, APAA (C_16, 18, 20_), tmtd, phy, pri |  |  |  | -28.1 | -28.4 | -0.4 | 13.3 | -27.0 | 2.1 | 7.4 | 7.9 |
| Raku-108 | Ceramic int | EN | 11 | 32.4 | SFA (C_11:0-26:0_), UFA (C_16:1, 18:1, 22:1_), DC (C_9_), Alk (C_17-18, 22-24_), br, terp, APAA (C_16, 18, 20_), tmtd, phy, pri |  |  |  | -29.7 | -29.5 | 0.2 |  |  |  |  |  |
|  | Foodcrust int | EN | 11 | 1374.6 | SFA (C_12:0-26:0_), UFA (C_14:1-16:1, 18:1, 20:1, 22:1, 24:1_), DC (C_11, 13_), br, terp, APAA (C_16, 18, 20_), tmtd, phy, pri |  |  | 0.19 | -28.4 | -28.6 | -0.3 | 12.4 | -26.9 | 1.4 | 10.2 | 10.1 |
| Raku-109 | Foodcrust int | EN | 11 | 1257.1 | SFA (C_12:0-26:0_), UFA (C_14:1-18:1, 20:1, 22:1, 24:1_), DC (C_11, 13_), br, terp, APAA (C_16, 18, 20_), chol, tmtd, phy, pri |  | tr | 0.14 | -28.2 | -27.8 | 0.4 | 23.5 | -25.3 | 2.3 | 11.8 | 11.4 |
| Raku-110 | Foodcrust int | EN | 11 | 1498.3 | SFA (C_13:0-26:0_), UFA (C_14:1-18:1, 18:2, 20:1, 24:1_), DC (C_13_), br, terp, APAA (C_16, 18, 20_), tmtd, phy, pri |  |  |  | -28.7 | -30.1 | -1.4 | 7.1 | -27.8 | 0.5 | 6.5 | 15.1 |

| Sample | Type | Phase | Layers | Lipid conc. (µg g^-1^) | Major compounds detected | volume | SRR  (%) | APAA C_20_/C_18_ | δ^13^C_16 :0_ (‰) | δ^13^C_18 :0_ (‰) | Δ^13^C  (C_18:0_-C_16:0_) | %C | δ^13^ C  (‰) | %N | δ^15^N  (‰) | C:N |
| --- | --- | --- | --- | --- | --- | --- | --- | --- | --- | --- | --- | --- | --- | --- | --- | --- |
| Raku-111 | Foodcrust int | EN | 11 | 2242.4 | SFA (C_14:0-28:0_), UFA (C_14:1-16:1, 18:1, 20:1, 22:1, 24:1_), DC (C_13_), br, terp, APAA (C_16, 18, 20_), chol (tr), tmtd, phy, pri |  |  |  | -28.1 | -28.5 | -0.3 | 11.1 | -25.5 | 1.5 | 9.8 | 8.4 |
| Raku-112 | Ceramic^1^ | EN | 11 | 73.3 | SFA (C_16:0-24:0_), UFA (C_16:1, 18:1, 22:1_), Alk (C_22-24, 26-29_),br, terp, APAA (C_16, 18, 20_), tmtd, phy, pri |  |  |  | -29.9 | -29.6 | 0.3 |  |  |  |  |  |
| Raku-113 | Foodcrust int | EN | 11 | 1256.5 | SFA (C_11:0-24:0_), UFA (C_14-16:1, 18:1, 20:1, 22:1_), DC (C_7-12_), br, terp, APAA (C_16, 18, 20_), tmtd, phy, pri |  |  |  |  |  |  | 28.0 | -26.2 | 2.8 | 10.5 | 11.8 |
| Raku-114 | Ceramic | EN | 11 | 26.2 | SFA (C_11:0-28:0_), UFA (C_16:1, 18:1_), DC (C_9_), Alk (C_20-30_), br, terp*, HAP, APAA (C_16, 18, 20_), tmtd, phy, pri |  |  |  | -29.9 | -29.8 | 0.2 |  |  |  |  |  |
|  | Foodcrust int^1^ | EN | 11 | 1306.5 | SFA (C_14:0-24:0_), UFA (C_14:1-16:1, 18:1, 20:1, 22:1, 24:1_), br, terp, APAA (C_16, 18, 20_), tmtd, phy, pri |  |  |  | -29.3 | -29.9 | -0.6 | 9.7 | -30.3 | 0.7 | 6.8 | 16.1 |
| Raku-115 | Foodcrust int^1^ | EN | 11 | 1830.6 | SFA (C_12:0-19:0_), UFA (C_14:1-16:1, 18:1, 20:1, 20:1, 22:1, 24:1_), DC (C_9-13_), br, terp, APAA (C_16, 18, 20_), tmtd, phy, pri |  |  |  | -29.0 | -30.3 | -1.3 | 15.0 | -25.6 | 2.1 | 10.6 | 8.5 |
| Raku-116 | Foodcrust int | EN | 11 | 743.2 | SFA (C_8:0-25:0_), UFA (C_14:1-16:1, 18:1, 20:1, 22:1, 24:1_), DC (C_6-13_), br, terp, APAA (C_16, 18, 20_), tmtd, phy, pri |  |  |  |  |  |  | 20.8 | -23.6 | 2.1 | 12.6 | 11.4 |
| Raku-151 | Ceramic^1^ | EN | 11 | 108.2 | SFA (C_8:0-23:0_), UFA (C_16:1, 18:1_) (tr), Alk (C_20-30_), br, terp, HAP, tmtd, phy, pri (tr) |  | tr |  | -28.5 | -29.2 |  |  |  |  |  |  |
|  | Foodcrust int | EN | 11 | 931.6 | SFA (C_9:0-24:0_), UFA (C_16:1, 18:1, 22:1_), DC (C_9-13_), Alk (C_20-38_), br, terp*, tmtd (tr), phy |  | tr |  | -29.1 | -29.3 |  | 2.3 | -27.7 | 0.1 | 6.4 | 31.7 |
| Raku-152 | Ceramic^1^ | EN | 11 | 173.7 | SFA (C_9:0-26:0_), UFA (C_16:1, 18:1, 20:1, 22:1, 24:1_), DC (C_7, 9-14_), Alk (C_15-17, 22-24, 27-30_), br, terp*, HAP, APAA (C_16, 18, 20_), tmtd, phy, pri | e | 47.9 |  | -27.5 | -28.7 | -1.2 |  |  |  |  |  |
| Sample | Type | Phase | Layers | Lipid conc. (µg g^-1^) | Major compounds detected | volume | SRR  (%) | APAA C_20_/C_18_ | δ^13^C_16 :0_ (‰) | δ^13^C_18 :0_ (‰) | Δ^13^C  (C_18:0_-C_16:0_) | %C | δ^13^ C  (‰) | %N | δ^15^N  (‰) | C:N |
| Raku-153 | Ceramic | EN | 20 | 286.1 | SFA (C_6:0-26:0_), UFA (C_14:1-16:1, 18:1, 20:1, 22:1_), DC (C_6, 7, 9-12_), Alk (C_15-18, 22-24_), br, terp*, HAP, APAA (C_16, 18, 20_), chol (tr), tmtd, phy, pri |  | 57.8 |  | -25.7 | -26.4 | -0.7 |  |  |  |  |  |
| Raku-154 | Ceramic | EN | 23 | 41.3 | SFA (C_12:0-28:0_), UFA (C_15:1-16:1, 18:1, 22:1_), DC (C_11-13_), Alk (C_16, 20, 22-24_), br, terp*, HAP, APAA (C_16, 18, 20_), tmtd, phy, pri | e |  |  | -28.0 | -28.7 | -0.7 |  |  |  |  |  |
| Raku-155 | Ceramic | EN | 19 | 622.8 | SFA (C_8:0-28:0_), UFA (C_16:1, 18:1, 20:1, 22:1_), DC (C_8, 9, 11, 18_), Alk (C_14-24_), br, terp*, HAP, APAA (C_16, 18, 20_), chol (tr), tmtd, phy, pri |  | tr |  | -26.6 | -27.4 | -0.7 |  |  |  |  |  |
| Raku-156 | Ceramic^1^ | EN | 19 | 684.6 | SFA (C_14:0-26:0_), UFA (C_14:1-16:1, 18:1, 20:1, 22:1_), DC (C_11, 13_), Alk (C_22, 24_), br, terp, APAA (C_16, 18, 20_), tmtd, phy, pri |  |  |  | -28.9 | -28.9 | 0.0 |  |  |  |  |  |
| Raku-157 | Ceramic | EN | 14 | 105.9 | SFA (C_11:0-26:0_), UFA (C_16:1, 18:1, 20:1, 22:1_), DC (C_9-15_), Alk (C16, 22-24), br, terp, APAA (C_16, 18, 20_), tmtd, phy, pri |  | 45.5 | 0.14 | -26.7 | -27.7 | -1.0 |  |  |  |  |  |
| Raku-158 | Ceramic | EN | 14 | 80.6 | SFA (C_11:0-24:0_), UFA (C_14:1-16:1, 18:1, 20:1, 22:1_), DC (C_9-15_), Alk (C_18, 22, 23_), br, terp, HAP, APAA (C_16, 18, 20_), tmtd, phy, pri | d | 62.9 | 0.26 | -26.2 | -26.5 | -0.3 |  |  |  |  |  |
| Raku-159 | Ceramic | EN | 16 | 264.6 | SFA (C_6:0-28:0_), UFA (C_14:1-16:1, 20:1, 22:1_), DC (C_9-14_), Alk (C15-33), br, terp*, HAP, APAA (C_16, 18, 20_), tmtd, phy, pri | c | 68.8 | 0.34 | -25.7 | -26.1 | -0.4 |  |  |  |  |  |
| Raku-258 | Ceramic | EN | (17-19?) | 18.2 | SFA (C_14:0-28:0_), UFA (C_16:1, 18:1, 22:1_), DC (C_11, 13_), br, APAA (C_16, 18, 20_), tmtd, phy, pri | e | 58.2 | 0.24 | -26.7 | -27.2 | -0.5 |  |  |  |  |  |

| Sample | Type | Phase | Layers | Lipid conc. (µg g^-1^) | Major compounds detected | volume | SRR  (%) | APAA C_20_/C_18_ | δ^13^C_16 :0_ (‰) | δ^13^C_18 :0_ (‰) | Δ^13^C  (C_18:0_-C_16:0_) | %C | δ^13^ C  (‰) | %N | δ^15^N  (‰) | C:N |
| --- | --- | --- | --- | --- | --- | --- | --- | --- | --- | --- | --- | --- | --- | --- | --- | --- |
| Raku-259 | Ceramic | EN | (17-19?) | 83.9 | SFA (C_10:0-30:0_), UFA (C_16:1, 18:1, 20:1, 22:1, 24:1_), DC (C_8-15_), Alk (C_27, 29_), Alkone (10-K_29_), br, APAA (C_16, 18, 20_), tmtd, phy, pri |  | 50.3 |  | -26.6 | -26.7 | -0.1 |  |  |  |  |  |
| Raku-260 | Ceramic | EN | (17-19?) | 42.5 | SFA (C_12:0-26:0_), UFA (C_16:1, 18:1, 22:1_), DC (C_9-15_), br, APAA (C_16, 18, 20_), tmtd, phy, pri |  | 56.9 | 0.22 | -25.6 | -26.6 | -1.0 |  |  |  |  |  |
| Raku-261 | Ceramic | EN | pit 2 (11-14?) | 65.6 | SFA (C_11:0-26:0_), UFA (C_18:1, 20:1_), DC (C_9-16_), br, APAA (C_16, 18, 20_), tmtd, phy, pri |  | 31.2 | 0.18 | -23.5 | -23.7 | -0.2 |  |  |  |  |  |
| Raku-262 | Ceramic | EN | pit 2 (11-14?) | 19.1 | SFA (C_14:0-26:0_), UFA (C_18:1_), DC (C_11-15_), br, APAA (C_16, 18, 20_), tmtd, phy, pri | e | 23.6 |  | -25.8 | -26.6 | -0.8 |  |  |  |  |  |
| Raku-263 | Ceramic | EN | vivip 3 | 65.3 | SFA (C_14:0-26:0_), UFA (C_16:1, 18:1, 20:1_), DC (C_11_), br, APAA (C_16, 18, 20_), tmtd, phy, pri | d | 54.6 | 0.19 | -26.9 | -27.3 | -0.4 |  |  |  |  |  |
| Raku-264 | Ceramic^1^ | EN | 20 | 1.1 | SFA (C_14:0-24:0_), UFA (C_18:1, 22:1_), Alk (C19-25), br, terp, APAA (C_16, 18, 20_), tmtd, phy, pri |  | tr |  | -27.2 | -29.1 | -2.0 |  |  |  |  |  |
| Raku-265 | Ceramic | EN | 20 | 16.4 | SFA (C_12:0-24:0_), UFA (C_18:1, 22:1_), DC (C_9_) (tr), Alk (C18-24), br, APAA (C_16, 18, 20_), tmtd, phy, pri | e | tr |  | -28.0 | -27.9 | 0.1 |  |  |  |  |  |
| Raku-266 | Ceramic | EN | 13 | 92.0 | SFA (C_12:0-26:0_), UFA (C_16:1, 18:1, 22:1_), DC (C_9-13_), Alk (C15-24), br, terp, HAP (tr), APAA (C_16, 18, 20_), tmtd, phy, pri |  | 57.0 | 0.19 | -26.3 | -26.5 | -0.2 |  |  |  |  |  |
| Raku-267 | Ceramic | EN | 20 | 34.2 | SFA (C_13:0-24:0_), UFA (C_18:1, 22:1_), DC (C_11_), Alk (C_16- 24, 26, 27_), br, terp, HAP, APAA (C_16, 18, 20_), tmtd, phy, pri | b | 71.0 |  | -27.0 | -27.4 | -0.4 |  |  |  |  |  |
| Raku-268 | Ceramic | EN | 21 | 1.7 | SFA (C_14:0-24:0_), UFA (C_16:1, 18:1, 22:1_), DC (C_13_), Alk (C_20-24_), br, terp, HAP, APAA (C_16, 18, 20_), tmtd, phy, pri | e |  |  | -28.6 | -29.3 | -0.6 |  |  |  |  |  |

| Sample | Type | Phase | Layers | Lipid conc. (µg g^-1^) | Major compounds detected | volume | SRR  (%) | APAA C_20_/C_18_ | δ^13^C_16 :0_ (‰) | δ^13^C_18 :0_ (‰) | Δ13C  (C_18:0_-C_16:0_) | %C | δ^13^ C  (‰) | %N | δ^15^N  (‰) | C:N |
| --- | --- | --- | --- | --- | --- | --- | --- | --- | --- | --- | --- | --- | --- | --- | --- | --- |
| Raku-269 | Ceramic^1^ | EN | 20 | 620.4 | SFA (C_12:0-29:0_), UFA (C_16:1, 18:1, 22:1_), DC (C_9, 11_), Alk (C_16-33_), br, terp, HAP, APAA (C_16, 18, 20_), tmtd, phy, pri |  |  |  | -29.2 | -29.0 | 0.2 |  |  |  |  |  |
| Raku-270 | Ceramic | EN | 20 | 111.6 | SFA (C_12:0-26:0_), UFA (C_16:1, 18:1, 20:1, 22:1_), DC (C_9-15_), Alk (C_16-29_), br, terp, HAP, APAA (C_16, 18, 20_), tmtd, phy, pri | f | 38.6 | 0.21 | -25.0 | -25.9 | -0.8 |  |  |  |  |  |
| Raku-271 | Ceramic^1^ | EN | 20 | 52.6 | SFA (C_14:0-28:0_), UFA (C_16:1, 18:1_), DC (C_11_) (tr), Alk (C17-33), br, terp, HAP, APAA (C_16, 18, 20_), tmtd, phy, pri | d |  | 0.26 | -28.0 | -27.9 | 0.1 |  |  |  |  |  |
| Raku-272 | Ceramic^1^ | EN | 20 | 113.9 | SFA (C_11:0-26:0_), UFA (C_16:1, 18:1, 22:1_), DC (C_9_), Alk (C_16-27_), br, terp, APAA (C_16, 18, 20_), tmtd, phy, pri | b |  |  | -27.1 | -27.6 | -0.5 |  |  |  |  |  |
| Raku-273 | Ceramic | EN | 21 | 364.6 | SFA (C_14:0-26:0_), UFA (C_16:1, 18:1_), DC (C_11_), Alk (C_17-33_), br, terp, APAA (C_16, 18, 20_), tmtd, phy, pri |  | 27.3 |  | -26.4 | -27.8 | -1.4 |  |  |  |  |  |
| Raku-274 | Ceramic^1^ | EN | 21 | 37.6 | SFA (C_14:0-28:0_), UFA (C_16:1, 18:1_), Alk (C_17-31_), br, terp, APAA (C_16, 18, 20_), tmtd, phy, pri | e |  |  | -28.7 | -29.3 | -0.6 |  |  |  |  |  |
| Raku-275 | Ceramic | EN | 20 | 66.2 | SFA (C_12:0-26:0_), UFA (C_16:1, 18:1_), DC (C_9-16_), Alk (C_16-29_), br, terp*, HAP, APAA (C_16, 18, 20_), tmtd, phy, pri | c | 53.0 | 0.20 | -25.5 | -25.7 | -0.3 |  |  |  |  |  |
| Raku-276 | Ceramic | EN | 19 | 79.6 | SFA (C_12:0-26:0_), UFA (C_16:1, 18:1_) (tr), DC (C_9-16_), Alk (C_17, 18, 20-32_), br, terp*, HAP, APAA (C_16, 18, 20_), tmtd, phy, pri | c | 59.2 | 0.19 | -26.0 | -26.7 | -0.7 |  |  |  |  |  |
| Raku-277 | Ceramic^1^ | EN | 19 | 33.6 | SFA (C_12:0-24:0_), Alk (C17-29), br, terp, APAA (C_16, 18, 20_), tmtd, phy, pri | e |  |  | -28.6 | -28.6 | 0.1 |  |  |  |  |  |
| Raku-278 | Ceramic | EN | 15a | 240.3 | SFA (C_8:0-28:0_), UFA (C_16:1, 18:1_), DC (C_9_), Alk (C_15-25_), br, terp*, HAP, APAA (C_16, 18, 20_), tmtd, phy, pri | c | 50.6 | 0.26 | -25.7 | -25.8 | -0.1 |  |  |  |  |  |
| Sample | Type | Phase | Layers | Lipid conc. (µg g^-1^) | Major compounds detected | volume | SRR  (%) | APAA C_20_/C_18_ | δ^13^C_16 :0_ (‰) | δ^13^C_18 :0_ (‰) | Δ^13^C  (C_18:0_-C_16:0_) | %C | δ^13^ C  (‰) | %N | δ^15^N  (‰) | C:N |
| Raku-279 | Ceramic | EN | 13a | 98.1 | SFA (C_13:0-26:0_), UFA (C_18:1, 20:1, 22:1, 24:1_), DC (C_11-15_), Alk (C_17-24_), br, terp, HAP, APAA (C_16, 18, 20_), tmtd, phy, pri | e | 33.0 |  | -26.8 | -26.8 | 0.0 |  |  |  |  |  |
| Raku-280 | Ceramic | EN | 15a | 119.7 | SFA (C_11:0-24:0_), DC (C_8-16_), Alk (C_15-33_), br, terp, HAP, APAA (C_16, 18, 20_), tmtd, phy, pri | f | 54.0 | 0.22 | -25.9 | -25.7 | 0.2 |  |  |  |  |  |
| Raku-281 | Ceramic | EN | 14-15 | 13.3 | SFA (C_13:0-24:0_), UFA (C_22:1_), Alk (C_17-25_), br, terp, APAA (C_16, 18, 20_), tmtd, phy, pri | e | tr |  | -27.5 | -27.1 | 0.4 |  |  |  |  |  |
| Raku-282 | Ceramic | EN | 13 | 22.5 | SFA (C_12:0-25:0_), UFA (C_18:1, 22:1_), Alk (C_17-24, 26_), br, terp, APAA (C_16, 18, 20_), tmtd, phy, pri | d | 17.4 |  | -26.7 | -27.1 | -0.4 |  |  |  |  |  |
| Raku-283 | Ceramic | EN | 10 | 109.5 | SFA (C_13:0-32:0_), UFA (C_16:1, 18:1, 20:1_), DC (C_10-15_), Alk (C_17-31_), br, terp, APAA (C_16, 18, 20_), tmtd, phy, pri |  | 26.1 | 0.18 | -27.0 | -27.4 | -0.3 |  |  |  |  |  |
| Raku-284 | Ceramic | EN | 11 | 16.6 | SFA (C_14:0-26:0_), UFA (C_18:1, 22:1_), Alk (C_17-24, 26_), br, terp, APAA (C_16, 18, 20_), tmtd, phy, pri | b | 41.3 |  | -28.1 | -29.2 | -1.1 |  |  |  |  |  |
| Raku-286 | Ceramic | EN | 11 | 125.6 | SFA (C_14:0-26:0_), UFA (C_16:1, 18:1, 20:1, 22:1_), DC (C_11_), Alk (C_22-25_), br, terp, APAA (C_16, 18, 20_), tmtd, phy, pri | b | 75.3 |  | -26.0 | -26.6 | -0.6 |  |  |  |  |  |
| Raku-287 | Ceramic | EN | 11 | 110.2 | SFA (C_8:0-26:0_), UFA (C_18:1_) (tr), DC (C_9-11_), Alk (C_16-29_), br, terp*, HAP, APAA (C_16, 18, 20_), tmtd, phy, pri | b |  |  | -27.5 | -28.4 | -0.9 |  |  |  |  |  |
| Raku-288 | Ceramic | EN | 12 | 40.5 | SFA (C_14:0-28:0_), UFA (C_16:1, 18:1_), DC (C_11_), Alk (C_17-28_), Alkone (16-K_31_), br, terp*, HAP (tr), APAA (C_16, 18, 20_), tmtd, phy, pri |  | 68.9 |  |  |  |  |  |  |  |  |  |
| Raku-289 | Ceramic^1^ | EN | 14 | 57.8 | SFA (C_14:0-26:0_), UFA (C_15, 16:1, 18:1, 22:1_), Alk (C_17-33_), br, terp, HAP (tr), APAA (C_16, 18, 20_), tmtd, phy, pri | b |  |  | -27.2 | -28.6 | -1.3 |  |  |  |  |  |
| Sample | Type | Phase | Layers | Lipid conc. (µg g^-1^) | Major compounds detected | volume | SRR  (%) | APAA C_20_/C_18_ | δ^13^C_16 :0_ (‰) | δ^13^C_18 :0_ (‰) | Δ^13^C  (C_18:0_-C_16:0_) | %C | δ^13^ C  (‰) | %N | δ^15^N  (‰) | C:N |
| Raku-913 | Ceramic | EN | Grey sand | 61.0 | SFA (C_12:0-30:0_), UFA (C_16:1, 18:1, 22:1_), DC (C_7-15_), HAP, 9-10diHFA (C_18_), br, APAA (C_16, 18, 20_), chol, tmtd, phy, pri | c | 44.6 | 0.18 | -27.0 | -27.6 | -0.6 |  |  |  |  |  |
|  | Foodcrust ext | EN | Grey sand | 306.9 | SFA (C_11:0-30:0_), UFA (C_18:1, 22:1_) (tr), DC (C_7-13_), br, HAP, APAA (C_16, 18, 20_), tmtd, phy, pri |  | 48.2 | 0.17 | -27.4 | -26.6 | 0.7 | 43.2 | -26.1 | 3.2 | 7.4 | 15.8 |
| Raku-914 | Ceramic | EN | vivip 2 | 65.3 | SFA (C_11:0-26:0_), UFA (C_16:1, 18:1, 22:1_), DC (C_8-13_), 9-10diHFA (C_18_), br, HAP, APAA (C_16, 18, 20_), chol, tmtd, phy, pri | a | 29.5 | 0.45 | -27.4 | -28.0 | -0.7 |  |  |  |  |  |
|  | Foodcrust int | EN | vivip 2 | n/a | pas d'extraction mais bulk/ not enough sample for extraction |  |  |  |  |  |  | 25.0 | -25.7 | 1.2 | 10.6 | 23.7 |
| Raku-915 | Ceramic | EN | 17-19 | 109.4 | SFA (C_11:0-26:0_), UFA (C_16:1, 18:1, 20:1, 22:1, 24:1_), DC (C_8-12_), Alk (C_14-17_), Alkone (16-K_31_), br, HAP, APAA (C_16, 18, 20_), chol (tr), tmtd, phy, pri | c | 42.3 | 0.27 | -25.9 | -26.4 | -0.6 |  |  |  |  |  |
|  | Foodcrust int | EN | 17-19 | 85.7 | SFA (C_14:0-24:0_), UFA (C_16:1, 18:1_) (tr), DC (C_9-12_), br, APAA (C_16, 18, 20_), tmtd, phy, pri |  | tr |  | -24.9 | -24.8 | 0.1 | 23.1 | -27.2 | 2.4 | 8.2 | 11.2 |
| Raku-916 | Ceramic | EN | vivip 2 | 400.1 | SFA (C_12:0-22:0_), UFA (C_18:1, 22:1_), DC (C_9-11_), br, HAP, APAA (C_16, 18, 20_), chol, tmtd, phy, pri |  | 75.7 | 0.04 | -27.8 | -30.0 | -2.3 |  |  |  |  |  |
|  | Foodcrust int | EN | vivip 2 | 514.5 | SFA (C_10:0-30:0_), UFA (C_16:1, 18:1, 22:1_), DC (C_7-13_), br, APAA (C_16, 18, 20_), tmtd, phy, pri |  | 80.3 | 0.04 | -27.2 | -26.9 | 0.3 | 26.9 | -28.4 | 4.2 | 7.5 | 7.5 |
| Raku-917 | Ceramic | EN | vivip 2 | 713.8 | SFA (C_9:0-26:0_), UFA (C_18:1, 22:1_), DC (C_9-13_), br, terp, APAA (C_16, 18, 20_), Alkone (16-K_31_), tmtd, phy, pri |  |  | 0.03 | -26.1 | -29.1 | -3.1 |  |  |  |  |  |
|  | Foodcrust int | EN | vivip 2 | 519.7 | SFA (C_12:0-28:0_), UFA (C_16:1, 18:1, 22:1_), DC (C_8-15_), br, APAA (C_16, 18, 20_), tmtd, phy, pri |  | 78.8 | 0.07 | -26.0 | -28.0 | -2.0 | 22.7 | -27.8 | 3.1 | 8.3 | 8.6 |
| Sample | Type | Phase | Layers | Lipid conc. (µg g^-1^) | Major compounds detected | volume | SRR  (%) | APAA C_20_/C_18_ | δ^13^C_16 :0_ (‰) | δ^13^C_18 :0_ (‰) | Δ^13^C  (C_18:0_-C_16:0_) | %C | δ^13^ C  (‰) | %N | δ^15^N  (‰) | C:N |
| Raku-918 | Ceramic | EN | 17-19 | 11.4 | SFA (C_12:0-26:0_), UFA (C_16:1, 18:1, 20:1, 22:1_), DC (C_8-12_), br, HAP, APAA (C_16, 18, 20_), chol, tmtd, phy, pri | a |  |  | -26.5 | -27.7 | -1.2 |  |  |  |  |  |
| Raku-919 | Ceramic | EN | 17-19 | 214.0 | SFA (C_9:0-22:0_), UFA (C_16:1, 18:1, 22:1_), DC (C_6-13_), Alk (C_11-12, 14-17_), br, APAA (C_16, 18, 20_), chol, tmtd, phy, pri |  | 45.5 | 0.32 | -25.8 | -26.3 | -0.5 |  |  |  |  |  |
|  | Foodcrust int | EN | 17-19 | 233.8 | SFA (C_11:0-30:0_), UFA (C_16:1, 18:1, 22:1_), DC (C_7-13_), Alkone (16-K_31_), br, APAA (C_16, 18, 20_), chol, tmtd, phy, pri |  | 48.2 |  | -26.2 | -26.3 | -0.1 | 22.7 | -26.9 | 1.9 | 7.6 | 13.9 |
|  | Foodcrust ext | EN | 17-19 | 945.6 | SFA (C_7:0-30:0_), UFA (C_16:1, 18:1, 20:1, 22:1, 24:1_), DC (C_5-13_), Alk (C_11, 14, 15, 17_), br, HAP, APAA (C_16, 18, 20_), chol (tr), tmtd, phy, pri |  | 48.4 | 0.21 | -25.1 | -25.5 | -0.4 | 37.0 | -26.5 | 1.7 | 10.8 | 25.7 |
| Raku-920 | Ceramic | EN | 17-19 | 224.7 | SFA (C_9:0-24:0_), UFA (C_16:1, 18:1, 20:1, 22:1, 24:1_), DC (C7_-12_), Alkone (16-K_31_), br, HAP, APAA (C_16, 18, 20_), chol, tmtd, phy, pri | c | 41.4 | 0.23 | -25.0 | -25.4 | -0.3 |  |  |  |  |  |
|  | Foodcrust int | EN | 17-19 | 239.6 | SFA (C_9:0-28:0_), UFA (C_16:1, 18:1, 18:2, 20:1, 22:1, 24:1_), DC (C_7-14_), Alk (C_12-17-33_), br, APAA (C_16, 18, 20_), chol, tmtd, phy, pri |  | 53.0 | 0.30 | -24.9 | -24.9 | 0.0 | 27.9 | -25.6 | 3.6 | 11.3 | 9.0 |
| Raku-921 | Ceramic | EN | 21/23 | 596.2 | SFA (C_8:0-24:0_), UFA (C_16:1, 18:1, 18:2, 20:1, 22:1, 24:1_), DC (C_6-12_), Alk (C_16, 17_), br, APAA (C_16, 18, 20_), chol, phyol, tmtd, phy, pri | c | 58.7 | 0.34 | -26.3 | -27.0 | -0.7 |  |  |  |  |  |
|  | Foodcrust int | EN | 21/23 | 1826.6 | SFA (C_8:0-30:0_), UFA (C_16:1, 18:1, 20:1, 22:1, 24:1_), DC (C_5-14_), br, APAA (C_16, 18, 20_), chol, tmtd, phy, pri |  | 56.0 | 0.26 | -25.2 | -25.7 | -0.4 | 28.7 | -27.1 | 2.5 | 12.2 | 13.6 |
|  | Foodcrust ext | EN | 21/23 | 1562.2 | SFA (C_10:0-24:0_), UFA (C_16:1, 18:1, 22:1_), DC (C_6-12_), Alk (C15, 16, 17), br, HAP, APAA (C_16, 18, 20_), tmtd, phy, pri |  | 58.9 | 0.14 | -25.5 | -25.8 | -0.3 | 41.7 | -26.0 | 2.3 | 12.7 | 21.7 |
| Sample | Type | Phase | Layers | Lipid conc. (µg g^-1^) | Major compounds detected | volume | SRR  (%) | APAA C_20_/C_18_ | δ^13^C_16 :0_ (‰) | δ^13^C_18 :0_ (‰) | Δ^13^C  (C_18:0_-C_16:0_) | %C | δ^13^ C  (‰) | %N | δ^15^N  (‰) | C:N |
| Raku-922 | Ceramic | EN | 17-19 | 297.0 | SFA (C_11:0-28:0_), UFA (C_16:1, 18:1, 22:1_), DC (C_7-13_), Alk (C_14, 15, 16, 17_), br, HAP, APAA (C_16, 18, 20_), chol (tr), tmtd, phy, pri |  | 56.6 | 0.28 | -27.6 | -27.6 | -0.1 |  |  |  |  |  |
|  | Foodcrust int | EN | 17-19 | 77.8 | SFA (C_10:0-24:0_), UFA (C_16:1, 18:1, 22:1_), DC (C_6-13_), br, APAA (C_16, 18, 20_), chol (tr), tmtd, phy, pri |  |  |  | -28.0 | -27.9 | 0.0 | 16.2 | -30.2 | 1.6 | 7.4 | 11.6 |
| Raku-923 | Ceramic | EN | 21/23 | 91.2 | SFA (C_10:0-26:0_), UFA (C_16:1, 18:1, 18:1, 20:1, 22:1, 24:1_), DC (C_7-13_), br, APAA (C_16, 18, 20_), chol, phyol, tmtd, phy, pri | e | 60.6 |  | -26.7 | -27.5 | -0.8 |  |  |  |  |  |
| Raku-924 | Ceramic | EN | vivip 2 | 49.6 | SFA (C_12:0-28:0_), UFA (C_16:1, 18:1, 20:1, 22:1, 24:1_), DC (C_8-15_), br, APAA (C_16, 18, 20_), chol (tr), tmtd, phy, pri |  | 29.7 |  | -26.4 | -26.7 | -0.3 |  |  |  |  |  |
|  | Foodcrust int | EN | vivip 2 | 453.9 | SFA (C_9:0-30:0_), UFA (C_16:1, 18:1, 20:1, 22:1_), DC (C_7-13_), br, APAA (C_16, 18, 20_), tmtd, phy, pri |  | 37.6 | 0.22 | -25.9 | -25.5 | 0.5 | 37.7 | -26.2 | 1.1 | 10.9 | 40.2 |
| Raku-925 | Ceramic | EN | 21/23 | 167.2 | SFA (C_9:0-26:0_), UFA (C_16:1, 18:1, 20:1, 24:1_), br, HAP, APAA (C_16, 18, 20_), chol, tmtd, phy, pri | b | 49.6 | 0.24 | -24.2 | -25.8 | -1.6 |  |  |  |  |  |
| Raku-926 | Ceramic | EN | vivip 2 | 65.5 | SFA (C_12:0-30:0_), UFA (C_16:1, 18:1, 20:1, 22:1, 24:1_), DC (C_9, 11-15_), br, APAA (C_16, 18, 20_), chol, tmtd, phy, pri |  | 49.2 | 0.38 | -25.9 | -27.0 | -1.1 |  |  |  |  |  |
|  | Foodcrust ext | EN | vivip 2 | 3701.5 | SFA (C_8:0-28:0_), UFA (C_16:1, 18:1, 20:1_), DC (C_6-13_), Alk (C_17_), br, APAA (C_16, 18, 20_), chol, tmtd, phy, pri |  | 50.0 | 0.37 | -23.8 | -24.2 | -0.4 | 34.4 | -25.5 | 1.2 | 10.2 | 32.4 |
| Raku-927 | Ceramic | EN | Grey sand uder viv | 12.5 | SFA (C_11:0-28:0_), UFA (C_16:1, 18:1, 20:1, 22:1_) (tr), DC (C_8-14_), br, APAA (C_16, 18, 20_), chol, tmtd, phy, pri |  | 58.5 | 0.34 | -28.1 | -28.0 | 0.1 |  |  |  |  |  |
|  | Foodcrust int | EN | Grey sand uder viv | 1741.4 | SFA (C_9:0-26:0_), UFA (C_16:1, 18:1, 20:1, 22:1_), DC (C_6-14_), br, APAA (C_16, 18, 20_), chol, tmtd, phy, pri |  | 62.3 | 0.24 | -28.1 | -27.9 | 0.3 | 37.1 | -28.4 | 4.3 | 9.6 | 10.0 |

| Sample | Type | Phase | Layers | Lipid conc. (µg g^-1^) | Major compounds detected | volume | SRR  (%) | APAA C_20_/C_18_ | δ^13^C_16 :0_ (‰) | δ^13^C_18 :0_ (‰) | Δ^13^C  (C_18:0_-C_16:0_) | %C | δ^13^ C  (‰) | %N | δ^15^N  (‰) | C:N |
| --- | --- | --- | --- | --- | --- | --- | --- | --- | --- | --- | --- | --- | --- | --- | --- | --- |
| Raku-928 | Ceramic | EN | vivip 1 | 63.4 | SFA (C_12:0-28:0_), UFA (C_16:1, 18:1, 20:1, 22:1_), DC (C_9-12_), br, APAA (C_16, 18, 20_), chol, tmtd, phy, pri | c | tr |  | -25.1 | -26.8 | -1.7 |  |  |  |  |  |
| Raku-929 | Ceramic | EN | vivip 1 | 72.2 | SFA (C_10:0-28:0_), UFA (C_16:1, 18:1, 20:1, 22:1, 24:1_), DC (C_7-15_), br, APAA (C_16, 18, 20_), chol, tmtd, phy, pri | b | 24.8 | 0.42 | -25.7 | -26.5 | -0.8 |  |  |  |  |  |
|  | Foodcrust ext | EN | vivip 1 | 541.4 | SFA (C_10:0-28:0_), UFA (C_16:1, 18:1, 20:1, 22:1, 24:1_), DC (C_7-15_), Alk (C_17_), br, APAA (C_16, 18, 20_), chol, tmtd, phy, pri |  | 27.4 | 0.35 | -24.6 | -24.3 | 0.2 | 41.5 | -25.5 | 1.4 | 11.5 | 34.1 |
| Raku-930 | Ceramic | EN | vivip 1 | 3.2 | SFA (C_12:0-30:0_), UFA (C_16:1, 18:1, 22:1_), DC (C_8-14_), br, HAP, APAA (C_16, 18, 20_), chol, tmtd, phy, pri | b | 50.5 | 0.24 | -28.8 | -29.3 | -0.6 |  |  |  |  |  |
| Raku-931 | Ceramic | EN | vivip 1 | 8.4 | SFA (C_12:0-30:0_), UFA (C_16:1, 18:1, 22:1_), DC (C_9_) (tr), Alk (C_23, 27, 29_), br, terp, APAA (C_16, 18, 20_), chol, phyol, tmtd, phy, pri | d |  |  | -29.6 | -28.6 | 1.0 |  |  |  |  |  |
| Raku-932 | Ceramic | EN | 15-16 | 8.7 | SFA (C_13:0-30:0_), UFA (C_16:1, 18:1, 22:1_), DC (C_9-13_), Alk (C_29, 27_), br, APAA (C_16, 18, 20_), chol, phyol, tmtd, phy, pri | d | 42.0 |  | -25.7 | -27.1 | -1.3 |  |  |  |  |  |
| Raku-334 | Ceramic | EN | vivip 1 | 27.6 | SFA (C_12:0-30:0_), UFA (C_16:1, 18:1, 22:1_), DC (C_8-15_), 9-10diHFA (C_18_), br, HAP, APAA (C_16, 18, 20_), tmtd, phy, pri | f |  |  | -27.7 | -28.2 | -0.5 |  |  |  |  |  |
| Raku-933 | Ceramic | EN | 15-16 | 13.0 | SFA (C_12:0-30:0_), UFA (C_16:1, 18:1, 20:1, 22:1_), DC (C_9-13_), Alk (C_27_), br, terp, APAA (C_16, 18, 20_), chol, tmtd, phy, pri | c | 51.9 | 0.25 | -25.4 | -26.6 | -1.2 |  |  |  |  |  |
| Raku-935 | Ceramic | EN | 17-19 | 40.3 | SFA (C_12:0-30:0_), UFA (C_16:1, 18:1, 20:1, 22:1, 22:1_), DC (C_9-15, 18_), Alk (C_29_), br, terp, APAA (C_16, 18, 20_), chol, tmtd, phy, pri | d |  | 0.43 | -27.2 | -28.2 | -0.9 |  |  |  |  |  |

| Sample | Type | Phase | Layers | Lipid conc. (µg g^-1^) | Major compounds detected | volume | SRR  (%) | APAA C_20_/C_18_ | δ^13^C_16 :0_ (‰) | δ^13^C_18 :0_ (‰) | Δ^13^C  (C_18:0_-C_16:0_) | %C | δ^13^ C  (‰) | %N | δ^15^N  (‰) | C:N |
| --- | --- | --- | --- | --- | --- | --- | --- | --- | --- | --- | --- | --- | --- | --- | --- | --- |
| SedMB  01 | Sediment  (clay) | Excavation 3 area | | 10.3 | SFA (C_12:0-30:0_), UFA (C_18:1_), DC (C_16, 18-22_), br, Alk (C_25, 27, 29, 31_), phyol |  |  |  |  |  |  |  |  |  |  |  |
| SedMB  02 | Sediment | Excavation 3 area | | 0.7 | SFA (C_16:0-28:0_), UFA (C_18:1_), DC (C_16, 18-22_), Alk (C_27_) (tr) |  |  |  |  |  |  |  |  |  |  |  |
| SedMB  03 | Sediment | Excavation 3 area | | 15.8 | SFA (C_14:0-30:0_), UFA (C_16:1, 18:1_), DC (C_18-25_), br, Alk (C_27, 29_), Triterp, phyol |  |  |  |  |  |  |  |  |  |  |  |
| SedMB  04 | Sediment (ash) | Excavation 3 area | | 29.1 | SFA (C_14:0-30:0_), UFA (C_16:1, 18:1_), DC (C_16, 18, 20, 22_), br, Alk (C_27, 29_), phyol |  |  |  |  |  |  |  |  |  |  |  |
| SedMB  05 | Sediment | Excavation 1/4 area | | 0.3 | SFA (C_16:0-28:0_), UFA (C_18:1, 22:1_) |  |  |  |  |  |  |  |  |  |  |  |
| SedMB  06 | Sediment | Excavation 1/4 area | | 37.6 | SFA (C_14:0-30:0_), UFA (C_16:1, 18:1_), DC (C_19, 30_), br, Alk (C_27, 29_), Triterp, phyol |  |  |  |  |  |  |  |  |  |  |  |
| SedMB  07 | Sediment | Excavation 1/4 area | | 2.4 | SFA (C_14:0-30:0_), UFA (_18:1_) (tr), br, Alk (C_23-25, 27, 29_) |  |  |  |  |  |  |  |  |  |  |  |
| SedMB  08 | Sediment | Excavation 1/4 area | | 0.8 | SFA (C_15:0-30:0_), br |  |  |  |  |  |  |  |  |  |  |  |

*Sherds, internal (int) and external (ext) foodcrusts were all analysed by acid-methanol extraction and a selection of samples by solvent extraction and trimethylsilylated ^1^ - carboxilic acids (Cn:x) with carbon length n and number of unsaturations x, SFA – saturated fatty acid, UFA – unsaturated fatty acids, DC - α,ω-dicarboxylic acids, Alk – alkane, Alkone – alkanone, PAH – polyaromatic hydrocarbons, APAA - ω-(o-alkylphenyl) alkanoic acids, br -branched chain acids dominated by iso and anteiso C_15_ and C_17_, tmtd - 4,8,12-trimethyltridecanoic acid, pri – pristanic acid, phy – phytanic acid with the percentage contribution of SRR diastereomer in total phytanic acid, chol - cholesterol or derivatives, phyol - phytosterol or derivatives, Copr – coprostanol, abie – abietic acid, terp – terpenes (mainly methyl-dehydroabietic acid and 7-oxo- dehydroabietic acid), terp* – presence of retene, Triterp – triterpenes and samples from EN – Early Neolithic. Volumes of RY pottery correspond to (a) < 0.5 L, (b) 0.5 L, (c) 1-2 L, (d) 2-3 L, (e) 5-6 L and (f) 15-20 L.*

**Table (S2):** **Stable carbon isotopes** **of n-hexadecanoic (C_16:0_) and n-octadecanoic (C_18:0_) acid of reference fats from modern animal products. Data are from different studies, with additional new freshwater fish and shellfish, ruminants and terrestrial plants from Rakushechny Yar region or Western Russia.**

| Common name | Taxonomic name | Provenience | δ^13^C_16:0_ (‰) | δ^13^C_18:0_ (‰) | Δ^13^C  (C_18:0_- C_16:0_) | Reference |
| --- | --- | --- | --- | --- | --- | --- |
| *Freshwater fish* | | | | | | |
| Pike | *Esox lucius* | Denmark | -34.8 | -35.0 | -0.2 | Craig et al. 2011 |
| Tench | *Tinca tinca* | Denmark | -27.7 | -28.8 | -1.1 | Craig et al. 2011 |
| Tench | *Tinca tinca* | Denmark | -24.2 | -26.3 | -2.1 | Craig et al. 2011 |
| Tench | *Tinca tinca* | Denmark | -37.2 | -36.5 | 0.7 | Craig et al. 2011 |
| Freshwater fish | n/a | Kazakhstan | -32.1 | -32.2 | 0.0 | Outram et al. 2009 |
| Freshwater fish | n/a | Kazakhstan | -32.1 | -31.5 | 0.7 | Outram et al. 2009 |
| Freshwater fish | n/a | Kazakhstan | -31.8 | -31.9 | -0.1 | Outram et al. 2009 |
| Freshwater fish | n/a | Kazakhstan | -31.7 | -31.4 | 0.3 | Outram et al. 2009 |
| Freshwater fish | n/a | Kazakhstan | -31.5 | -31.4 | 0.1 | Outram et al. 2009 |
| Freshwater fish | n/a | Kazakhstan | -31.6 | -29.9 | 1.7 | Outram et al. 2009 |
| Freshwater fish | n/a | Kazakhstan | -31.1 | -30.9 | 0.2 | Outram et al. 2009 |
| Freshwater fish | n/a | Kazakhstan | -30.9 | -30.4 | 0.5 | Outram et al. 2009 |
| Freshwater fish | n/a | Kazakhstan | -30.7 | -30.2 | 0.5 | Outram et al. 2009 |
| Freshwater fish | n/a | Kazakhstan | -30.6 | -30.6 | 0.0 | Outram et al. 2009 |
| Common name | Taxonomic name | Provenience | δ^13^C_16:0_ (‰) | δ^13^C_18:0_ (‰) | Δ^13^C  (C_18:0_- C_16:0_) | Reference |
| Bleak | *Alburnus alburnus* | Finland | -26.6 | -25.9 | 0.7 | Pääkkönen et al. 2016 |
| Bleak | *Alburnus alburnus* | Finland | -33.8 | -32.3 | 1.5 | Pääkkönen et al. 2016 |
| Bleak | *Alburnus alburnus* | Finland | -34.1 | -34.5 | -0.4 | Pääkkönen et al. 2016 |
| Bleak | *Alburnus alburnus* | Finland | -35.6 | -35.1 | 0.5 | Pääkkönen et al. 2016 |
| Bleak | *Alburnus alburnus* | Finland | -36.0 | -34.5 | 1.5 | Pääkkönen et al. 2016 |
| Burbot | *Lota lota* | Finland | -34.3 | -32.3 | 2.0 | Pääkkönen et al. 2016 |
| Ide | *Leuciscus idus* | Finland | -33.4 | -31.8 | 1.6 | Pääkkönen et al. 2016 |
| Ide | *Leuciscus idus* | Finland | -32.8 | -31.2 | 1.6 | Pääkkönen et al. 2016 |
| Northern pike | *Esox lucius* | Finland | -33.2 | -32.9 | 0.3 | Pääkkönen et al. 2016 |
| Northern pike | *Esox lucius* | Finland | -33.4 | -32.2 | 1.2 | Pääkkönen et al. 2016 |
| Northern pike | *Esox lucius* | Finland | -31.7 | -31.3 | 0.4 | Pääkkönen et al. 2016 |
| Perch | *Perca fluviatilis* | Finland | -34.3 | -33.2 | 1.1 | Pääkkönen et al. 2016 |
| Perch | *Perca fluviatilis* | Finland | -35.1 | -34.6 | 0.5 | Pääkkönen et al. 2016 |
| Perch | *Perca fluviatilis* | Finland | -33.3 | -32.5 | 0.8 | Pääkkönen et al. 2016 |
| Perch | *Perca fluviatilis* | Finland | -34.9 | -33.3 | 1.6 | Pääkkönen et al. 2016 |
| Perch | *Perca fluviatilis* | Finland | -32.9 | -32.3 | 0.6 | Pääkkönen et al. 2016 |
| Perch | *Perca fluviatilis* | Finland | -32.0 | -32.3 | -0.3 | Pääkkönen et al. 2016 |
| Common name | Taxonomic name | Provenience | δ^13^C_16:0_ (‰) | δ^13^C_18:0_ (‰) | Δ^13^C  (C_18:0_- C_16:0_) | Reference |
| Perch | *Perca fluviatilis* | Finland | -32.0 | -32.6 | -0.6 | Pääkkönen et al. 2016 |
| Perch | *Perca fluviatilis* | Finland | -35.3 | -36.8 | -1.5 | Pääkkönen et al. 2016 |
| Perch | *Perca fluviatilis* | Finland | -35.2 | -34.7 | 0.5 | Pääkkönen et al. 2016 |
| Pikeperch | *Sander lucioperca* | Finland | -34.5 | -33.7 | 0.8 | Pääkkönen et al. 2016 |
| Pikeperch | *Sander lucioperca* | Finland | -30.2 | -29.9 | 0.3 | Pääkkönen et al. 2016 |
| Roach | *Rutilus rutilus* | Finland | -29.4 | -31.9 | -2.5 | Pääkkönen et al. 2016 |
| Roach | *Rutilus rutilus* | Finland | -34.0 | -32.9 | 1.1 | Pääkkönen et al. 2016 |
| Arctic grayling | *Thymallus arcticus* | Alaska | -40.9 | -39.0 | 1.9 | Choy et al 2016 |
| Burbot | *Lota lota* | Alaska | -26.9 | -28.2 | -1.3 | Choy et al 2016 |
| Burbot | *Lota lota* | Alaska | -29.8 | -28.8 | 1.0 | Choy et al 2016 |
| Northern pike | *Esox lucius* | Alaska | -32.9 | -30.7 | 2.2 | Choy et al 2016 |
| Northern pike | *Esox lucius* | Alaska | -35.8 | -35.6 | 0.2 | Choy et al 2016 |
| Northern pike | *Esox lucius* | Alaska | -36.0 | -35.0 | 1.0 | Choy et al 2016 |
| Sheefish | *Stenodus nelma* | Alaska | -34.0 | -34.4 | -0.4 | Choy et al 2016 |
| Bering cisco | *Coregonus laurettae* | Alaska | -34.6 | -34.3 | 0.3 | Choy et al 2016 |
| Northern pike | *Esox lucius* | Russia - Middle Don | -36.4 | -35.5 | 1.0 | This study |
| Roach | *Rutilus rutilus* | Russia - Middle Don | -36.3 | -34.7 | 1.7 | This study |
| Common name | Taxonomic name | Provenience | δ^13^C_16:0_ (‰) | δ^13^C_18:0_ (‰) | Δ^13^C  (C_18:0_- C_16:0_) | Reference |
| Crucian carp | *Carassius carassius* | Russia - Middle Don | -35.0 | -34.2 | 0.8 | This study |
| European perch | *Perca fluviatilis* | Russia - Lower Don | -32.1 | -29.9 | 2.2 | This study |
| Ide | *Leuciscus idus* | Russia - Syktyvkar | -29.3 | -29.0 | 0.3 | This study |
| Cisco | *n/a* | Russia - Syktyvkar | -35.5 | -35.5 | 0.0 | This study |
| Grayling | *Thymallus thymallus* | Russia - Syktyvkar | -34.4 | -34.1 | 0.3 | This study |
| Northern pike | *Esox lucius* | Russia - Syktyvkar | -35.0 | -34.4 | 0.6 | This study |
| Wels catfish | *Silurus glanis* | Russia-Lower Don | -27.7 | -27.8 | -0.1 | This study |
| *Freshwater shell* | | | | | | |
| Mussel | *Unio* | Russia-Lower Don | -32.6 | -31.8 | 0.7 | This study |
| Mussel | *Unio* | Russia-Lower Don | -34.2 | -33.5 | 0.8 | This study |
| Mussel | *Unio* | Russia-Lower Don | -34.2 | -33.1 | 1.1 | This study |
| Mussel | *Unio* | Russia-Lower Don | -31.6 | -31.2 | 0.4 | This study |
| Mussel | *Unio* | Russia-Lower Don | -34.2 | -33.0 | 1.2 | This study |
| Mussel | *Unio* | Russia-Lower Don | -35.2 | -34.1 | 1.1 | This study |
| Mussel | *Unio* | Russia-Lower Don | -33.8 | -32.2 | 1.5 | This study |
| Mussel | *Unio* | Russia-Lower Don | -32.2 | -30.2 | 2.0 | This study |
| Mussel | *Unio* | Russia-Lower Don | -33.8 | -32.8 | 1.0 | This study |
| Common name | Taxonomic name | Provenience | δ^13^C_16:0_ (‰) | δ^13^C_18:0_ (‰) | Δ^13^C  (C_18:0_- C_16:0_) | Reference |
| Mussel | *Unio* | Russia-Lower Don | -33.0 | -31.2 | 1.9 | This study |
| Snail | *Viviparus diluvianus* | Russia-Lower Don | -34.3 | -33.3 | 1.0 | This study |
| Snail | *Viviparus diluvianus* | Russia-Lower Don | -34.0 | -33.9 | 0.1 | This study |
| Snail | *Viviparus diluvianus* | Russia-Lower Don | -34.5 | -33.1 | 1.4 | This study |
| Snail | *Viviparus diluvianus* | Russia-Lower Don | -33.7 | -32.9 | 0.8 | This study |
| Snail | *Viviparus diluvianus* | Russia-Lower Don | -33.4 | -33.1 | 0.3 | This study |
| Snail | *Viviparus diluvianus* | Russia-Lower Don | -32.8 | -33.2 | -0.4 | This study |
| Snail | *Viviparus diluvianus* | Russia-Lower Don | -34.0 | -32.7 | 1.3 | This study |
| Snail | *Viviparus diluvianus* | Russia-Lower Don | -33.5 | -33.2 | 0.3 | This study |
| Snail | *Viviparus diluvianus* | Russia-Lower Don | -34.0 | -33.7 | 0.3 | This study |
| Snail | *Viviparus diluvianus* | Russia-Lower Don | -33.0 | -32.5 | 0.5 | This study |
| *Migratory fish* | | | | | | |
| Salmon | *Salmonidae* | Japan | -27.3 | -28.0 | -0.7 | Craig et al. 2013 |
| Salmon | *Salmonidae* | Japan | -24.5 | -26.1 | -1.6 | Craig et al. 2013 |
| Salmon | *Salmonidae* | Japan | -25.3 | -26.6 | -1.3 | Craig et al. 2013 |
| Trout | *Salmonidae* | Japan | -26.0 | -25.9 | 0.0 | Craig et al. 2013 |
| Trout | *Salmonidae* | Japan | -26.8 | -27.0 | -0.2 | Craig et al. 2013 |
| Common name | Taxonomic name | Provenience | δ^13^C_16:0_ (‰) | δ^13^C_18:0_ (‰) | Δ^13^C  (C_18:0_- C_16:0_) | Reference |
| Trout | *Salmonidae* | Japan | -25.1 | -25.5 | -0.4 | Craig et al. 2013 |
| Trout | *Salmonidae* | Japan | -24.1 | -22.4 | 1.7 | Lucquin et al 2016a |
| Salmon | *Salmonidae* | Japan | -25.8 | -24.3 | 1.4 | Lucquin et al 2016a |
| Trout | *Salmonidae* | Japan | -23.3 | -23.7 | -0.4 | Lucquin et al 2016a |
| Salmon | *Salmonidae* | Japan | -27.9 | -28.4 | -0.5 | Lucquin et al 2016a |
| Cherry Salmon | *Salmonidae* | Japan | -24.8 | -23.8 | 1.0 | Lucquin et al 2016a |
| Coast Salmon | *Salmonidae* | Japan | -23.7 | -22.6 | 1.1 | Lucquin et al 2016a |
| Salmon | *Salmonidae* | Japan | -25.8 | -24.8 | 1.0 | Lucquin et al 2016a |
| Atlantic salmon | *Salmo salar* | Finland | -25.0 | -23.6 | 1.4 | Pääkkönen et al. 2016 |
| Atlantic salmon | *Salmo salar* | Finland | -24.3 | -23.9 | 0.4 | Pääkkönen et al. 2016 |
| Coho salmon | *Oncorhynchus kisutch* | Alaska | -28.2 | -26.6 | 1.6 | Choy et al 2016 |
| Coho salmon | *Oncorhynchus kisutch* | Alaska | -27.8 | -26.0 | 1.8 | Choy et al 2016 |
| Coho salmon | *Oncorhynchus kisutch* | Alaska | -27.2 | -25.0 | 2.2 | Choy et al 2016 |
| Chum salmon | *Oncorhynchus keta* | Alaska | -26.2 | -25.4 | 0.8 | Choy et al 2016 |
| Chum salmon | *Oncorhynchus keta* | Alaska | -26.2 | -24.8 | 1.4 | Choy et al 2016 |
| Chum salmon | *Oncorhynchus keta* | Alaska | -25.1 | -23.5 | 1.6 | Choy et al 2016 |
| Danube sturgeon | *Acipenser gueldenstaedtii* | Russia-Volga | -26.0 | -26.2 | -0.2 | This study |
| Common name | Taxonomic name | Provenience | δ^13^C_16:0_ (‰) | δ^13^C_18:0_ (‰) | Δ^13^C  (C_18:0_- C_16:0_) | Reference |
| *Wild ruminant* | | | | | | |
| Red deer | *Cervus elaphus* | Poland | -27.7 | -31.5 | -3.8 | Craig et al. 2012 |
| Red deer | *Cervus elaphus* | Poland | -27.4 | -31.1 | -3.7 | Craig et al. 2012 |
| Red deer | *Cervus elaphus* | Poland | -28.4 | -32.6 | -4.2 | Craig et al. 2012 |
| Red deer | *Cervus elaphus* | Poland | -30.0 | -33.7 | -3.7 | Craig et al. 2012 |
| Red deer | *Cervus elaphus* | Poland | -29.1 | -32.7 | -3.6 | Craig et al. 2012 |
| Red deer | *Cervus elaphus* | Poland | -28.8 | -33.0 | -4.2 | Craig et al. 2012 |
| Red deer | *Cervus elaphus* | Poland | -30.4 | -33.0 | -2.6 | Craig et al. 2012 |
| Red deer | *Cervus elaphus* | Poland | -29.5 | -33.1 | -3.6 | Craig et al. 2012 |
| Red deer | *Cervus elaphus* | Poland | -28.9 | -32.3 | -3.4 | Craig et al. 2012 |
| Red deer | *Cervus elaphus* | Poland | -29.4 | -33.0 | -3.6 | Craig et al. 2012 |
| Red deer | *Cervus elaphus* | Poland | -31.0 | -33.1 | -2.2 | Spangenberg et al. 2006 |
| Moose | *Alces alces* | Alaska | -30.8 | -31.9 | -1.1 | Choy et al 2016 |
| Roe deer | *Capreolus capreolus* | Russia - Middle Don | -31.1 | -32.4 | -1.3 | This study |
| Deer | *n/a* | Russia - Middle Don | -28.7 | -32.0 | -3.3 | This study |
| Reindeer | *Rangifer tarandus* | Russia - Syktyvkar | -23.8 | -24.9 | -1.1 | This study |
| Elk | *Alces alces* | Russia - Upper Volga | -30.5 | -32.0 | -1.5 | This study |
| Common name | Taxonomic name | Provenience | δ^13^C_16:0_ (‰) | δ^13^C_18:0_ (‰) | Δ^13^C  (C_18:0_- C_16:0_) | Reference |
| Elk | *Alces alces* | Russia - Upper Volga | -29.8 | -31.0 | -1.1 | This study |
| Elk | *Alces alces* | Russia - Syktyvkar | -33.2 | -34.8 | -1.6 | This study |
| *Plants* | | | | | | |
| Bulrush | *Typha* | Russia-Lower Don | -31.79 | -33.1 | -1.3 | This study |
| Wild thyme | *Thymus* | Russia-Lower Don | -38.51 | -34.8 | 3.7 | This study |
| Silverberry | *Elaeagnus* | Russia-Lower Don | -31.93 | -32.0 | -0.0 | This study |
| Wild rose berry | *Rosa* | Russia-Lower Don | -28.98 | -29.7 | -0.7 | This study |
| Dog rose berry | *Rosa canina* | Russia-Lower Don | -32.12 | -32.7 | -0.6 | This study |
| Wild pear | *Pyrus* | Russia-Lower Don | -33.53 | -32.4 | 1.2 | This study |
| Wild apple | *Malus* | Russia-Lower Don | -32.7 | -30.1 | 2.6 | This study |
| *Ruminant dairy* | | | | | | |
| Cow | *Bos taurus* | United Kingdom | -30.8 | -34.4 | -3.6 | Dudd 1999 |
| Cow | *Bos taurus* | United Kingdom | -27.8 | -32.1 | -4.3 | Dudd 1999 |
| Sheep | *Ovis aries* | United Kingdom | -29.4 | -33.8 | -4.4 | Dudd 1999 |
| Sheep | *Ovis aries* | United Kingdom | -29 | -33.4 | -4.4 | Dudd 1999 |
| Cow | *Bos taurus* | United Kingdom | -27.4 | -32.2 | -4.8 | Dudd 1999 |
| Cow | *Bos taurus* | United Kingdom | -28.9 | -33.7 | -4.8 | Dudd 1999 |
| Common name | Taxonomic name | Provenience | δ^13^C_16:0_ (‰) | δ^13^C_18:0_ (‰) | Δ^13^C  (C_18:0_- C_16:0_) | Reference |
| Cow | *Bos taurus* | United Kingdom | -29.6 | -34.9 | -5.3 | Dudd 1999 |
| Cow | *Bos taurus* | United Kingdom | -27.9 | -33.1 | -5.2 | Dudd 1999 |
| Cow | *Bos taurus* | United Kingdom | -28.6 | -34.1 | -5.5 | Dudd 1999 |
| Cow | *Bos taurus* | United Kingdom | -28.1 | -34 | -5.9 | Dudd 1999 |
| *Non-ruminant* | | | | | | |
| Beaver | *Castor fiber* | Estonia | -31.4 | -32.1 | -0.7 | Courel et al. 2020 |
| Beaver | *Castor fiber* | Estonia | -31.3 | -32.1 | -0.8 | Courel et al. 2020 |
| Beaver | *Castor fiber* | Estonia | -31.2 | -31.7 | -0.5 | Courel et al. 2020 |
| Beaver | *Castor fiber* | Estonia | -31.0 | -31.6 | -0.6 | Courel et al. 2020 |
| Beaver | *Castor fiber* | Finland | -29.9 | -31.0 | -1.1 | Pääkkönen et al. 2020 |
| Beaver | *Castor fiber* | Russia - Upper Volga | -30.8 | -31.0 | -0.2 | This study |
| Beaver | *Castor fiber* | Estonia | -31.0 | -30.8 | 0.2 | Courel et al. 2020 |
| Beaver | *Castor canadensis* | Canada | -30.1 | -30.3 | -0.2 | Taché and Craig 2015 |
| Beaver | *Castor fiber* | Estonia | -30.0 | -30.2 | -0.2 | Courel et al. 2020 |
| Beaver | *Castor fiber* | Estonia | -29.9 | -30.0 | -0.1 | Courel et al. 2020 |
| Beaver | *Castor fiber* | Estonia | -29.3 | -29.7 | -0.4 | Courel et al. 2020 |
| Beaver | *Castor fiber* | Finland | -27.7 | -28.9 | -1.2 | Pääkkönen et al. 2020 |
| Common name | Taxonomic name | Provenience | δ^13^C_16:0_ (‰) | δ^13^C_18:0_ (‰) | Δ^13^C  (C_18:0_- C_16:0_) | Reference |
| Beaver | *Castor fiber* | Finland | -28.3 | -28.6 | -0.3 | Pääkkönen et al. 2020 |
| Beaver | *Castor fiber* | Russia - Middle Don | -31.1 | -28.5 | 2.6 | This study |
| Horse | *Equus caballus* | n/a | -29.7 | -29.4 | 0.3 | Dudd 1999 |
| Horse | *Equus caballus* | n/a | -30.6 | -30.1 | 0.5 | Dudd 1999 |
| Horse | *Equus caballus* | n/a | -30.5 | -29.6 | 0.9 | Dudd 1999 |
| Horse | *Equus caballus* | n/a | -30.0 | -29.2 | 0.8 | Dudd 1999 |
| Horse | *Equus caballus* | n/a | -30.3 | -29.9 | 0.4 | Dudd 1999 |
| Horse | *Equus caballus* | n/a | -29.5 | -29.6 | -0.1 | Dudd 1999 |
| Horse | *Equus caballus* | n/a | -29.6 | -27.5 | 2.1 | Dudd 1999 |
| Horse | *Equus caballus* | n/a | -29.9 | -29.7 | 0.2 | Dudd 1999 |
| Horse | *Equus caballus* | Kazakhstan | -28.2 | -27.9 | 0.3 | Outram et al. 2009 |
| Horse | *Equus caballus* | Kazakhstan | -28.2 | -28.5 | -0.3 | Outram et al. 2009 |
| Horse | *Equus caballus* | Kazakhstan | -27.7 | -28.6 | -0.9 | Outram et al. 2009 |
| Horse | *Equus caballus* | Kazakhstan | -25.9 | -27.0 | -1.1 | Outram et al. 2009 |
| Horse | *Equus caballus* | Kazakhstan | -25.6 | -26.0 | -0.4 | Outram et al. 2009 |
| Wild boar | *Sus scrofa* | Japan | -27.3 | -26.4 | 0.9 | Lucquin et al. 2016b |
| Wild boar | *Sus scrofa* | Japan | -27.7 | -26.7 | 1.0 | Lucquin et al. 2016b |
| Common name | Taxonomic name | Provenience | δ^13^C_16:0_ (‰) | δ^13^C_18:0_ (‰) | Δ^13^C  (C_18:0_- C_16:0_) | Reference |
| Wild boar | *Sus scrofa* | Japan | -27.6 | -26.9 | 0.7 | Lucquin et al. 2016b |
| Wild boar | *Sus scrofa* | Japan | -27.4 | -26.3 | 1.1 | Horiuchi et al. 2015 |
| Wild boar | *Sus scrofa* | Japan | -27.5 | -26.2 | 1.3 | Horiuchi et al. 2015 |
| Wild boar | *Sus scrofa* | Japan | -25.5 | -26.0 | -0.5 | Horiuchi et al. 2015 |
| Wild boar | *Sus scrofa* | Japan | -27.2 | -26.5 | 0.7 | Horiuchi et al. 2015 |

*The δ^13^C values of the modern references were adjusted for the addition of the effects of post-industrial carbon in order to facilitate the comparison with the archaeological samples* [*(Hellevang and Aagaard 2015)*](https://paperpile.com/c/wuLnGy/eEsm+BBR7+ueXW)*.*

**Table (S3): Bulk isotope measurement of archaeological bone collagen from Rakushechny Yar site.**

| **Common** **name** | **Taxonomic name** | **Collagen yield (%)** | **%C** | **δ^13^C (Collagen)**  **(‰)** | **%N** | **δ^15^N (collagen)**  **(‰)** | **C:N** | **^14^C lab numbers** |
| --- | --- | --- | --- | --- | --- | --- | --- | --- |
| ***Freshwater fish*** | | | | | | | | |
| Zander | *Sander lucioperca* | 3.2 | 37.4 | -24.1 | 13.6 | 12.3 | 3.2 |  |
| Zander | *Sander lucioperca* | 2.2 | 38.6 | -16.5 | 13.9 | 12.9 | 3.2 |  |
| Zander | *Sander lucioperca* | 5.8 | 42.2 | -21.7 | 15.0 | 10.4 | 3.3 |  |
| Zander | *Sander lucioperca* | 5.5 | 43.0 | -21.0 | 15.8 | 11.9 | 3.2 |  |
| Wels catfish | *Silurus glanis* | 4.5 | 41.9 | -24.0 | 14.4 | 11.2 | 3.4 |  |
| ***Migratory fish*** | | | | | | | | |
| Sturgeon | *Acipenser sp.* | 1.0 |  | -23.8 |  | 12.5 | 2.9 | P48885 |
| Sturgeon | Acipenser sp. | 1.3 |  | -15.9 |  | 12.4 | 2.9 | P48886 |
| Sturgeon | Acipenser sp. | 0.5 |  | -15.4 |  | 14.0 | 3.0 | P48887 |
| Sturgeon | Acipenser sp. | 0.6 |  | -16.8 |  | 14.5 | 3.0 | P48888 |
| Sturgeon | Acipenser sp. | 1.5 |  | -15.1 |  | 13.0 | 3.2 | P48889 |
| Sturgeon | Acipenser sp. | 1.8 |  | -17.3 |  | 13.2 | 3.2 | P48890 |
| Sturgeon | Acipenser sp. | 1.8 |  | -17.0 |  | 13.1 | 3.2 | P48891 |
| ***Wild ruminant*** | | | | | | | | |
| Red deer | *Cervus elaphus* | 14.3 | 41.6 | -20.2 | 15.1 | 6.2 | 3.2 |  |
| Red deer | *Cervus elaphus* | 13.2 | 42.5 | -19.8 | 15.5 | 6.0 | 3.2 |  |
| Red deer | *Cervus elaphus* | 3.1 | - | -20.4 | - | 7.0 | 3.2 | OxA-39362 |
| Red deer | *Cervus elaphus* | 4.5 | - | -20.4 | - | 7.7 | 3.2 | SUERC-86132 |
| Red deer | *Cervus elaphus* | 8.9 | 42.1 | -19.9 | 15.5 | 6.2 | 3.2 |  |
| Red deer | *Cervus elaphus* | 16.7 | 43.3 | -19.2 | 15.9 | 6.5 | 3.2 |  |
| Red deer | *Cervus elaphus* | 3.6 | 43.5 | -20.2 | 16.0 | 5.1 | 3.2 |  |
| Red deer | *Cervus elaphus* | 16.0 | 43.4 | -19.8 | 15.9 | 5.9 | 3.2 |  |
| Red deer | *Cervus elaphus* | 11.8 | 42.9 | -19.9 | 15.9 | 5.4 | 3.1 |  |
| Red deer | *Cervus elaphus* | 11.5 | 43.2 | -19.5 | 15.7 | 5.4 | 3.2 |  |
| **Common** **name** | **Taxonomic name** | **Collagen yield (%)** | **%C** | **δ^13^C (Collagen)**  **(‰)** | **%N** | **δ^15^N (Collagen)**  **(‰)** | **C:N** | **^14^C lab numbers** |
| Red deer | *Cervus elaphus* | 13.6 | 43.6 | -19.6 | 16.0 | 6.0 | 3.2 |  |
| Red deer | *Cervus elaphus* | 13.6 | 40.1 | -20.0 | 15.2 | 6.0 | 3.1 |  |
| Red deer | *Cervus elaphus* | 9.0 | 42.9 | -20.1 | 15.8 | 5.5 | 3.2 |  |
| Red deer | *Cervus elaphus* | 17.4 | 42.5 | -19.6 | 15.8 | 5.4 | 3.1 |  |
| Red deer | *Cervus elaphus* | 4.4 | 41.6 | -21.0 | 15.3 | 5.9 | 3.2 |  |
| Red deer | *Cervus elaphus* | 10.2 | - | -20.2 | - | 7.6 | 3.2 | SUERC-86131 |
| Red deer | *Cervus elaphus* | 9.5 | - | -19.9 | - | 6.6 | 3.2 | SUERC-86130 |
| Red deer | *Cervus elaphus* | 8.20 | 43.15 | -20.0 | 16.0 | 5.1 | 3.2 |  |
| Red deer | *Cervus elaphus* | 2.7 | 39.8 | -19.8 | 15.0 | 5.2 | 3.1 |  |
| Red deer | *Cervus elaphus* | 3.0 | - | -21.0 | - | 6.6 | 3.2 | SUERC-86130 |
| Roe deer | *Capreolus capreolus* | 2.3 | - | -21.2 | - | 6.7 | 3.2 | OxA-39364 |
| Roe deer | *Capreolus capreolus* | - | - | -20.1 | - | 7.3 | 3.2 |  |
| Deer | *-* | 5.0 | - | -20.3 | - | 9.4 | 3.2 | OxA-39361 |
| Deer | *-* | 8.0 | - | -20.8 | - | 5.3 | 3.2 | OxA-39363 |
| ***Non Ruminant*** |  |  |  |  |  |  |  |  |
| Pig | *Suidae* | 8.4 | 44.2 | -20.9 | 16.1 | 7.9 | 3.2 |  |
| Pig | *Suidae* | 11.6 | 41.2 | -20.8 | 15.1 | 8.4 | 3.2 |  |
| Pig | *Suidae* | 15.4 | 42.9 | -20.7 | 15.9 | 7.9 | 3.2 |  |
| Pig | *Suidae* | 7.9 | 42.1 | -20.5 | 15.3 | 7.3 | 3.2 |  |
| Pig | *Suidae* | 12.4 | - | -20.1 | - | 9.9 | 3.2 | SUERC-86129 |
| Pig | *Suidae* | 2.5 | - | -20.5 | - | 8.9 | 3.2 | OxA-39360 |
| Pig | *Suidae* | 2.6 | - | -20.5 | - | 9.1 | 3.3 | SUERC-86128 |
| Beaver | *Castor fiber* | 14.3 | 41.6 | -20.2 | 15.1 | 6.2 | 3.2 |  |
| Horse | *Equus ferus* | 16.0 | - | -19.7 |  | 5.0 | 3.2 | SUERC-88043 |
| Horse | *Equus ferus* | 2.4 | - | -21.1 | - | 5.5 | 3.2 | SUERC-86137 |
| Horse | *Equus ferus* | 11.2 | - | -20.3 | - | 5.9 | 3.1 | SUERC-86136 |

*For the samples used for radiocarbon dating, %C and %N were measured but not reported.*

**Table (S4):** **SRR% values calculated from beaver tissues, either raw or heated, extracted within the scope of this study or in previous studies (Courel et al. 2020 and Bondetti et al., submitted).**

| Common name | Taxonomic name | Sample type | Provenience | SRR% | Reference |
| --- | --- | --- | --- | --- | --- |
| Beaver | *Castor fiber* | Raw tissue | Estonia | 99.6 | Courel et al. 2020 |
| Beaver | *Castor fiber* | Raw tissue | Estonia | 99.5 | Courel et al. 2020 |
| Beaver | *Castor fiber* | Raw tissue | Estonia | 99.7 | Courel et al. 2020 |
| Beaver | *Castor fiber* | Raw tissue | Estonia | 99.6 | Courel et al. 2020 |
| Beaver | *Castor fiber* | Raw tissue | Estonia | 99.4 | Courel et al. 2020 |
| Beaver | *Castor fiber* | Raw tissue | Estonia | 97.7 | Courel et al. 2020 |
| Beaver | *Castor fiber* | Raw tissue | Estonia | 99.9 | Courel et al. 2020 |
| Beaver | *Castor fiber* | Heated tissue | Estonia | 90.5 | Bondetti et al. 2020a |
| Beaver | *Castor fiber* | Raw tissue | Russia - Middle Don | 99.7 | This study |
| Beaver | *Castor fiber* | Raw tissue | Russia - Upper Volga | 99.7 | This study |

**Table (S5): List of samples selected for ZooMS analysis and the results.**

| Sample ID | Zooarchaeological identification | Layer | P1 | A1 | A2 | B | C | P2 | D | E | F1 | F2 | G1 | G2 | ZooMS identification |
| --- | --- | --- | --- | --- | --- | --- | --- | --- | --- | --- | --- | --- | --- | --- | --- |
| I14C0005 | large mammal | pit | 1105.6 | - | - | 1453.7 | - | - | 2131.1 | 2820.4 | - | - | - | - | Pig^2^ |
| I14C0006 | Sheep | 15a | 1105.6 | 1180.6 | 1196.6 | 1427.7 | **1550.8** | 1648.8 | 2131.1 | - | 2883.4 | 2899.4 | 3017.5 | 3033.5 | Red deer^1^ |
| I14C0010 | large mammal | 15a | 1105.6 | 1180.6 | 1196.6 | 1427.7 | 1550.8 | 1648.8 | 2131.1 | 2792.3 | 2883.4 | 2899.4 | 3017.5 | 3033.5 | Red deer^1^ |
| I14C0011 | n/a | 16 | 1105.6 | - | - | 1427.7 | 1550.8 | 1648.8 | 2131.1 |  | 2883.4 | 2899.4 | 3017.5 | 3033.5 | Red deer^1^ |
| I14C0012 | n/a | 16 | 1105.6 | 1180.6 |  | 1427.7 | 1550.8 | 1648.8 | 2131.1 | 2792.3 | 2883.4 | 2899.4 | 3017.5 | 3033.5 | Red deer^1^ |
| I14C0013 | medium mammal | 16 | 1105.6 | - | - | 1427.7 | 1550.8 | 1648.8 | 2131.1 | 2792.3 | 2883.4 | 2899.4 | 3017.5 | 3033.5 | Red deer^1^ |
| I14C0014 | medium mammal | 16 | - | - | - | 1453.7 | - | - | 2131.1 | 2820.4 | 2883.4 | - | 3017.5 | 3033.5 | Pig^2^ |
| I14C0015 | large mammal | 17 | 1105.6 | 1180.6 | 1196.6 | 1427.7 | 1550.8 | 1648.8 | 2131.1 | 2792.3 | 2883.4 | 2899.4 | 3017.5 | 3033.5 | Red deer^1^ |
| I14C0016 | large mammal | 17 | 1105.6 | - | - | 1427.7 | 1550.8 | 1648.8 | 2131.1 | 2792.3 | 2883.4 | 2899.4 | 3017.5 | 3033.5 | Red deer^1^ |
| I14C0017 | n/a | 17 | 1105.6 | - | - | 1427.7 | 1550.8 | 1648.8 | 2131.1 | 2792.3 | 2883.4 | 2899.4 | 3017.5 | 3033.5 | Red deer^1^ |
| I14C0018 | Sheep | 17 | 1105.6 | - | - | 1427.7 | **1550.8** | 1648.8 | 2131.1 | 2792.3 | 2883.4 | 2899.4 | 3017.5 | 3033.5 | Red deer^1^ |
| I14C0019 | large mammal | 17 | 1105.6 | - | - | 1427.7 | 1550.8 | 1648.8 | 2131.1 | 2792.3 | 2883.4 | 2899.4 | 3017.5 | 3033.5 | Red deer^1^ |
| I14C0021 | large mammal | 17 | 1105.6 | - | - | 1427.7 | 1550.8 | 1648.8 | 2131.1 | 2792.3 | 2883.4 | 2899.4 | 3017.5 | 3033.5 | Red deer^1^ |
| I14C0023 | medium mammal | 17 | 1105.6 | 1180.6 | 1196.6 | 1427.7 | 1550.8 | 1648.8 | 2131.1 | 2792.3 | 2883.4 | 2899.4 | 3017.5 | - | Red deer^1^ |
| I14C0024 | large mammal | 17 | 1105.6 | - | - | 1427.7 | 1550.8 | 1648.8 | 2131.1 | - | 2883.4 | 2899.4 | - | - | Red deer^1^ |
| I14C0063 | n/a | upper vivip layer 1 | 1106 | 1181 | 1196.6 | 1427.7 | **1580.8** | 1648.8 | 2131.1 | 2792.3 | 2883.4 | 2899.4 | 3017.5 | 3033.5 | Sheep^2^ |
| I14C0479 | Goat | n/a | 1105.6 | 1180.6 | 1196.6 | 1427.7 | 1580.8 | 1648.8 | 2131.1 | 2792.3 | 2883.4 | 2899.4 |  | 3093.5 | Goat^2^ |
| I14C0439 | Sheep | vivip 2 | 1105.6 |  | 1196.6 | 1427.7 | **1580.8** | 1648.8 | 2131.1 |  |  |  | 3017.5 |  | Sheep^2^ |

*1 - ZooMS currently cannot distinguish between red deer (*Cervus elaphus*), fallow deer (*Dama dama*), and European elk (*Alces alces*), BUT based on site context the most likely species is red deer.*

*2 - includes both domestic and wild species.*

**Figure S1: Scheme showing the different forms of cooking pottery found at Rakushechny Yar.**
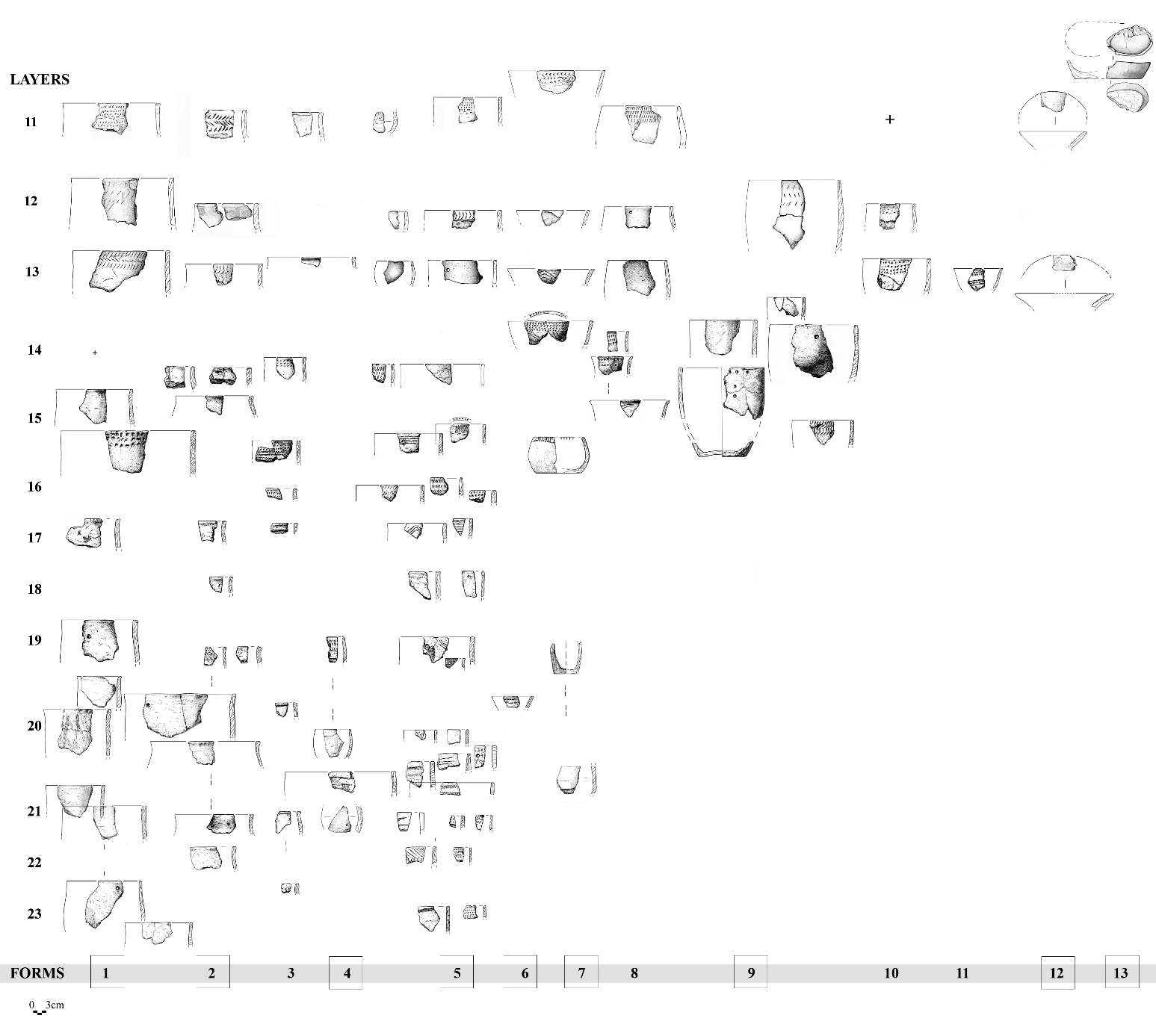


**Figure S2:** **A typical total ion current (TIC) chromatogram (a)** **of a lipid extract from the Rakushechny Yar (sample: Raku-929c-AE, acid/methanol extract) and (b) of the ceramic sample 151 (acid/methanol extract). This shows the presence of saturated fatty acids (FA), diacids (DA), branched (br), long-chain unsaturated fatty acids and cholesterol derivatives.**

**
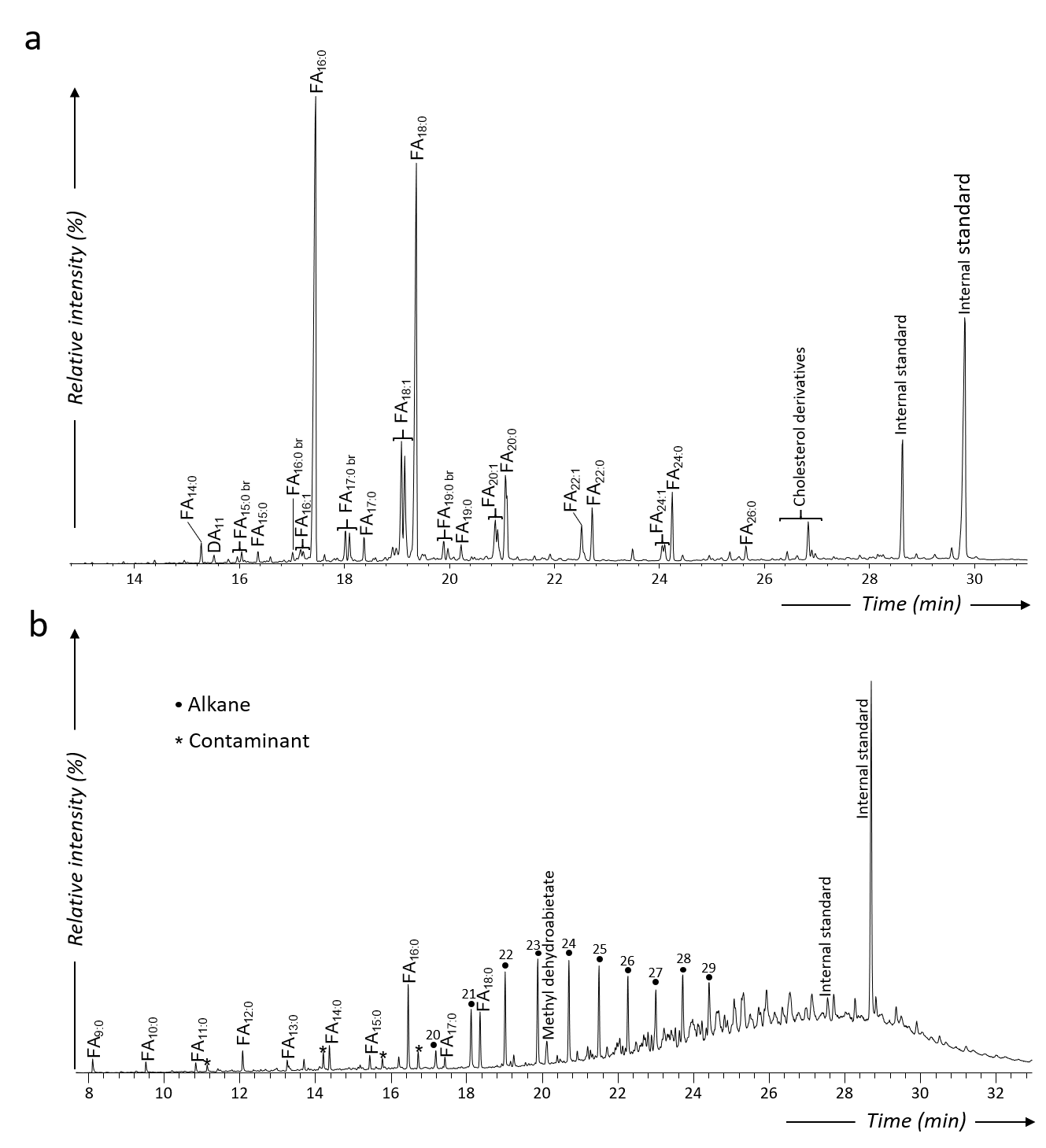
**

**Figure S3: Elongated ceramic found at Rakushechny Yar.**

**
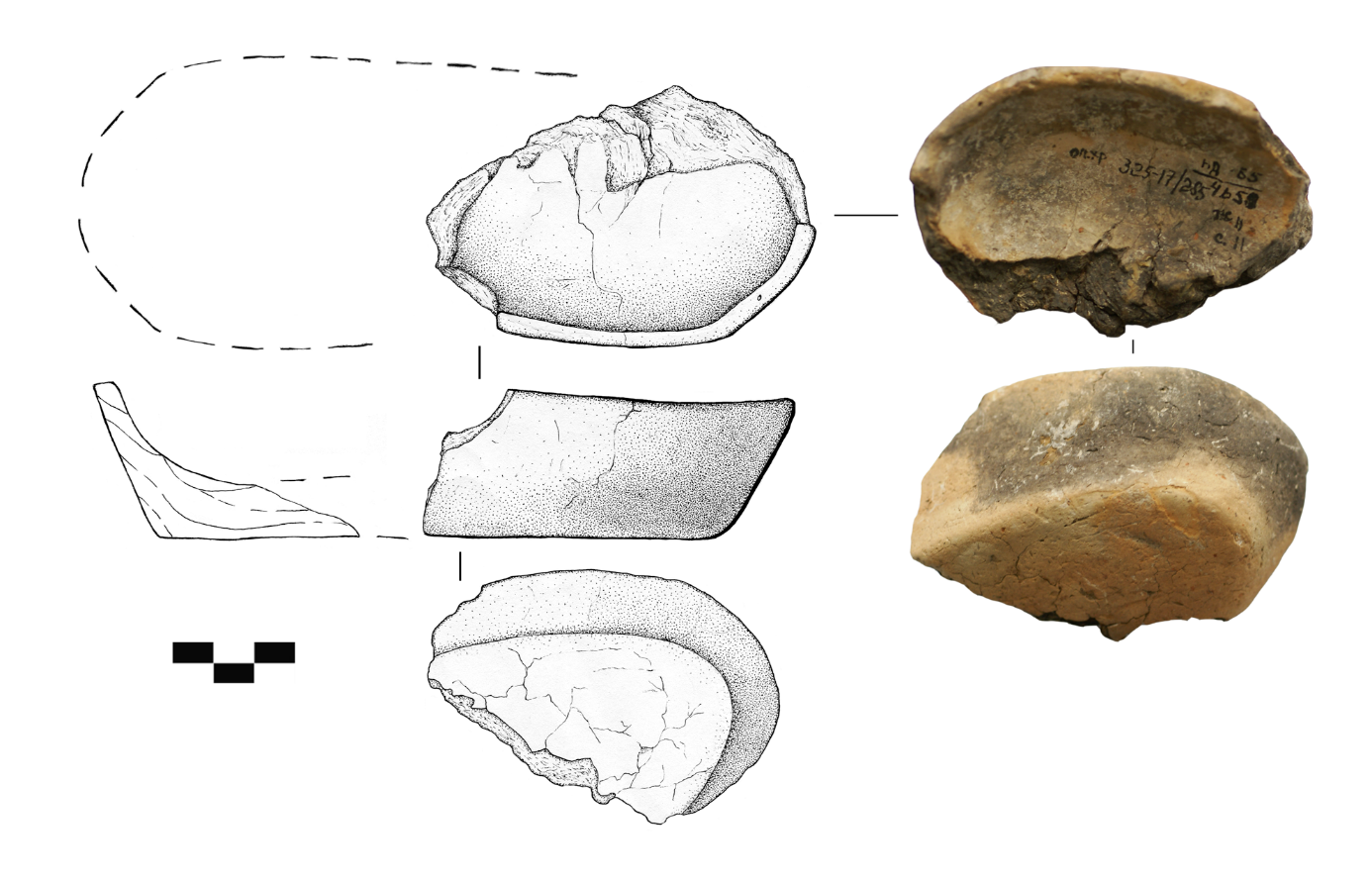
**

**Method and Materials**

1. Lipid extraction

Each sherd was first mechanically cleaned using a modelling drill to remove a few outer millimeters of the surface and then finely crushed. When available, carbonised deposits adhered on the surface were collected using a sterile scalpel and ground along with the ceramic samples.

- 1. Direct methanolic acid extraction

All the pottery and foodcrust samples were subjected to acidified methanol extraction following the established protocol [(Craig et al. 2013)](https://paperpile.com/c/wuLnGy/45BF+K5HB), as were sediments from the site. Powdered samples of ceramic and sediment (*ca.* 1 g) and foodcrusts (*ca.* 10-20 mg) were homogenised with methanol (4 mL and 1 mL, respectively) and sonicated in a water bath for 15 min. Then, concentrated sulfuric acid was added  (200 µL and 800 µL, respectively) in the vial and samples were placed in a heat block for 4 hours at 70 °C. Lipids were subsequently extracted by centrifugation (3000 rpm, 5 min) with *n-*hexane (3 x 2 mL), after which the samples were concentrated under a stream of nitrogen and, finally, directly analysed by Gas Chromatography-Mass Spectrometry (GC-MS) and Gas Chromatography-Combustion-Isotope Ratio Mass Spectrometry (GC-C-IRMS).

- 1. Solvent extraction and trimethylsilyl (TMS) derivatization

A selection of samples was subjected to solvent extraction based on published methodologies (Bondetti et al. 2020b). Briefly, the powdered samples were weighed (potsherd: 1 g; foodcrusts: 10-20 mg) and mixed with a mixture of dichloromethane-methanol (potsherd: 4 mL; foodcrusts: 2 mL; 2:1 *v/v*). Samples were ultrasonicated (3 × 15 min) to promote the extraction and next centrifuged (3000 rpm, 10 min) to facilitate the separation of the phases. In order to analyse samples by GC-MS, the total lipid extract (TLE) was derivatized using BSTFA (N, O-bis (trimethylsilyl) trifluorocetamide; 100 μl), during 1 hour at 70 °C, then evaporated and reconstituted in *n*-hexane before being analysed by GC-MS.

1. Collagen extraction from archaeological bones for isotopic analysis

Collagen was extracted from bones using the standard developed by [Longin (1971) and Brown et al. (1988), then modified in Alexander et al. (2015)](https://paperpile.com/c/WyqUz3/GnNi+HjrI+oGRn). Each sample was mechanically cleaned, weighed out (200-800 mg), immersed in HCl solution (0.6 M) and placed in the fridge, at 4 °C, until demineralization was complete. Samples were rinsed with ultra-pure water (3 times). A dilute HCl solution (pH=3) was added and the tubes were placed in a heat block set at 80 °C for 48h to gelatinise and then cooled. The samples were first filtered using polyethylene Ezee Filters (9 mL, pore size 60–90 µm; Elkay Laboratories Ltd.) to remove large unwanted particulate matter from the dissolved collagen, followed by ultrafiltration using Amicon centrifugal filters (30 kDa, Ultra-4 centrifugal filter units; Millipore, Burlington, MA, USA ) to restrict contamination and eliminate molecules larger than 30 k Da. The samples were then frozen at -20 °C for 48 hours prior to being freeze-dried in a condensing chamber held at -55°C for around 24h. The collagen extracts were then analysed by Elemental Analysis-Isotope Ratio Mass Spectrometry (EA-IRMS).

1. Collagen extraction and analysis from archaeological bones for ZooMS

ZooMS was performed similar to that outlined in Buckley et al. (2009). For each specimen, 10-30 mg of bone was sampled and immersed in 0.6 M HCl, then placed in a fridge at 4 °C to demineralize. Once demineralized, the samples were rinsed three times with 50 mM ammonium bicarbonate (AmBic, pH 8.0). A final 100 µL of AmBic was added and the samples were gelatinized at 65 °C for one hour. Following gelatinization, 50 µL of the supernatant was transferred to a new 1.5 mL Eppendorf and 0.4 µg of trypsin was added (the remaining 50 µL and residual bone material were stored at -20⁰C for possible later use). The samples were digested for approximately 18 hours at 37 °C, and then acidified to 0.1% TFA to stop the trypsin. Samples were zip tipped using 100 µL C18 tips (Millipore) following the manufacturer’s recommendations, and collagen peptides were eluted in 50 µL of a solution of 50% acetonitrile / 0.1% TFA (vol/vol). 1 µL of sample was spotted in triplicate on a Bruker ground steel MALDI plate, along with 1 µL of α-Cyano-4-hydroxycinnamic acid matrix, and allowed to air dry. Calibration standards were also included. The plate was run on a Bruker ultraflex III MALDI-ToF-MS in reflector mode with 1000 acquisitions per spot. Spectra were collected over a mass range from *m/z* 800–4000. Resultant spectra were averaged and analyzed using mMass software ([www.mmass.org](http://www.mmass.org), Strohalm et al. 2008) and compared against a database of published *m/z* markers (Buckley et al. 2009; Buckley et al. 2010; Buckley and Collins 2011; Kirby et al. 2013).

1. Instrumentation settings
   1. Gas Chromatography-Mass Spectrometry (GC-MS)

The GC-MS analyses were conducted using an Agilent 7890A series Gas Chromatography (Agilent Technologies, Cheadle, Cheshire, UK) coupled to an Agilent 5975C Inert XL mass selective detector with a quadrupole mass analyser (Agilent technologies, Cheadle, Chershire, UK). Splitless injector was used and held at 300 °C. The GC column was directly introduced in the ion source of the mass spectrometer. The ionisation and fragmentation were accomplished by electron impact (70 eV) and the mass filter was set to scan between m/z 50 and 800. All the samples were screened in full scan mode by using a DB-5 (5%-phenyl)-methylpolysiloxane column (30m, 250 µm, 0.25 µm; J&W Scientific technologies, Folsom, CA, USA). The temperature program was set at 50 °C for 2 min, followed by a temperature increase at a rate of 10 °C/min, until 325 °C where it was held for 15 min. Helium was used as carrier gas at a constant flow 3 mL/min.

All the acid extracts were also analysed on (50%-Cyanopropyl)-methylpolysiloxane DB23 column (60 m, 250 µm, 0.25 µm; J&Scientific technologies, Folsom, CA, USA) in Selected Ion Monitoring (SIM) mode. This column is particularly useful for the high-quality separation of fatty acid methyl esters (FAMEs). The temperature program was 50 °C for 2 min, which increased at a rate of 10 °C/min until 100 °C, followed by an increase to 140 °C at a rate of 4 °C/min, then a rise of 0.5 °C/min to 160 °C, and finally by 20 °C/min until it reached 250 °C where the temperature was kept for 10 min. The flow rate of the carrier gas (helium) was set at 1.5 mL/min. This SIM method enabled better detection of isoprenoid fatty acids (pristanic and phytanic acid and 4,8,12-trimethyltridecanoic acid [TMTD]) and ω-(o-alkylphenyl) alkanoic acids (APAAs) associated with aquatic resources [(Cramp and Evershed 2014)](https://paperpile.com/c/wuLnGy/mCQ2) by characterising four specific ion groups [(Shoda et al. 2017; Admiraal et al. 2018)](https://paperpile.com/c/wuLnGy/NvkF+8tZ7). Moreover, this method enables the resolution and quantification of the two natural phytanic acid diastereomers [(Lucquin](https://paperpile.com/c/wuLnGy/2NtN) [et al. 2016)](https://paperpile.com/c/wuLnGy/2NtN) lending further weight to the argument for its origin.

- 1. Gas Chromatography-Combustion-Isotope Ratio Mass Spectrometry (GC-C-IRMS)

Instruments and instrument conditions for GC-C-MS followed existing procedures [(Lucquin et al. 2018)](https://paperpile.com/c/wuLnGy/NEod+qYoq). The equipment used for measuring stable carbon isotope values of the major compounds was a Delta V Advantage isotope ratio mass spectrometer (Thermo Fisher, Bremen, Germany) linked to a Trace Ultra gas chromatograph (Thermo Fisher) with a GC Isolink II interface to oxidise all the carbon species to CO_2_. The instrument was equipped with a DB-5MS ultra-inert fused-silica column (60 m × 0.25 mm × 0.25 µm; J&W Scientific). For each sample 1 μL was injected in splitless mode at 300 °C. The carrier gas used was ultra-high-purity-grade helium with a flow rate of 2 mL/min. A parallel acquisition of the molecular data was realised by deriving a small part of the flow to an ISQ mass spectrometer (Thermo Fisher). The temperature program was 50 °C for 0.5 min, followed by a temperature rise at a rate of 25 °C/min until 175 °C, then raised 8 °C/min to 325 °C where it was held for 20 min.

Eluted products were ionized in the mass spectrometer by electron impact, and ion intensities of m/z 44, 45, and 46 were recorded by automatic calculation of the ^13^C/^12^C ratio of each peak in the extracts. The repeated measuring of standard reference gas (CO_2_), for which the isotopic composition is known, allowed the computation to be carried out with IonOS (Isoprime, Cheadle, UK). The values were reported in per mille (‰) comparative to an international standard, Vienna Pee Dee Belemnite (VPDB). A calibration curve (average R^2^ = 0.958 ± 0.048 in 8 batches) of samples, based on expected vs. measured δ^13^C values of *n*-alkanes and *n-*alkanoic acid esters international standards (Indiana A6 and F8-3 mixture), was used for each batch for calibration. Standards of *n*-alkanoic acid esters of known isotopic composition (Indiana standard F8-3, 16 measurements) were used in order to determine the accuracy and precision of the instrument. The mean ± standard deviation (S.D.) values of these were −29.95 ± 0.04‰ and −23.22 ± 0.04‰ for the methyl ester of C_16:0_ (reported mean value vs. VPDB -29.90 ± 0.03‰) and C_18:0_ (reported mean value vs. VPDB -23.24 ± 0.01‰) respectively. All the samples were analysed in duplicate and the S.D. computed (mean of S.D. 0.06‰ for C_16:0_ and 0.08‰ C_18:0_). For each batch, a standard mixture of C_16:0_ and C_18:1_ fatty acids of known isotopic composition were measured under identical conditions in order to correct the sample values taking into account the methylation of the carboxyl group, which occurred during methanolic acid extraction [(Craig et al. 2013a; Lucquin](https://paperpile.com/c/wuLnGy/45BF+ueXW) [et al. 2016)](https://paperpile.com/c/wuLnGy/45BF+ueXW). To allows comparison with the archaeological samples from the Holocene period, the δ^13^C values of the modern samples were adjusted given the variation in the atmospheric δ^13^C resulting from post-industrial carbon according to the known or estimated year of death of the animal [(Hellevang and Aagaard 2015](https://paperpile.com/c/wuLnGy/eEsm+BBR7+ueXW)).

- 1. Bulk isotope analysis - Analysis-Isotope Ratio Mass Spectrometry (EA-IRMS)

Charred residues, finely crushed, and archaeological animal collagen were weighed in duplicate (between 0.9-1.1 mg) into tin capsules and then subjected to Elemental Analysis-Isotope Ratio Mass Spectrometry (EA-IRMS). The bulk stable nitrogen (δ^15^N) and carbon (δ^13^C) isotope values were measured using protocols reported elsewhere [(Craig et al. 2007; Lucquin](https://paperpile.com/c/wuLnGy/4SEN+ueXW+NvkF) [et al. 2016)](https://paperpile.com/c/wuLnGy/4SEN+ueXW+NvkF). Instrument precision on the repeated measurements was ±0.2‰ (s.e.m.), δ^13^C, δ^15^N = [(R_sample_/R_standard_-1)] 1,000, where R = ^13^C/^12^C and ^15^N/^14^N. The measurements of international standard reference materials (IAEA 600, IAEA N2, IA Cane) were performed in each run in order to determine run accuracy. Values are given in per mill (‰) relative to the standards, Vienna Pee Dee Belemnite for δ^13^C, and air N_2_ for δ^15^N, respectively. The δ^15^N of samples with <1% N were disregarded [(Lucquin et al. 2016; Shoda et al. 2017; Lucquin et al. 2018)](https://paperpile.com/c/wuLnGy/ueXW+NvkF+qYoq).

1. Radiocarbon dating

Twenty-four AMS ^14^C dates on single mammal bone fragments, obtained in the course of a larger ongoing dating programme funded by the INDUCE project, are listed in Table S5 (below). Thirteen of these results (from samples dated at the Leibniz-Labor, Christian-Albrechts University, Kiel, laboratory code KIA-) were published by Dolbunova, et al. (2019) before the bones were identified by ZooMS at BioArCh, University of York, following methods described above. In some cases, the ZooMS identification given here supersedes the original morphometric identification.

Eleven more dates relevant to the chronology of the pottery assemblage analysed for this paper are published here for the first time. These samples were dated at the Oxford Radiocarbon Accelerator Unit, University of Oxford (OxA-), Scottish Universities Environmental Research Centre, East Kilbride (SUERC-) or the Isotope Climatology and Environmental Research Centre, Institute of Nuclear Research of the Hungarian Academy of Sciences, Debrecen (DeA-). All three laboratories apply published methods (Brock et al. 2010; Molnár et al. 2013; Dunbar et al. 2016; Major et al. 2019a; Major et al. 2019b) for collagen extraction, combustion, graphitisation and AMS measurement, whose efficacy is confirmed by long-term reproducibility of results on internal and international bone standards. Samples yielding <1% collagen by weight are rejected, as are collagen extracts with atomic C/N ratios outside the range 2.9—3.5. Conventional ^14^C ages were converted to calendar dates using OxCal v.4 (Bronk Ramsey 2009) and the IntCal13 calibration data (Reimer et al. 2013).

The new results suggest that the earliest pottery excavated in 2016-18 is no earlier than c. 5600 cal BC (Figure S4, below). Although many bones from the upper Early Neolithic layers contained no collagen and could not be dated, the few results from the stratigraphically latest early Neolithic layers – ‘trench’ layers 5 and 6 – suggest that the Early Neolithic phase was brief. Three much more recent samples from the uppermost layers (SUERC-86126; DeA-21601; SUERC-88042) must be later intrusions, or indicate that the Early Neolithic deposits were truncated. Thus, Early Neolithic pottery from the recent excavations may all date to a narrow range in the mid-6^th^ millennium (Figure S4).

Legacy ^14^C dates (Tsybryi et al. 2017; figure 3 and table 1) on samples from Early Neolithic layers appear to span a much wider range (c. 7000-5000 cal BC; Figure S4). Most of these results are probably misleading, however, due to freshwater reservoir effects. Although the dated food-crusts have not been analysed directly, it may be assumed that they contained a similar range of ingredients to those analysed for this paper, in which aquatic ingredients feature prominently. The ^14^C ages of all 10 carbonised food-crusts on sherds from Belanovskaya’s layers 15 to 20 fall between those of bulk fish-bones (SPb-1185, 8020±120 BP) and unidentified mammal bones (DeA-20972, 6462±33 BP; SPb-731, 6560±100 BP) attributed to the same layers. One of the legacy food-crust ^14^C ages (SPb-751, 6050±100 BP, from layer 11) is apparently later than the mammal bones from deeper layers, which may imply that the Early Neolithic phase at Rakushechny Yar lasted rather longer than indicated by the dates from the new excavations. Thus, the sherds from the Belanovskaya excavations analysed for this paper from layers 11—13 may date to the second half of the 6th millennium. Sherds from Belanovskaya layers 14—16, 19—21 and 23 can be dated to the middle of the 6th millennium.

Legacy ^14^C dates on bulk charcoal and an elk bone from layer 15 and below appear to be much earlier than the oldest dates from the recent excavations (Figure S4). Inconsistencies between the charcoal dates from layers 19-20, and between charcoal and animal bone dates in layer 15, suggest that much of the charcoal sampled may have been redeposited (or had a high intrinsic age), a suggestion reinforced by the new AMS date on a bone from layer 20 (DeA-20972, 6462±33 BP, 5490—5360 cal BC). The layer 23 elk bone (SPb-729, 7970±110 BP, 7180—6590 cal BC) is so much earlier than any other dated bone that its association with pottery use is questionable, particularly as there are mid-late 7th millennium ^14^C dates at the aceramic site Razdorskaya II, on the opposite bank of the Don (Tsybryi et al. 2017). Thus, the entire Early Neolithic assemblage appears to date to the mid-later 6th millennium cal BC.

**Table S6. AMS ^14^C dates on single mammal bone fragments, Rakushechny Yar. Results of samples dated in Glasgow (SUERC-) and Debrecen (DeA-) are previously unpublished; only morphometric identifications are available. ZooMS identifications (*) of samples dated in Kiel (KIA-) supersede morphometric identifications of these samples (Dolbunova** **et al.** **2019).**

| Samples ID | Bone ID | Localisation | Identification | Laboratory code | ^14^C age (BP) | δ^13^C (‰) | δ^15^N (‰) | C/N |
| --- | --- | --- | --- | --- | --- | --- | --- | --- |
| I14C0270 | 70 | 2013 trench layer 5 upper | large mammal longbone midshaft | SUERC-88042 | 2128±25 | -20.3 | 9.9 | 3.3 |
| I14C0271 | 127 | 2013 trench layer 5 upper | large mammal longbone midshaft | DeA-21601 | 4535±35 |  |  |  |
| I14C0284 | 5876 | 2018 trench layer 5 | horse tooth | SUERC-88043 | 6644±27 | -19.7 | 5.0 | 3.2 |
| I14C0281 | 5151 | 2018 trench layer 6 fireplace | bone, not determined | DeA-20971 | 6584±33 | -20.3 | 4.5 | 3.1 |
| I14C0065 | 146 | 2016 exc.2 upper vivip layer 1 | deer incisor | SUERC-86126 | 4179±28 | -18.5 | 11.5 | 3.3 |
| I14C0273 | 791 | 2016 exc.2 vivip 2 | large mammal longbone midshaft | DeA-20969 | 6634±34 | -20.1 | 5.9 | 3.2 |
| I14C0156 | 1492 | 2016 exc.2 vivip 3 | red deer phalanx 2 | OxA-39619 | 6604±26 | -19.9 | 6.7 | 3.2 |
| I14C0275 | 1620 | 2016 exc.2 vivip 3 | medium mammal longbone midshaft | DeA-20970 | 6568±33 | -20.4 | 4.6 | 3.1 |
| I14C0285 | 326 | 1966 layer 20 square M8 | bone, not determined | DeA-20972 | 6462±33 | -19.9 | 4.8 | 3.1 |
| I14C0008 | 1525 | 2016 exc.1 layer 15a upper part, square A7 | *red deer phalanx 1 | KIA-52981 | 6590±28 |  |  |  |
| I14C0009 | 1474 | 2016 exc.1 layer 15a lower part | *red deer radius | KIA-52982 | 6649±27 | -19.8 | 5.9 | 3.2 |
| I14C0010 | 1456 | 2016 exc.1 layer 15a lower part | *red deer flat bone | KIA-52983 | 6626±28 | -20.2 | 5.1 | 3.2 |
| I14C0011 | N5 | 2016 exc.1 layer 16 Unio shell #1 | *red deer long bone | KIA-52984 | 6655±28 | -20.0 | 5.1 | 3.2 |
| I14C0012 | 1218 | 2016 exc.1 layer 16 Unio shell #1 | *red deer long bone | KIA-52985 | 6632±28 | -19.2 | 6.5 | 3.2 |
| I14C0013 | 1215 | 2016 exc.1 layer 16 Unio shell #1 | *red deer rib | KIA-52986 | 6683±29 | -19.9 | 6.2 | 3.2 |
| I14C0014 | 1202 | 2016 exc.1 layer 16 Unio shell #1 | *pig scapula | KIA-52987 | 6681±28 | -20.7 | 7.9 | 3.2 |
| I14C0015 | 1719 | 2016 exc.1 layer 17 lower part | *red deer rib | KIA-52988 | 6645±27 | -19.6 | 6.0 | 3.2 |
| I14C0015 | 1676 | 2016 exc.1 layer 17 lower part | *red deer rib | KIA-52989 | 6711±27 | -19.9 | 5.4 | 3.1 |
| I14C0019 | 1776 | 2016 exc.1 layer 17 Unio 6 | *red deer splinter | KIA-52992 | 6652±28 | -19.6 | 5.4 | 3.1 |
| I14C0020 | 1708 | 2016 exc.1 layer 17 Unio 6 | *red deer pelvis | KIA-52993 | 6650±29 | -19.5 | 5.4 | 3.2 |
| I14C0021 | 1772 | 2016 exc.1 layer 17 Unio 6 | *red deer splinter | KIA-52994 | 6643±28 | -20.1 | 5.5 | 3.2 |
| I14C0022 | 1860 | 2016 exc.1 layer 17 Unio shell #2 | *pig long bone | KIA-52995 | 6666±30 | -20.5 | 7.3 | 3.2 |
| I14C0439 | ND'16 | exc. 2 vivip 2 | Sheep bone | SUERC-94518 | 5433±31 | -18.9 | 9.1 | 3.3 |
| I14C0479 | ND'19-2-N6371 | Unclear | Goat tooth | SUERC-94517 | 1855±31 | -19.6 | 7.4 | 3.3 |

**Figure S4. Calibrated radiocarbon results on Early Neolithic samples from Rakushechny Yar. Previously unpublished dates are labelled with bold text. The green band corresponds to 5600-5400 cal BC, a range which would accommodate all new dates on mammal bones (green) (Table S5). Legacy ^14^C dates from adjacent trenches (Tsybryi et al. 2017) for charred food crust (red) and total organic carbon content (grey) of pottery, and fish bone (blue) have been calibrated without accounting for potential freshwater reservoir effects. Bulk charcoal samples (black) may incorporate wood-age offsets and residual (redeposited) fragments. Within each excavation area, samples are grouped stratigraphically (earlier, deeper layers below later layers).**


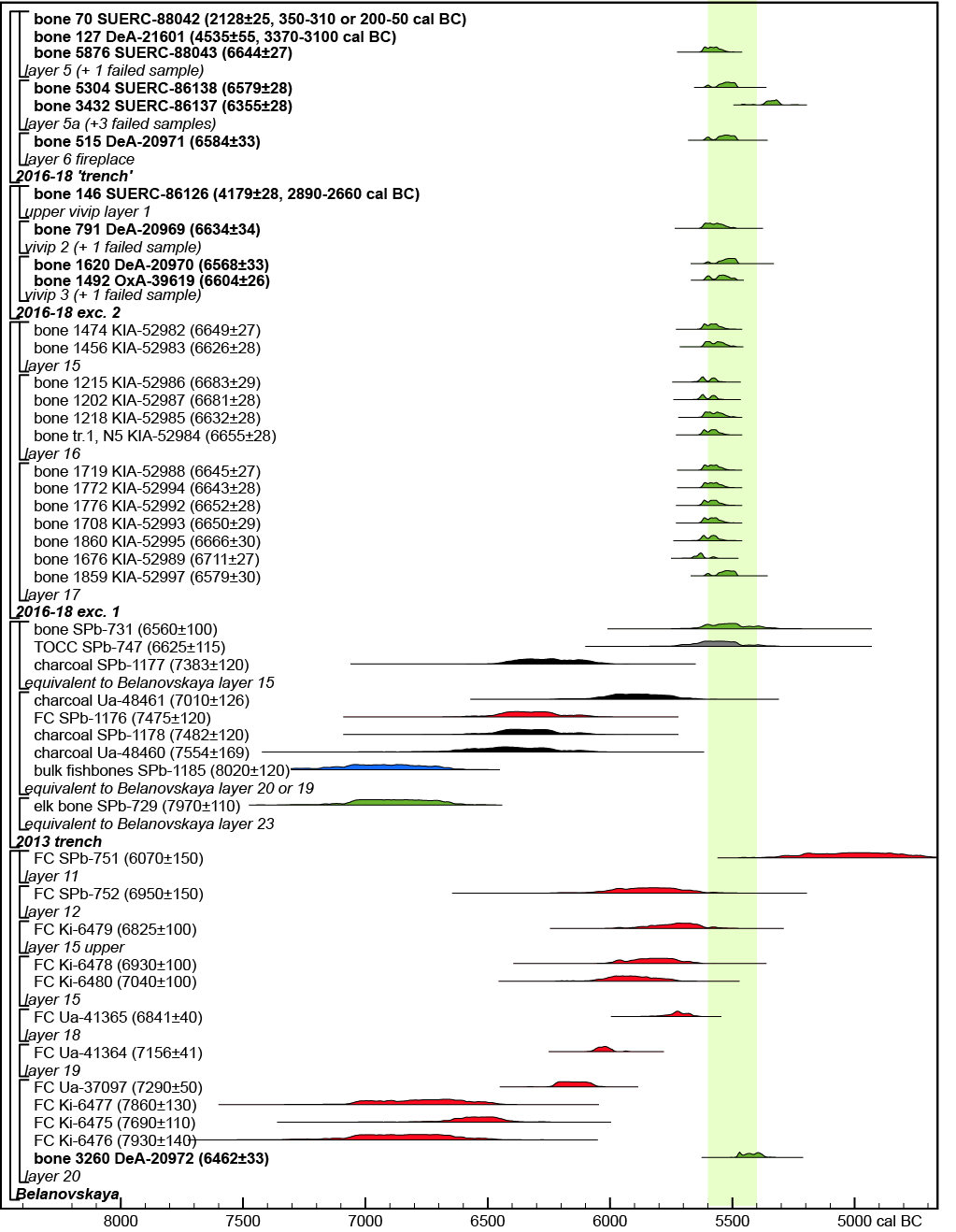


**References**

[Admiraal, M., Lucquin, A., Tersch, M. von, Jordan, P.D., Craig, O.E., 2019. Investigating the function of prehistoric stone bowls and griddle stones in the Aleutian Islands by lipid residue analysis. Quaternary Research 91, 1003–1015.](http://paperpile.com/b/5zXchd/houy)

Alexander, Michelle M., Christopher M. Gerrard, Alejandra Gutiérrez, and Andrew R. Millard. 2015. Diet, Society, and Economy in Late Medieval Spain: Stable Isotope Evidence from Muslims and Christians from Gandía, Valencia. Am J Phy. Anthropol 156: 263–73. https://doi.org/10.1002/ajpa.22647

[Bondetti M, Scott E, Courel B, Lucquin A, Shoda S, Lundy J, Labra-Odde C, Drieu L, Craig O.E. (2020a) Investigating the formation and diagnostic value of ω-(o-alkylphenyl)alkanoic acids in ancient pottery. Archaeometry](about:blank)  https://doi.org/10.1111/arcm.12631

[Bondetti M, Scott S, Lucquin A, et al (2020b) Fruits, fish and the introduction of pottery in the Eastern European plain: Lipid residue analysis of ceramic vessels from Zamostje 2. Quat Int 541:“104–114.” https://doi.org/](http://paperpile.com/b/YeFa2L/H6OT)[10.1016/j.quaint.2019.05.008](http://dx.doi.org/10.1016/j.quaint.2019.05.008)

[Brock, F., Higham, T., Ditchfield, P., Bronk Ramsey, C., 2010. Current pretreatment methods for AMS radiocarbon dating at the Oxford Radiocarbon Accelerator Unit (ORAU). Radiocarbon 52, 103-112.](http://paperpile.com/b/5zXchd/houy)

Bronk Ramsey, C., 2009. Bayesian analysis of radiocarbon dates, Radiocarbon 51, 337-360.

[Brown, T.A., Nelson, D.E., Vogel, J.S., Southon, J.R., 1988. Improved collagen extraction by modified Longin method. Radiocarbon 30, 171–177.](http://paperpile.com/b/5zXchd/v8v5)

[Buckley, M., Collins, M.J., 2011. Collagen survival and its use for species identification in Holocene-lower Pleistocene bone fragments from British archaeological and paleontological sites. Antiqua 1, e1–e1.](http://paperpile.com/b/5zXchd/Ie1b)

[Buckley, M., Collins, M., Thomas-Oates, J., Wilson, J.C., 2009. Species identification by analysis of bone collagen using matrix-assisted laser desorption/ionisation time-of-flight mass spectrometry. Rapid communications in mass spectrometry: RCM 23, 3843–3854.](http://paperpile.com/b/5zXchd/R4pm)

[Buckley, M., Whitcher Kansa, S., Howard, S., Campbell, S., Thomas-Oates, J., Collins, M., 2010. Distinguishing between archaeological sheep and goat bones using a single collagen peptide. Journal of Archaeological Science 37, 13–20.](http://paperpile.com/b/5zXchd/vM46)

[Choy, K., Potter, B.A., McKinney, H.J., Reuther, J.D., Wang, S.W., Wooller, M.J., 2016. Chemical profiling of ancient hearths reveals recurrent salmon use in Ice Age Beringia. Proceedings of the National Academy of Sciences of the United States of America 113, 9757–9762](http://paperpile.com/b/5zXchd/mY11).

[Courel B, Robson HK, Lucquin A, et al (2020) Organic residue analysis shows sub-regional patterns in the use of pottery by Northern European hunter–gatherers. R Soc Open Sci 7:e192016. https://doi.org/](http://paperpile.com/b/ehsPcr/6HIU)[10.1098/rsos.192016](http://dx.doi.org/10.1098/rsos.192016)

[Craig, O.E., Forster, M., Andersen, S.H., Koch, E., Crombé, P., Milner, N.J., Stern, B., Bailey, G.N., Heron, C.P., 2007. Molecular and isotopic demonstration of the processing of aquatic product in Northern European Prehistoric pottery. Archaeometry 49, 135–152.](http://paperpile.com/b/5zXchd/awXE)

[Craig, O.E., Steele, V.J., Fischer, A., Hartz, S., Andersen, S.H., Donohoe, P., Glykou, A., Saul, H., Jones, D.M., Koch, E., Heron, C.P., 2011. Ancient lipids reveal continuity in culinary practices across the transition to agriculture in Northern Europe. Proceedings of the National Academy of Sciences of the United States of America 108, 17910–17915.](http://paperpile.com/b/5zXchd/qRmj)

[Craig, O.E., Allen, R.B., Thompson, A., Stevens, R.E., Steele, V.J., Heron, C., 2012. Distinguishing wild ruminant lipids by gas chromatography/combustion/isotope ratio mass spectrometry. Rapid communications in mass spectrometry: RCM 26, 2359–2364.](http://paperpile.com/b/5zXchd/u12W)

[Craig, O.E., Saul, H., Lucquin, A., Nishida, Y., Taché, K., Clarke, L., Thompson, A., Altoft, D.T., Uchiyama, J., Ajimoto, M., Gibbs, K., Isaksson, S., Heron, C.P., Jordan, P., 2013. Earliest evidence for the use of pottery. Nature 496, 351–354.](http://paperpile.com/b/5zXchd/slFE)

[Cramp, L.J.E., Evershed, R.P., 2014. Reconstructing Aquatic Resource Exploitation in Human Prehistory Using Lipid Biomarkers and Stable Isotopes. In: Holland, H.D., Turekian, K.K. (Eds.), Treatise on Geochemistry. Elsevier, Oxford, pp. 319–339.](http://paperpile.com/b/5zXchd/bwkK)

Dolbunova, E.V., Tsybryi, V.V., Mazurkevich, A.N., Tsybryi, A.V., Szmańda, J., Kittel, P., Zabilska-Kunek, M., Sablin, M.V., Gorodetskaya, S.P., Hamon, C., Meadows, J., 2019. Subsistence strategies and the origin of early Neolithic community in the lower Don River valley (Rakushechny Yar site, early/middle 6th millennium cal BC): First results, Quaternary International.

Dunbar, E., Cook, G.T., Naysmith, P., Tripney, B.G., Xu, S., 2016. AMS ^14^C Dating at the Scottish Universities Environmental Research Centre (SUERC) Radiocarbon Dating Laboratory – Corrigendum, Radiocarbon 58, 233-233.

[Hellevang, H., Aagaard, P., 2015. Constraints on natural global atmospheric CO_2_ fluxes from 1860 to 2010 using a simplified explicit forward model. Scientific Reports 5, e17352.](http://paperpile.com/b/5zXchd/YDI4)

Horiuchi, A., Miyata, Y., Kamijo, N., Cramp, L., Evershed, R.P., 2015. A Dietary Study of the Kamegaoka Culture Population during the Final Jomon Period, Japan, Using Stable Isotope and Lipid Analyses of Ceramic Residues. Radiocarbon 57, 721-736.

[Jørkov, M.L.S., Heinemeier, J., Lynnerup, N., 2007. Evaluating bone collagen extraction methods for stable isotope analysis in dietary studies. Journal of Archaeological Science 34, 1824–1829.](http://paperpile.com/b/5zXchd/hcoi)

[Kirby, D.P., Buckley, M., Promise, E., Trauger, S.A., Holdcraft, T.R., 2013. Identification of collagen-based materials in cultural heritage. The Analyst 138, 4849–4858.](http://paperpile.com/b/5zXchd/6lmb)

[Longin, R., 1971. New method of collagen extraction for radiocarbon dating. Nature 230, 241–242.](http://paperpile.com/b/5zXchd/2bre)

[Lucquin, A., Gibbs, K., Uchiyama, J., Saul, H., Ajimoto, M., Eley, Y., Radini, A., Heron, C.P., Shoda, S., Nishida, Y., Lundy, J., Jordan, P., Isaksson, S., Craig, O.E., 2016a. Ancient lipids document continuity in the use of early hunter–gatherer pottery through 9,000 years of Japanese prehistory. Proceedings of the National Academy of Sciences 113, 3991–3996.](http://paperpile.com/b/5zXchd/LwlU)

[Lucquin, A., Colonese, A.C., Farrell, T.F.G., Craig, O.E., 2016b. Utilising phytanic acid diastereomers for the characterisation of archaeological lipid residues in pottery samples. Tetrahedron Letters 57, 703–707.](http://paperpile.com/b/5zXchd/Xq8x)

[Lucquin, A., Robson, H.K., Eley, Y., Shoda, S., Veltcheva, D., Gibbs, K., Heron, C.P., Isaksson, S., Nishida, Y., Taniguchi, Y., Nakajima, S., Kobayashi, K., Jordan, P., Kaner, S., Craig, O.E., 2018. The impact of environmental change of the use of early pottery by East Asian hunter-gatherers. Proceedings of the National Academy of Sciences 115, 7931–793.](http://paperpile.com/b/5zXchd/5k1m)

Major, I., Dani, J., Kiss, V., Melis, E., Patay, R., Szabó, G., Hubay, K., Túri, M., Futó, I., Huszánk, R., Jull, A.J.T., Molnár, M., 2019a. Adoption and evaluation of a sample pretreatment protocol for radiocarbon dating of cremated bones at HEKAL, Radiocarbon 61, 159-171.

Major, I., Futó, I., Dani, J., Cserpák-Laczi, O., Gasparik, M., Jull, A.J.T., Molnár, M., 2019b. Assessment and development of bone preparation for radiocarbon dating at HEKAL, Radiocarbon 61, 1551-1561.

Molnár, M., Rinyu, L., Veres, M., Seiler, M., Wacker, L., Synal, H.A., 2013. EnvironMICADAS: A mini 14C AMS with enhanced gas ion source interface in the Hertelendi Laboratory of Environmental Studies (HEKAL), Hungary, Radiocarbon 55, 338-344.

[Outram, A.K., Stear, N.A., Bendrey, R., Olsen, S., Kasparov, A., Zaibert, V., Thorpe, N., Evershed, R.P., 2009. The earliest horse harnessing and milking. Science 323, 1332–1335.](http://paperpile.com/b/5zXchd/dccN)

[Pääkkönen, M., Bläuer, A., Evershed, R.P., Asplund, H., 2016. Reconstructing food procurement and processing in Early Comb Ware Period through organic residues in Early Comb and Jäkärlä Ware Pottery. Fennoscandia Archaeologica XXXIII, 57–75.](http://paperpile.com/b/5zXchd/WC1T)

Reimer, P.J., Bard, E., Bayliss, A., Beck, J.W., Blackwell, P.G., Bronk Ramsey, C., Buck, C.E., Cheng, H., Edwards, R.L., Friedrich, M., Grootes, P.M., Guilderson, T.P., Haflidason, H., Hajdas, I., Hatte, C., Heaton, T.J., Hoffmann, D.L., Hogg, A.G., Hughen, K.A., Kaiser, K.F., Kromer, B., Manning, S.W., Niu, M., Reimer, R.W., Richards, D.A., Scott, E.M., Southon, J.R., Staff, R.A., Turney, C.S.M., van der Plicht, J., 2013. IntCal13 and Marine13 radiocarbon age calibration curves 0-50,000 years cal BP, Radiocarbon 55, 1869-1887.

Tsybryi, A., Tsybryi, V., Dolbunova, E., Mazurkevich, A., Kulkova, M., 2017. Radiocarbon chronology of Neolithic in the Lower Don and north-eastern Azov Sea, Documenta Praehistorica 44, 204-222.

[Richards, M.P., Hedges, R.E.M., Molleson, T.I., Vogel, J.C., 1998. Stable Isotope Analysis Reveals Variations in Human Diet at the Poundbury Camp Cemetery Site. Journal of Archaeological Science 25, 1247–1252.](http://paperpile.com/b/5zXchd/aU4s)

[Shoda, S., Lucquin, A., Ahn, J.-H., Hwang, C.-J., Craig, O.E., 2017. Pottery use by early Holocene hunter-gatherers of the Korean peninsula closely linked with the exploitation of marine resources. Quaternary Science Reviews 170, 164–173.](http://paperpile.com/b/5zXchd/eg1r)

[Spangenberg, J.E., Jacomet, S., Schibler, J., 2006. Chemical analyses of organic residues in archaeological pottery from Arbon Bleiche 3, Switzerland – evidence for dairying in the late Neolithic. Journal of Archaeological Science 33, 1–13.](http://paperpile.com/b/5zXchd/zakY)

[Strohalm, M., Hassman, M., Kosata, B., Kodícek, M., 2008. mMass data miner: an open source alternative for mass spectrometric data analysis. Rapid communications in mass spectrometry: RCM 22, 905–908.](http://paperpile.com/b/5zXchd/INMP)
